# Development of InDel markers for interspecific hybridization between hill pigeons and feral pigeons based on whole-genome re-sequencing

**DOI:** 10.1038/s41598-022-27147-1

**Published:** 2022-12-30

**Authors:** Jin-Yong Kim, Jung Eun Hwang, Soo Hyung Eo, Seung-Gu Kang, Jeong Chan Moon, Jung A Kim, Jin-Young Park, Junghwa An, Yonggu Yeo, Jongmin Yoon

**Affiliations:** 1 Research Center for Endangered Species, National Institute of Ecology, Yeongyang, South Korea; 2 Department of Forest Science, Kongju National University, Yesan, Chungnam, South Korea; 3 National Institute of Biological Resources, Incheon, South Korea; 4 Conservation and Health Center, Seoul Zoo, Gwacheon, South Korea

**Keywords:** Ecological genetics, Invasive species, Genetic hybridization, Genetic markers

## Abstract

Interspecific hybridization occurs among birds, and closely related sister taxa tend to hybridize at a high rate. Genomic hybridization markers are useful for understanding the patterns and processes of hybridization and for conserving endangered species in captivity and the wild. In this study, we developed genomic hybridization markers for the F1 progeny of the sister taxa feral pigeons (*Columba livia* var*. domestica*) and endangered hill pigeons (*Columba rupestris*) (family Columbidae). Using whole-genome re-sequencing data, we performed genome-wide analysis for insertion/deletion (InDel) polymorphisms and validated using primers. We conducted polymerase chain reaction (PCR) and agarose gel electrophoresis to identify species-specific InDels. We produced eight F1 hybrids of hill and feral pigeons, and their samples were tested by re-performing analyses and sequencing using 11 species-specific InDel polymorphisms. Eight InDel markers simultaneously amplified two DNA fragments from all F1 hybrids, and there was no abnormality in the sequencing results. The application of genomic tools to detect hybrids can play a crucial role in the assessment of hybridization frequency in the wild. Moreover, systematic captive propagation efforts with hybrids can help control the population decline of hill pigeons.

## Introduction

Successful interspecific mating can be infrequent because of reproductive barrier diversity^1^. Nevertheless, interspecific hybridization does occur in nature among both plants and animals, albeit at low frequencies^2^. Novel genomic data have revealed multiple incidences of multispecies hybridization^3^. Interspecific hybridization can be an important generator of genetic diversity, which drives adaptation and speciation under changing or fluctuating environments^4–6^. However, it can also negatively impact the maintenance of species boundaries^7–9^. In birds, various phenotypic traits—such as mating, foraging behavior, and migration—can act as pre-mating barriers or extrinsic post-zygotic barriers to successful interspecific mating^9^. In addition, low levels of social bonding and migration are associated with a lack of hybridization^10^. In recent years, climate change and anthropogenic habitat disturbance have been implicated in the dismantling of some barriers to hybridization^11–14^. As such, issues of hybridization in nature pose challenges to biological and phylogenetic species concepts and conservation practices.

Hybrid detection using genomic data has potential applications in the conservation of endangered species that exhibit hybridization and introgression. It can also be used to measure the rate of hybridization, differentiate natural vs. human-caused hybridization, and in the genetic management of species to improve their adaptability. In birds, interspecific hybrids have been identified in the natural populations of various species using different DNA markers, including Forbes’ parakeet, buntings, and chickadees (using mtDNA and microsatellites)^15–17^; partridges (using RAPD)^18^; warblers and owls (using AFLP)^19–21^; and flycatchers, woodpeckers, and eagles (using microsatellites and single nucleotide polymorphisms [SNPs])^22–24^. At present, third-generation DNA markers based on SNP and insertions/deletions (InDels) have gained popularity^25,26^. InDel markers have been widely used to differentiate hybrids^25,27^, as they can be genotyped with simple procedures based on size separation^28^.

Another advantage of InDel markers is the minuscule chance of two InDel mutations of exactly the same length occurring at the same genomic position. Therefore, shared InDels can be seen as representing identity by descent^28^. Recent advances in DNA sequencing technology have led to the reference genomes of various species becoming publicly accessible^29^.Consequently, InDel polymorphisms can be easily identified by mapping new re-sequencing data to reference genomes^27^. Recent studies on hybridization have advanced the identification and widespread availability of multiple nuclear markers. These markers can be used to directly link interspecific hybridization and its evolutionary significance with the adaptability of hybridizing species (e.g., their fitness or selection pressures), rather than with evolutionary noise^30^.

Two variants of rock pigeons—domestic (*Columba livia*) and feral (*Columba livia* var*. domestica*)—are among the most intensively studied birds worldwide. Wild rock pigeons (*C. livia*) are native to the Old World, whereas domestic pigeons with diverse forms have been produced through long-term artificial selection by humans^31^. Several domestic varieties have also been going feral worldwide^32^. In particular, racing breeds of domestic pigeons have made extensive contributions to feral populations^29,33^. The frequency of hybridization is known to be higher among sister species than among non-sister species^34^. Gene flow from feral pigeons to native populations has reduced the genetic originality of rock pigeons, leading to overlaps in morphology and behavioral ecology^35^. Furthermore, interspecific hybridization between two closely related species—feral pigeons and hill pigeons (*Columba rupestris*)—has been reported in some regions, including in Northern India, Southern Siberia, and South Korea^36,37^. This is a cause for concern, as the hybridization has been accompanied by a loss of *C. rupestris* population^36–38^. However, the patterns and processes of interspecific hybridization between the two species remain unclear because of the lack of contemporary genomic tools^39^.

In the present study, we developed agarose-resolvable InDel markers through whole-genome re-sequencing (WGS) to differentiate hill pigeons from feral pigeons and their F1 hybrid. We used these markers to verify the hybrid risk in wild populations as follows: (1) we identified InDel polymorphisms between hill and rock pigeons (i.e., domestic and feral pigeons) using WGS; (2) we conducted experimental validation of species-specific InDel polymorphisms through agarose gel electrophoresis using native populations in South Korea (i.e., hill and feral pigeons); and (3) we developed hybrid markers between hill and feral pigeons. Polymorphisms in these species-specific InDel regions were re-investigated (through agarose gel electrophoresis) using four pairs of hill and feral pigeons and their F1 hybrids. On the basis of our results, we discuss the conservation perspectives of endangered species that exhibit interspecific hybridization with target species.

## Results

### Identification of InDel polymorphisms between hill pigeons and rock pigeons

A total of 112,638,908 and 841,269,524 clean reads were generated from seven hill pigeons and seven rock pigeons (Table 1). The clean reads were mapped to the reference genome assembly (Clive_1.0) using Burrows–Wheeler Aligner (BWA), and 88–94.45% of the reads from hill pigeons and rock pigeons were mapped with a depth of 8.61–45.67, respectively (Table 1). The overall genome coverage from hill pigeons and rock pigeons was 98.61–99.63% (Table 1), and genome-wide analysis revealed 226,030–814,877 InDels (Table 2). InDels occurred most frequently in intron regions (43.83%), followed by intergenic regions (24.13%) (Table 3). A total of 67 primers with an InDel length of > 34 bp (HM 1–HM 67) (Table S1) were used for experimental validation of species-specific InDel polymorphisms.

**Table 1:** Summary of the original sequencing data of seven hill pigeons and seven feral/domestic pigeons.

Species	Sample ID	Total bases (GB)	≥ Q30 (%)	Clean reads	Mapped ratio (%)	Coverage ratio (%)	Depth
Hill pigeon	NIBRGR0000605614	32.9	90.7	308,291,226	91.3	99.5	24.0
	NIBRGR0000605618	35.9	90.7	336,074,660	90.6	99.5	25.3
	NIBRGR0000605610	34.8	90.0	324,104,102	90.7	99.5	24.7
	NIBRGR0000605613	39.2	90.4	367,097,674	91.4	99.5	28.2
	NIBRGR0000617910	35.2	90.3	329,308,000	92.0	99.5	25.8
	NIBRGR0000605620	33.9	90.3	316,816,196	88.4	99.5	22.8
	SRS346866	22.5	89.3	213,721,788	90.6	99.2	14.6
Feral pigeon	NIBRGR0000605604	35.5	90.7	333,955,758	94.4	99.6	27.1
	SRS346884	16.0	92.2	168,163,172	90.0	99.2	10.7
	SRS346865	36.9	86.7	326,767,858	91.0	99.6	24.4
	SRS346899	11.2	92.7	117,376,882	90.2	98.7	8.1
	SRS346873	14.4	85.4	144,312,392	91.2	98.8	10.0
	SRS346877	12.6	88.4	128,638,908	89.1	98.6	8.6
	Clive_1.0	127.3	85.8	841,269,524	88.0	99.9	45.7

**Table 2:** InDel polymorphisms identified in the genome sequences of seven hill pigeons and seven feral/domestic pigeons.

Species	Sample ID	Ref homo	Alt homo	No. of InDels
Hill pigeon	NIBRGR0000605614	573,905	231,756	805,661
	NIBRGR0000605618	560,700	244,724	805,424
	NIBRGR0000605610	551,728	241,624	793,352
	NIBRGR0000605613	554,981	259,896	814,877
	NIBRGR0000617910	571,252	235,040	806,292
	NIBRGR0000605620	535,554	233,064	768,618
	SRS346866	451,230	226,154	677,384
Feral pigeon	NIBRGR0000605604	278,226	430,118	708,344
	SRS346884	264,744	183,556	448,300
	SRS346865	283,893	198,898	482,791
	SRS346899	230,743	77,117	307,860
	SRS346873	253,676	90,466	344,142
	SRS346877	242,258	92,549	334,807
	Clive_1.0	27,384	198,646	226,030

*Ref homo* a relationship that is homozygous with the reference (feral pigeon) group, *Alt* *homo* a relationship that is homozygous with the alteration (hill pigeon) group.

**Table 3:** Types of InDel polymorphisms identified in the genome sequences of seven hill pigeons and seven feral/domestic pigeons.

Type	Count	Percentage (%)
Downstream	3,443,130	9.54
Exon	972,718	2.69
Intergenic	8,712,664	24.13
Intron	15,824,569	43.83
None	3,385,177	9.38
Splice site acceptor	3066	0.01
Splice site donor	2844	0.01
Splice site region	58,222	0.16
Transcript	433	0.00
Upstream	3,324,084	9.21
UTR 3′	290,996	0.81
UTR 5′	86,444	0.24

*UTR* untranslated region.

### Experimental validation of species-specific InDel markers for hill pigeon and feral pigeon

To identify the species-specific InDel regions between the two species, we used 16 hill pigeons and 14 feral pigeons. All hill pigeons used in the experiment were confirmed as hill pigeons based on their feather phenotype (Fig. S1). Using 67 primers, we performed PCR and agarose gel electrophoresis with randomly selected individuals: hill pigeon 4 (or 2) and feral pigeon 5 (or 2). Primer pairs that amplified different base pairs between the two species (Fig. S2) were selected. As a result, species-specific InDels between the two species were identified in 17 out of 67 primer pairs (Fig. S2). PCR and agarose gel electrophoresis were re-performed using the 17 selected primers for 16 hill pigeons and 14 feral pigeons (Fig. S3). Finally, 11 species-specific InDel markers were identified (Fig. S3).

### Experimental validation of InDel markers for F1 hybrids between hill pigeons and feral pigeons

**Figure 1:**
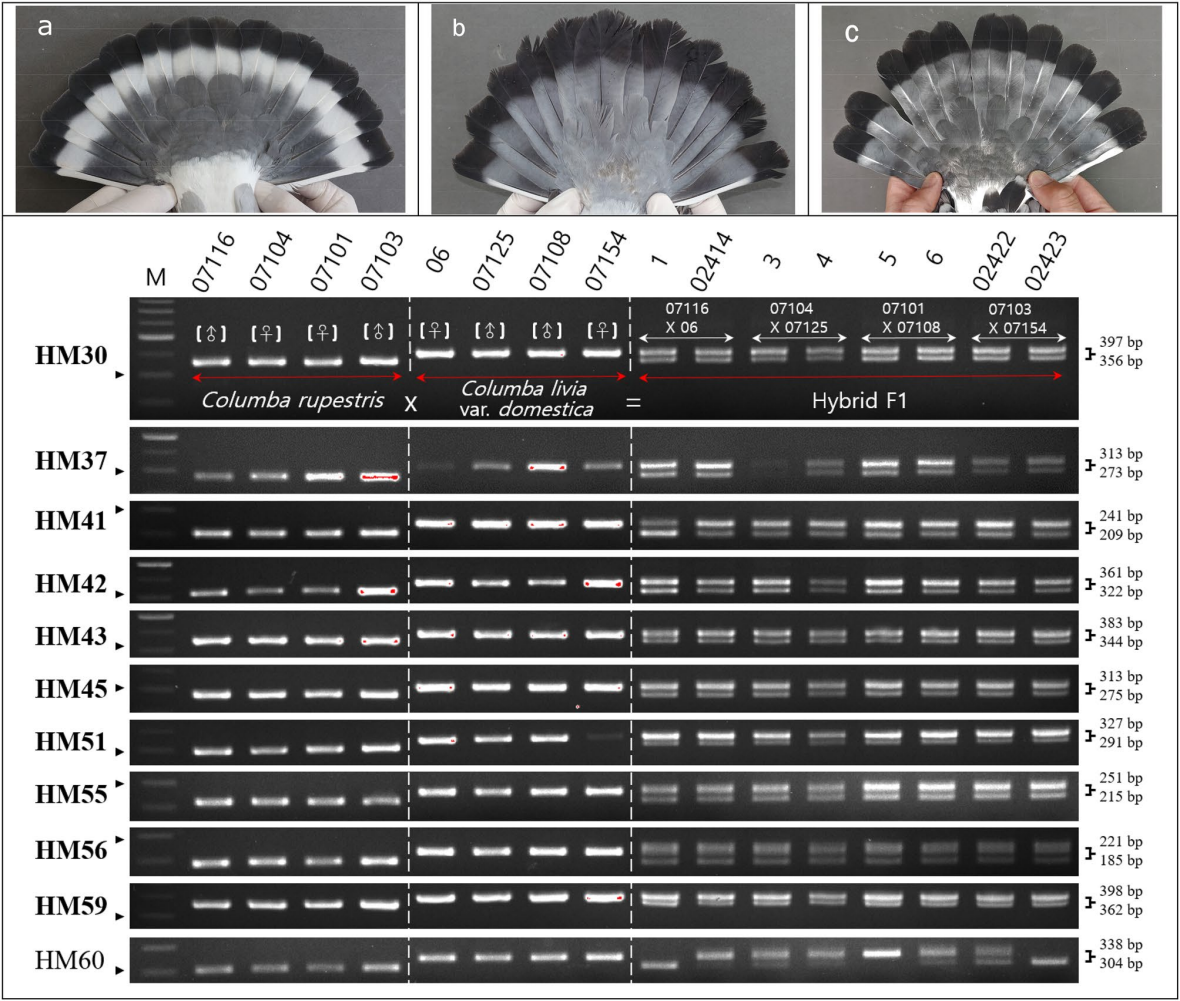
Testing of 11 InDel regions to develop the markers of hybridization between two wild populations (*Columba rupestris* and *C. livia* var*. domestica*) and their hybrid using agarose gel electrophoresis. The names of accepted markers are shown in bold. Black arrows indicate 300 bp. Representative phenotypes (i.e., white band in tail feathers) of pigeons. Phenotypes are shown for (**a**) a hill pigeon (*C. rupestris*), (**b**) a feral pigeon (*C. livia* var*. domestica*), and (**c**) their F1 hybrid. Groups of gels are cropped from different gel images and original images are included in Fig. S5.

**Figure 2:**
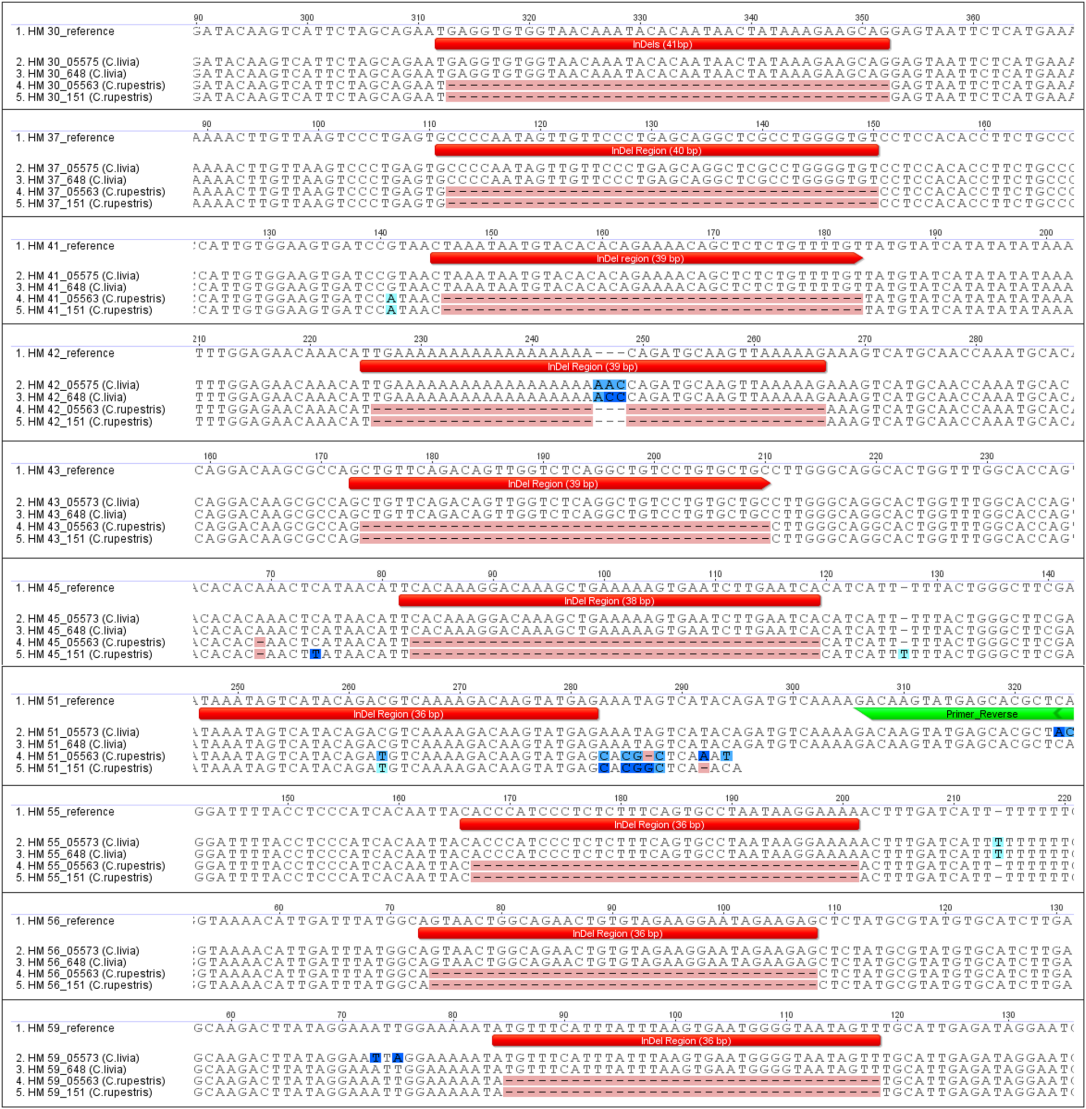
Sequences of 10 selected InDel markers to identify the F1 hybrids between hill and feral pigeons. Data are shown for two randomly selected individuals in the two species.

Eleven of the species-specific InDel markers were used to develop the hybrid markers. A total of eight F1 hybrids were produced using four individuals of hill pigeon and feral pigeon each (Fig. S4). Five out of eight hybrid F1 individuals died in the brooding stage. Therefore, the phenotype of only three individuals could be confirmed. Using the 16 individuals, 11 species-specific InDel markers were tested. For 10 (HM30, HM37, HM41, HM42, HM43, HM45, HM51, HM55, HM56, and HM59) out of the 11 markers, two DNA fragments (hill and feral pigeon) were simultaneously amplified from all F1 individuals (Fig. 1). In contrast, HM60 amplified 304-bp or 338-bp fragments in some individuals of hybrid F1 (Fig. 1). Ten of the selected hybrid InDel markers were sequenced using the PCR products of two randomly selected individuals of each species. The sequencing results of HM30, HM37, HM41, HM43, HM45, HM55, HM56, and HM59 matched (Fig. 2) with the InDel regions identified during primer design (Table S1). However, in HM42, a mutation occurred in the feral pigeon (Fig. 2), and the sequencing results did not match those obtained during primer design (Table S1). Furthermore, in HM51, the position of the InDels did not match that of the InDel regions identified during primer design (Fig. 2; Table S1).

## Discussion

The rock pigeon (*C. livia*) has a long history of hybridization with its sister taxa^37^. Taxonomically, rock pigeons include domestic pigeons and free-living feral pigeons^40^. Feral pigeons (*C. livia* var*. domestica*) are not a distinct species, but a variety of rock pigeons^40^. The wild ancestors of feral pigeons were first domesticated approximately 1000 years ago, and hybridization has occurred among domestic pigeons throughout their history of domestication^41^. Subsequently, domesticated pigeons began escaping captivity and were termed “feral”^40^. Feral pigeons have been introduced worldwide by humans (The World Bird Database: https://avibase.bsc-eoc.org/avibase.jsp?lang=EN). Such artificial introductions can cause overlaps in the distribution areas of species, and pre-zygotic barriers (e.g., life cycle, behavior, and distribution) can be disrupted among closely related species. The two study species, hill and feral pigeons, have similar characteristics of inhabiting man-made structures. However, feral pigeons mainly inhabit urban areas, and hill pigeons inhabit forest areas. Occasionally, feral pigeons invade man-made structures in forest areas where hill pigeons live, resulting in hybridization. Hybrids between feral pigeons and native populations in the Columbidae family have recently been reported in North America^42^ and South Korea^36^. The hybrid markers that evolved throughout this complicated history were evaluated in this study.

Prior to experimental validation, we confirmed the phenotype of all individuals used in the experiment. All hill pigeons used in this study were collected from Gurey-gun and Goheung-gun in South Korea, where interspecific hybridization was not detected using mtDNA^36^, and the phenotype also reflected the characteristics of hill pigeons (Figs. S1, S4). In contrast, the feral pigeons showed various phenotypes, and it was not possible to confirm their species-specific phenotype. However, the white tail-band across the black tail was clearly distinguished in the phenotype between the two species (Fig. S4). The tail pattern of F1 hybrids showed a clear tendency toward mixing the phenotypes of the two species, with the gray background of the feral pigeon’s traits and a rare white band of hill pigeon’s traits. This was consistent with the characteristics of individuals suspected to be hybrids using mtDNA in a previous study^36^.

To develop interspecific markers, species-specific InDel regions were identified in 11 of the 67 primers (Fig. S3). We tested 11 species-specific InDel markers and performed PCR, agarose gel electrophoresis, and sequencing analysis using these eight F1 hybrids and their eight parents (i.e., four pairs). The results of agarose gel electrophoresis from the F1 hybrid were expected to show two DNA fragments from each parent. Although HM60 clearly distinguished the two species in the parental generation, some offspring did not inherit all of the parental characteristics (Fig. 1). We assumed that this was either because of allelic dropouts or null alleles. This phenomenon is more likely to occur when primers do not perfectly match the flanking sequences and fail to amplify one or both alleles of a diploid individual^43^. The 10 selected InDel markers (except HM60) were sequenced using two randomly selected individuals from the two species (Fig. 2). Through this process, we identified whether the expected InDels matched the sequences obtained during experimental validation (PCR and gel-loading). We confirmed that HM42 was unstable because of the occurrence of mutations in the InDel region. Moreover, HM51 was excluded from the final marker list because InDels did not occur in the expected region. In total, we developed eight InDel markers (HM30, HM37, HM41, HM43, HM45, HM55, HM56, and HM59) for F1 hybrids between hill and feral pigeons.

The genomic evaluation of interspecific hybridization within sister taxa is a conservation management tool for rare species that are close to extinction because of low genetic diversity, inbreeding depression, or poorly adapting phenotypes in rapidly changing environments^44^. The occurrence and function of hybridizations in nature may be underestimated, and the effects of anthropogenic habitat disturbance should also be considered from the perspective of conservation biology^12,45,46^. Among birds, hybrids exhibit higher fitness than parental species in fluctuating environments^4^. Hybridization between species can also assist species range expansion^47^. Nevertheless, the survival probability of fertile hybrids seems to be low under strong pre-zygotic selection in birds, although hybridizing species may not be rare in the wild^7^. Ecosystem-scale alterations in the physical habitat (e.g., clear-cutting, road and city construction and urbanization, eutrophication, and irrigation) and the introduction of invasive species have been on-going in the wild, resulting in a breakdown of reproductive barriers (i.e., increasing hybridization or introgression)^12^. The overall proportion of feral pigeons cohabiting with hill pigeons was approximately 20% within the three known colonies (i.e., 140 individuals) in South Korea, where the small numbers of interspecific hybrids have been reported to invade and compete with feral pigeons.

In the present study, we developed eight InDel markers (HM30, HM37, HM41, HM43, HM45, HM55, HM56, and HM59) using WGS. Our findings can be applied to the conservation of hill pigeons, which have recently shown a rapid decline in South Korea^36^. In addition, the method used in this study (i.e., selecting InDel markers using WGS) may be used as a precedent for the discrimination of hybrids occurring in other animal taxa. Genomic tools for hybrid detection are expected to play a crucial role in the assessment of hybridization frequency in the wild. Therefore, our method can be a convenient approach for hybrid detection; however, it is expected that better results can be obtained if the structure analysis method using SNP^48^ is additionally employed to confirm and evaluate the hybrid ratio of individuals other than F1. Future studies are needed to elucidate the process of interspecific hybridization in the presence of reproductive barriers. In addition, the systematic captive propagation of the hybrids of hill pigeons and feral pigeons can help mitigate the population decline of hill pigeons.

## Methods

### Study system

The hill pigeon is closely related to the rock pigeon and snow pigeon (*C. leuconota*). The two subspecies of the study species are recognized as *C. r. turkestanica* (western form) and *C. r. rupestris* (eastern form)^37^. The hill pigeon is similar in appearance to the rock pigeon in terms of size and plumage but is differentiated by its tail pattern. Specifically, hill pigeons have a broad, white tail-band across the black tail and a white patch on the back (Fig. 1, Fig. S1). This white-banded tail pattern is similar to that of the snow pigeon. Recently, researchers have reported a decline in the populations of hill pigeons in India and South Korea, primarily because of invasion, nest-site competition, and interspecific hybridization by feral pigeons. Nevertheless, hill pigeons have been designated a non-endangered species on the basis of their overall distribution^36,37^. The frequency of hybridization between the two sister taxa is stable (3–10% of the population), and there are some ethological barriers to potential random mating (e.g., breeding asynchrony and habitat isolation)^37^. Historical records from 1948 indicate that hill pigeons (but not rock pigeons or feral pigeons) were commonly found in palace grounds and coastal river cliffs in the Korean Peninsula^49^. However, recent reports indicate that there are only three breeding populations with less than 100 individuals in South Korea^36^. Accordingly, the taxon has been recommended for priority conservation with interspecific hybridization (with *ca.* 20% cohabiting feral pigeons) in South Korea.

### DNA sampling

All samples were collected in South Korea between 2015 and 2022. Hill pigeons were captured in Gurye-gun and Goheung-gun, which are their known habitats^36^. Feral pigeons were captured from among individuals that had infiltrated the habitat of hill pigeons and caused hybridization. The captured individuals were transferred to the Research Center for Endangered Species of the National Institute of Ecology (NIE) and National Institute of Biological Resources (NIBR), South Korea. The phenotypes of the birds were identified, and blood samples were collected. Following this, the birds were either used for captive propagation or released back to the collection site^36^. Feathers or blood samples of six hill pigeons and one feral pigeon were used to extract DNA for WGS (Table 4)^50^. In a previous study that used the genome sequences of the samples in Table 4, all samples were clearly divided into hill and rock pigeon groups through both principal component analysis and non-synonymous SNP phylogenetic analysis, and it was confirmed that previous introgression did not occur between the two sample groups^51^. Furthermore, additional blood samples were collected from 20 hill pigeons, 18 feral pigeons, and 8 F1 hybrids (Figs. S1, S4). From these samples, DNA was extracted from these samples and analyzed by agarose gel electrophoresis. These hill pigeons were captured in Gurye and Goheung-gun, South Korea, and a previous study confirmed their distinction from rock pigeons through a haplotype network analysis using mtDNA^36^. The F1 hybrids of the two species consisted of eight chicks obtained from four pairs of parents. In order to produce F1 hybrids, one pair of hill and feral pigeon were placed in a total of four cages using each two female and two male of each species. And natural breeding was induced by providing a sufficient amount of grains. The brooding process after egg hatching also induced a natural feeding process by the parents. The study design was approved by the Research Experimental Ethics Committee of NIE (NIEIACUC-R-2021-019). And all experiment were performed in accordance with the ARRIVE guidelines, and carried out Accordance with relevant guidelines and regulations.

**Table 4:** Whole-genome re-sequencing sources of seven hill pigeons (*Columba rupestris*) and seven feral/domestic pigeons (*C. livia* var*. domestica*).

Species	Subspecies or breeds	Sample ID	Accession name	Origin	Source
Hill pigeon	–	NIBRGR0000605614	SRR19175034	South Korea	This study
	–	NIBRGR0000605618	SRR19175033	South Korea	This study
	–	NIBRGR0000605610	SRR19175032	South Korea	This study
	–	NIBRGR0000605613	SRR19175031	South Korea	This study
	–	NIBRGR0000617910	SRR19175030	South Korea	This study
	–	NIBRGR0000605620	SRR19175029	South Korea	This study
	–	–	SRS346866	–	NCBI
Feral pigeon	var*. domestica*	NIBRGR0000605604	SRR19175028	South Korea	This study
	English pouter	–	SRS346884	–	NCBI
	Fantail	–	SRS346865	–	NCBI
	Parlor roller	–	SRS346899	–	NCBI
	Scandaroon	–	SRS346873	–	NCBI
	Chinese owl	–	SRS346877	–	NCBI
	Danish tumbler	–	Assembly Clive_1.0	–	NCBI

### DNA extraction and high-throughput DNA sequencing

The collected samples were stored at − 30 °C before DNA extraction, and DNA was extracted using the DNeasy Blood & Tissue Kit (Qiagen, Hilden, Germany), following the manufacturer’s protocol. The quality of extracted DNA was evaluated by electrophoresis, and the DNA concentration measured using a Nanodrop system (Thermo Fisher Scientific, Waltham, MA, USA). Genomic DNA (200 ng) was sheared into ~ 500-bp fragments using the Covaris system (Covaris, Woburn, MA, USA), and libraries were constructed using a library kit (Illumina, San Diego, CA, USA). High-throughput paired-end sequencing was performed on an Illumina NovaSeq platform.

### Mining of InDel polymorphisms from the genomes of hill and rock pigeons

To identify InDel polymorphisms between hill pigeons and rock pigeons (domestic and feral pigeons), we explored the reference genome of the rock pigeon^29^ downloaded from the National Center for Biotechnology Information (NCBI) (accession no: Assembly Clive_1.0). The genome sequences of five breeds of domestic pigeon and one hill pigeon^29^ were also obtained from NCBI (Table 4). Genomic DNA isolated from the six hill pigeons and one feral pigeon was also used for analysis, and InDel polymorphisms between two groups of 14 individuals (Table 4) were identified using the reference genome (assembly Clive_1.0). Clean reads were obtained by trimming the ends of low-quality reads (< Q30) (Table 1). The Sickle software was used to trim the sequences (https://github.com/najoshi/sickle). The cleaned reads were aligned to the reference genome with the Burrows–Wheeler Aligner (BWA 1.7.10-r789). Quality control of FastQ files was performed using the FastQC software (http://www.bioinformatics.babraham.ac.uk/projects/fastqc/). InDels were filtered and called using the Genome Analysis Tool Kit (GATK) version 3.1 (https://gatk.broadinstitute.org/hc/en-us). InDel polymorphisms were extracted under the following conditions: homozygous within the groups (hill pigeon or rock pigeon) and heterozygous between the two groups (hill pigeon and rock pigeon).

### Primer design, PCR amplification, and sequencing

The online Primer3-Plus tool (http://www.bioinformatics.nl/cgi-bin/primer3plus/primer3plus.cgi) was used to design PCR primers for the locations of InDel polymorphisms between hill and rock pigeons. The parameters were as follows: amplicon size, 150–400 bp; primer melting temperature (Tm), 48–60 °C; and 40–60% primer GC content. Based on these parameters, the primers that included a total of 1703 InDel polymorphisms > 20 bp in length were identified. To select primers that can be visually confirmed as conveniently as possible after gel loading, the InDel polymorphisms were listed in order of the longest. Subsequently, a total of 67 primers were selected, that included 18 primers with InDels > 50 bp, 19 primers with InDels > 40 bp, and 30 primers with InDels > 34 bp (HM 1–HM 67) (Table S1), which were used to investigate species-specific InDel polymorphisms between hill and feral pigeons through agarose gel electrophoresis. PCR amplifications for all samples were carried out (PCR volume, 30 µL) using 2 × Lamp Taq PCR Pre-Mix (Biofact, Daejeon, South Korea). The PCR thermal profile of each primer has been described in Table S2. All PCR products were checked on 2% agarose gels and visualized using ultraviolet light at 300 nm on Gel Doc XR plus (Bio-Rad Laboratories, Inc., Hercules, CA, USA). The sequencing was performed by a commercial sequencing service (Macrogen Inc., Seoul, South Korea) on two randomly selected individuals from two species (hill pigeon and feral pigeon).

## Supplementary Information

Supplementary Information.

## Data Availability

These WGS data have been submitted to the NCBI databases under accession numbers SRR19175034, SRR19175033, SRR19175032, SRR19175031, SRR19175030, SRR19175029, SRR19175028; the address is http://www.ncbi.nlm.nih.gov/sra/PRJNA837397.
