# Supplementary material for: Development of InDel markers for interspecific hybridization between hill pigeons and feral pigeons based on whole-genome re-sequencing
Source: Sci Rep. 2022 Dec 30;12:22618. doi: 10.1038/s41598-022-27147-1 (PMC9803650; doi:10.1038/s41598-022-27147-1)
Supplement: Supplementary file 1 — Supplementary Information. [file 41598_2022_27147_MOESM1_ESM.pdf]

Development of InDel markers for interspecific hybridization between hill pigeons and feral pigeons based on whole-genome re-sequencing

Jin-Yong Kim[1], Jung Eun Hwang[1], Soo Hyung Eo[2], Seung-Gu Kang[1], Jeong Chan Moon[1], Jung A Kim[3], Jin-Young Park[3], Junghwa An[3], Yonggu Yeo[4], Jongmin Yoon[1,*]

Supplementary Table S1. Information of primer and InDel sequences.

Primer name	Forward and reverse primers (5′-3′)	Product size (bp)	InDel sequence
HM 1	GTCTCCTTTGAGGGGAATACAC<br>TACTTCCAAAGACAGGCTGC	340	GCCAATGATAGGACACGGGGCAATGTGTACAAACGGAAAAACAGGAGGGACAA
HM 2	CTTGGATAACTACAGAGCAGGG<br>CCCGATATGGTACCCTTTGT	338	ATAAAAAAAAATAGTCATTTTTCAGGGGAAAAAAATACCAAAGAGAAAAAGTGTAAT<br>CTGAAAAATATTAGCATACCTGGATTGG
HM 3	GTTTCTACAGAACACCAGACCC<br>ATAGTGACTCTTCCCAGACACG	357	CTAAGACACAGGCCAGGACGTGCTGTTACTCGCACTCCACACGCACTACAGCAGACG<br>TTTTACTCACGATACACCACTGTGCAGATTCGTGTCTGGGA
HM 4	CTTCCCTCTCTCTTCATCAGTG<br>CATAGGAAGGGCAGTACATCTC	354	TTTCATGGTATTATTCACACTCTTGCCAAAGAGTTCACTGACGTGCAAAGTTTGGAGT<br>TTGTCCCG
HM 5	GAAGTTAGGCAGTGTACGTTCC<br>CCTTTGAAATGTCGTGGC	377	GCAACTAATTCCTGTAGAACCTATTGGAAGTTCCAGGGCCACGACATTTC
HM 6	GTAAGAGCTGAGCAATACACCC<br>AGCTCAGTATTGCAGAGAGAGG	361	TCCCTTGTCAAAATCTTGTCTTCTTCTGATTTGCATTACCACCCACCAACATGC
HM 7	CTTGTAGCTCAGAAGCAGTAGGG<br>ACTCAGTGGACTCCTGATGTCT	397	TATCTCAAATGCAGAGACATCCTTTTTCTTTGAAGCACCATCCACTTTAAAA
HM 8	CAGGTCAGTTAAGATGGAGGAG<br>GTCTCTTCCACGAGGTACAATC	395	ACCTGCAGCCCATGGAGGACCACACTGGAGCAGACACCCACCTGCAGCCCATGGAC<br>ATTGGAGCACCTGCAGCCCATGGAGGACCACATTGGAGCAGACACCCAC
HM 9	ACCATGGTCTGACAAGAGAGC<br>CAGCACAACAAACCTGCAC	388	ATGGTATTTCAGATACTTGTCCTAATGATGTGCAGGTTTGTTGTAAAGAGAAACTAGT<br>AAAAAG
HM 10	CTATCTTGACAGCTAAGTCGGG<br>TACTTGTAGGTCTGGGGACAAC	371	TGAGTCCCAAAGATAAAAATAGAAAAGTATTATGTCCCAAAGATAAGTAAGAAACG<br>TCGAGGTGTGAAAATACTTTTAGA
HM 11	GGTGACCCTGTTATACATAGCC<br>GAGAAGACAGCTGGGAAAGTC	318	AAGGCAGGCACCAGCGCGGAGGATGGTGCTGCTCAGCCAGGGGAGCCCC
HM 12	GTCTCCTTTGAGGGGAATACAC<br>TACTTCCAAAGACAGGCTGC	256	ACGGAGAAAAAAAAGAAAACGCACGTGGTTATGATACAAATTGCAGCCTGTCTTTG
HM 13	ACCTCACCTCACATCAACTCTC<br>GGGTGTTATCCAGGAGAGAAAG	198	TGACGAGGACTGCAGGAAACACCATTTGCTTGATGTCCAAAGGGCAGGGCAAC
HM 14	AGATCCTGGAACCTACTGTCCT<br>CATCTCACTCTCGGGATCTATG	252	AGCCCCATAGATCCCAGAACCTGCTGTCCCAAGAGACCCAGGCGTTCAGGCCCCATA<br>GATCCTGGCCCCACAAGTCCCT

HM 15	GGAGAGGAAGATCTATAGAGGAGC GATGATCTTGAGGGTCTCTTCC	364	TTGTACAGATTAAATGACCTTCCTGGTAAGACGCTCCAGTATCAGCAATCTGGGGATT AA
HM 16	GACAGCCTGAGTTCTAATGACC CAATCTGCCTTGACATCCAC	180	ACAAACTATAAAAAAGGACTTTTATGGAGAATTGATGATTCCTTGACAAATAGTTTT GTGGATGTCAAGGCAGATTGTTCTAAGAAAAGTAGAAAATATAGCAG
HM 17	CTTTGCTGGTCCCTGAAGT GGGGACAACGAAACGTTAG	272	TGTTTGGTGGCCCCTAATGTTTGGTTGTCCCCAAGTTTTGGTGGCCCCAAGGCTTTGG TGGTTCCTGAGGTTTGCTGGCCACAAAA
HM 18	GATGGTATCTGTGATGTCACCC GGGTTGTCCCTTAAGTCTGAGT	352	GGGCTTGGGGACAGTCCTGGGCTTGGGGACAGTTCTGGGTCTGGGGACAGTTTGAGG TTTGGGGACAATTCAA
HM 19	GTTACGTATCCACAGACAGCAG GACCCTGAAGGTAAGTTGCAGT	341	GCAAGAAACTACAACTTACCTTCAGGGCTCCTGTGTGACCCTGAAAAATTTGTCCCTG TCCCCATGTCTAAAGCCCCAATCTGGAACATGAAGAAAAAAGCTCTGCTTCTTTCATG GGTGGGTGATGAACATT
HM 20	GTTCAGTCTGGGAACAATGG CTAGTCAATGGACTGGAGTTGG	250	TAATGAAAGGATCAGAGAATCACGGAATCGACTGGGTTGGA
HM 21	CATTGACTCAGGCTTTCACC GAACTAGGAGTCTGGATCTAGGG	383	CAGCAGGCCTTATGGGACAGAGTGAGGTCCAGATCACAACAA
HM 22	GTGTATGTGGACTCACAGGATG GGTGGATGTTGGAGATCAGT	351	TACTGAAATTCTTACAATTATTTTAAAACTTTATTTTCAGGGTATTTTA
HM 23	GGTACAAAACCAAGTGTGGG CTGTGATGTTAGTTACGCCCTC	232	AACTCAATCTTCAGCCAAGTGACCTTGGGAATTAATAGAAAT
HM 24	ACACTCAACCTCTGAGTAGGGAG CCATACCGTTGCTTTACGG	328	GTGTTTTAGACCTGAAATTGTCGTTTTAAGGTACACCTTCTTCTTCAC
HM 25	AGAGCAGAGATTCCCCTGTAG CTGCTACTCCTTCCTTCTCAAG	359	GGAGGTCCATGGTGGAGCAGAGATCCCACTGCAGCCCATC
HM 26	GTCATAGGGACACCTTACATGG CCCACAAGGTTCTCATGGT	352	AGGGGACCTTGTGGGGTCATGGGGACACCATCACATGGTCAT
HM 27	TCTGGTCTGTAAGAGACACTGC GCACTAACCCATGTCCCTAAG	383	GGGAGGTTTAGATTGGACATTAGGAAAATATTCAAGTTGTGCCAGC
HM 28	GTGCAGGGAAGCAACACTAT CTGGCTGACCTTCCTATCTACAG	261	AGTCAAAGAAGGAAGATTACTTTCAAGATCAAGGATCCTTGGGGTCT
HM 29	AGGTGTACACAGGAGACTCACAG TAGGGTACCTATGATGAGGCAG	383	AACTGTTGTAGCAGTTTATCGCTACAACGTGTGCTACTGAAGC
HM	TTGCTCGGATGATTGGAC	397	TGAGGTGTGGTAACAAATACACAATAACTATAAAGAAGCAG

30	GTATCCACTTACTGTGCACTGGC		
HM 31	AATCTCCTCTCCCTCATAGCAG<br>GACACATCACATCCACTCCAG	297	TCTGGGGTGGATGTACTACAGGGTGGATGTGTTCTGGAGTGGATGTGTC
HM 32	GAGACCTGACAAGCTGACAGAC<br>CTACTCCTAAGTTCAGTGGAGGC	382	GCTTAGGAAATAACTGGGGGTGGGCTGGCTGGGAGCCTCCACTGAA
HM 33	GGCAACACAGACAGAAGAACAG<br>CTGTGGAAGTAGCGTGTAGTACC	275	TACCCATGTTTGCTTTTTATACCGTATTAACAAAAGTAACCAGG
HM 34	GGGGTACAAGACCAAGATAAGC<br>GTATCTGTATGTTCAGCCCTGC	150	AAAAGGTCAACTTGACTGTAACACTGACAGAACTGGAGTGGG
HM 35	GTCTCACCTCGTGTGACAGATAC<br>GAAGTACCTGGAAAGGCTGAAG	344	GTTCAGCTTGGTTTCTACCAAGCTTTGTCTGTGCAAAACTGGT
HM 36	CAGAGTCCCAGACACTATGACAC<br>CTCTCCAGGCTCAAGGAATAAG	288	TCAAGTATTCCTTGAGCCTGAAGAGCAGCAGAGAGAACAAC
HM 37	GCTAGCTGTGTCTTTGGTAGGT<br>CTACAGGAGTTGGAGTCAGAGAC	313	GCCCCAATAGTTGTTCCCTGAGCAGGCTCGCCTGGGGTGT
HM 38	CACGCAGTCTACAGAAGGACTA<br>CTTGAACCTGGAACCTGCT	261	TTTCTTAAAAGCATTGTGTTTTGAAAGAACAATGGGAAA
HM 39	CCTAAGTGAGAATGGGAGACAG<br>GGGAGAGAGAGCACATATTCAC	367	AGCTTAAAAAAAAAAAAAAAAAAAAAAAAAGGCAGGCAG
HM 40	CTGTTGGCTCCTAAAGACTGCT<br>CTGTTGACAGGGAACTCCAG	312	TTTGCACTGCACCCTTACACTGCCCTGCACTGCACTGCG
HM 41	GTCAGATTATCAGTGTGAGGGG<br>CGGTGTATAAAGCTACCCTGTG	241	CTAAATAATGTACACACAGAAAACAGCTCTCTGTTTTGT
HM 42	TGCAGTCACCACTACCTACTTG<br>CTTCTCTCAAGTCTTTCCAGCC	361	TTGAAAAAAAAAAAAAAAAAACAGATGCAAGTTAAAAAG
HM 43	GTTAGTACTCCTCCTGTCCCATC<br>ATAGTGATCCACGACTACCTGC	383	GCTGTTCAGACAGTTGGTCTCAGGCTGTCCTGTGCTGC
HM 44	TACTTGGACCCACTCAGTAAGC<br>GTCACTTGTCCCATACACCTCT	199	CATAAAAGTAACCATTGGACATGGAGTACAATGCTATT
HM 45	ACAGAGAGGCAGAGAGAGAAGTC<br>AAGTACGTGGTGTAGAACTGGC	313	TCACAAAGGACAAAGCTGAAAAAGTGAATCTTGAATCA
HM	AGGTGTGCTGTTAGGTTAGGG	153	CTAAATCCTGCCTAAGCAGTTCTAAACAGTAATTCATT

46	ACCAACAGAACAGGTCAGGA		
HM 47	GTCACAGCTACCAGTTGTCTCAC CTATGCCTGCTGTCTACTTCTG	346	CTCCTGCAAAAGATTCCTTGCAGGTAAATATGTCAAAA
HM 48	GAATAGGAAGGGGTCTCCTAAC GATCTGTCTGCATCTCTGCTC	304	TAAGAGCCCAGTATGTAATAACTGAAAATCTTTCTGA
HM 49	GTTATCACTCATCTGAGCACCC ATAGGCAAGACAGACAGGACAG	393	CTTGACAGTTTAGCTTCCAAGTCCAGACTCTAGTGCT
HM 50	CTGTCCTGGCAGATCTTCAT TCTCTAACAGAGTCTGGGTCGT	382	ACTGACTGCTCAAGTAGTGTGTTATGCTGACTTCTC
HM 51	GCTATTCAGGAACACAGTCTCC GTTGAGCGTGCTCATACTTGTC	327	TAAATAGTCATACAGACGTCAAAAGACAAGTATGAG
HM 52	AGAACAGACACACACACAGAGG GTCCTGGTGTAACGTAGGAGTAG	193	CACACACAGAGGGCAAGACCAGACGCACACCCCAGT
HM 53	GCTGTGAAGTCTCTTGCCAT GTACCTCAACATCTCCCTGATG	180	TTCTGCCCCACCACTAAACGGGTGATTCTTAGGAGG
HM 54	GCCACAGTGAAGGATAGAATGC CTAAGTCTCTCTTCCTTTCCCC	391	CACCGGGTTGCTTATATTTTCAAAGGCTATAAGGAG
HM 55	CCCCTGAGTGCAGGAGTATAA TCTACCACATCCTCTGTCTTCC	251	CACCCATCCCTCTCTTTCAGTGCCTAATAAGGAAAA
HM 56	CCTGAGACCTCAAGTCCTGTAT CAGCTGTGCAGAACTGAAAC	221	AGTAACTGGCAGAACTGTGTAGAAGGAATAGAAGAG
HM 57	TAGATGCTCCCCTGTTTACCAC GGTGTCTGTACCCTGCTGTAAT	333	GTTGGGTACAAGATACCCACCTAACCTGCACCTACA
HM 58	CTGACATGTTCTCTGCTGCTTC ACAGTGGTTTGACCTGTACTCC	239	TGTTTCATTTAGAATGAAAACACAGGAGGTGGAAG
HM 59	GGGTGCTTTGTATCATCTGC CAGGATCCAATGAAGACCAC	398	ATGTTTCATTTATTTAAGTGAATGGGGTAATAGTT
HM 60	TTGGGCAGGTTTATGCAC TTGGAGCCTCTTTGCTTC	238	TAAACTATTATGAACTATTTGCTTATAAACTTATA
HM 61	TATGGACCAAGAAGAGGGACTC CATCAACACCTGAGATGTACCC	381	AGCACTATAGTGGAGATTTACATTAAGAAAAAAAT
HM	TATGTGAGGCCACAGAAAGC	363	CATGAATTACTTTTTACCTTATCTTGCTAATAGAA

62	GTCCTGGTGAGAAGCATAAACC		
HM 63	CGTCTGGTCTCATCCAAAAG<br>TAGTGGGTGAACAATCCAGC	398	ATATAATAATGGTTCCATATAATATAATATGGTTC
HM 64	CACACAGGAGCAAAGCATTC<br>GATACCATTTCGCAGAGGAG	334	GTTGGAGAATATAGTATCCCGCACTCCATATTTAA
HM 65	CACACAGGAGCAAAGCATTC<br>GATACCATTTCGCAGAGGAG	334	GTTGGAGAATATAGTATCCCGCACTCCATATTTAA
HM 66	CAATGGCTACTGATGCTACC<br>TGTGTAGACCCTGTGTTCACC	237	AAATGCTTTGATTGGTTAAAGACTACACATAAAAG
HM 67	TAGATGGTGCCTAAAAGCCC<br>GCTCATTGAGGACTAGAGGAGA	373	GACTTTAATATATGCTGCTAAACTTCACTCTGTGT

Supplementary Table S2. PCR conditions for primers used in this study

Primer	Pre-denaturation	Replication cycle				Final Extension
		Denaturation	Annealing	Extension	Cycles	
HM 1	95°C, 2:00	95°C, 0:20	57°C, 0:30	72°C, 0:30	35	72°C, 5:00
HM 2	95°C, 2:00	95°C, 0:20	57°C, 0:30	72°C, 0:30	35	72°C, 5:00
HM 3	95°C, 2:00	95°C, 0:20	57°C, 0:30	72°C, 0:30	35	72°C, 5:00
HM 4	95°C, 2:00	95°C, 0:20	52°C, 0:30	72°C, 0:30	35	72°C, 5:00
HM 5	95°C, 2:00	95°C, 0:20	57°C, 0:30	72°C, 0:30	35	72°C, 5:00
HM 6	95°C, 2:00	95°C, 0:20	57°C, 0:30	72°C, 0:30	35	72°C, 5:00
HM 7	95°C, 2:00	95°C, 0:20	57°C, 0:30	72°C, 0:30	35	72°C, 5:00
HM 8	95°C, 2:00	95°C, 0:20	52°C, 0:30	72°C, 0:30	35	72°C, 5:00
HM 9	95°C, 2:00	95°C, 0:20	57°C, 0:30	72°C, 0:30	35	72°C, 5:00
HM 10	95°C, 2:00	95°C, 0:20	57°C, 0:30	72°C, 0:30	35	72°C, 5:00
HM 11	95°C, 2:00	95°C, 0:20	57°C, 0:30	72°C, 0:30	35	72°C, 5:00
HM 12	95°C, 2:00	95°C, 0:20	57°C, 0:30	72°C, 0:30	35	72°C, 5:00
HM 13	95°C, 2:00	95°C, 0:20	57°C, 0:30	72°C, 0:30	35	72°C, 5:00
HM 14	95°C, 2:00	95°C, 0:20	51°C, 0:30	72°C, 0:30	35	72°C, 5:00
HM 15	95°C, 2:00	95°C, 0:20	57°C, 0:30	72°C, 0:30	35	72°C, 5:00
HM 16	95°C, 2:00	95°C, 0:20	57°C, 0:30	72°C, 0:30	35	72°C, 5:00
HM 17	95°C, 2:00	95°C, 0:20	52°C, 0:30	72°C, 0:30	35	72°C, 5:00
HM 18	95°C, 2:00	95°C, 0:20	57°C, 0:30	72°C, 0:30	35	72°C, 5:00
HM 19	95°C, 2:00	95°C, 0:20	57°C, 0:30	72°C, 0:30	35	72°C, 5:00
HM 20	95°C, 2:00	95°C, 0:20	60°C, 0:30	72°C, 0:30	35	72°C, 5:00
HM 21	95°C, 2:00	95°C, 0:20	60°C, 0:30	72°C, 0:30	35	72°C, 5:00
HM 22	95°C, 2:00	95°C, 0:20	51°C, 0:30	72°C, 0:30	35	72°C, 5:00
HM 23	95°C, 2:00	95°C, 0:20	60°C, 0:30	72°C, 0:30	35	72°C, 5:00
HM 24	95°C, 2:00	95°C, 0:20	51°C, 0:30	72°C, 0:30	35	72°C, 5:00
HM 25	95°C, 2:00	95°C, 0:20	52°C, 0:30	72°C, 0:30	35	72°C, 5:00
HM 26	95°C, 2:00	95°C, 0:20	60°C, 0:30	72°C, 0:30	35	72°C, 5:00
HM 27	95°C, 2:00	95°C, 0:20	60°C, 0:30	72°C, 0:30	35	72°C, 5:00
HM 28	95°C, 2:00	95°C, 0:20	60°C, 0:30	72°C, 0:30	35	72°C, 5:00
HM 29	95°C, 2:00	95°C, 0:20	60°C, 0:30	72°C, 0:30	35	72°C, 5:00
HM 30	95°C, 2:00	95°C, 0:20	49°C, 0:30	72°C, 0:30	35	72°C, 5:00
HM 31	95°C, 2:00	95°C, 0:20	49°C, 0:30	72°C, 0:30	35	72°C, 5:00
HM 32	95°C, 2:00	95°C, 0:20	54°C, 0:30	72°C, 0:30	35	72°C, 5:00
HM 33	95°C, 2:00	95°C, 0:20	60°C, 0:30	72°C, 0:30	35	72°C, 5:00
HM 34	95°C, 2:00	95°C, 0:20	52°C, 0:30	72°C, 0:30	35	72°C, 5:00
HM 35	95°C, 2:00	95°C, 0:20	51°C, 0:30	72°C, 0:30	35	72°C, 5:00
HM 36	95°C, 2:00	95°C, 0:20	60°C, 0:30	72°C, 0:30	35	72°C, 5:00
HM 37	95°C, 2:00	95°C, 0:20	53°C, 0:30	72°C, 0:30	35	72°C, 5:00

HM 38	95°C, 2:00	95°C, 0:20	56°C, 0:30	72°C, 0:30	35	72°C, 5:00
HM 39	95°C, 2:00	95°C, 0:20	56°C, 0:30	72°C, 0:30	35	72°C, 5:00
HM 40	95°C, 2:00	95°C, 0:20	52°C, 0:30	72°C, 0:30	35	72°C, 5:00
HM 41	95°C, 2:00	95°C, 0:20	58°C, 0:30	72°C, 0:30	35	72°C, 5:00
HM 42	95°C, 2:00	95°C, 0:20	52°C, 0:30	72°C, 0:30	35	72°C, 5:00
HM 43	95°C, 2:00	95°C, 0:20	52°C, 0:30	72°C, 0:30	35	72°C, 5:00
HM 44	95°C, 2:00	95°C, 0:20	56°C, 0:30	72°C, 0:30	35	72°C, 5:00
HM 45	95°C, 2:00	95°C, 0:20	59°C, 0:30	72°C, 0:30	35	72°C, 5:00
HM 46	95°C, 2:00	95°C, 0:20	56°C, 0:30	72°C, 0:30	35	72°C, 5:00
HM 47	95°C, 2:00	95°C, 0:20	56°C, 0:30	72°C, 0:30	35	72°C, 5:00
HM 48	95°C, 2:00	95°C, 0:20	56°C, 0:30	72°C, 0:30	35	72°C, 5:00
HM 49	95°C, 2:00	95°C, 0:20	56°C, 0:30	72°C, 0:30	35	72°C, 5:00
HM 50	95°C, 2:00	95°C, 0:20	56°C, 0:30	72°C, 0:30	35	72°C, 5:00
HM 51	95°C, 2:00	95°C, 0:20	51°C, 0:30	72°C, 0:30	35	72°C, 5:00
HM 52	95°C, 2:00	95°C, 0:20	56°C, 0:30	72°C, 0:30	35	72°C, 5:00
HM 53	95°C, 2:00	95°C, 0:20	56°C, 0:30	72°C, 0:30	35	72°C, 5:00
HM 54	95°C, 2:00	95°C, 0:20	51°C, 0:30	72°C, 0:30	35	72°C, 5:00
HM 55	95°C, 2:00	95°C, 0:20	52°C, 0:30	72°C, 0:30	35	72°C, 5:00
HM 56	95°C, 2:00	95°C, 0:20	51°C, 0:30	72°C, 0:30	35	72°C, 5:00
HM 57	95°C, 2:00	95°C, 0:20	56°C, 0:30	72°C, 0:30	35	72°C, 5:00
HM 58	95°C, 2:00	95°C, 0:20	56°C, 0:30	72°C, 0:30	35	72°C, 5:00
HM 59	95°C, 2:00	95°C, 0:20	49°C, 0:30	72°C, 0:30	35	72°C, 5:00
HM 60	95°C, 2:00	95°C, 0:20	52°C, 0:30	72°C, 0:30	35	72°C, 5:00
HM 61	95°C, 2:00	95°C, 0:20	56°C, 0:30	72°C, 0:30	35	72°C, 5:00
HM 62	95°C, 2:00	95°C, 0:20	56°C, 0:30	72°C, 0:30	35	72°C, 5:00
HM 63	95°C, 2:00	95°C, 0:20	56°C, 0:30	72°C, 0:30	35	72°C, 5:00
HM 64	95°C, 2:00	95°C, 0:20	56°C, 0:30	72°C, 0:30	35	72°C, 5:00
HM 65	95°C, 2:00	95°C, 0:20	56°C, 0:30	72°C, 0:30	35	72°C, 5:00
HM 66	95°C, 2:00	95°C, 0:20	56°C, 0:30	72°C, 0:30	35	72°C, 5:00
HM 67	95°C, 2:00	95°C, 0:20	56°C, 0:30	72°C, 0:30	35	72°C, 5:00

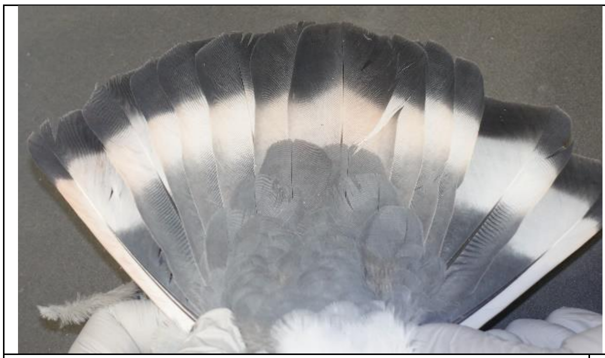	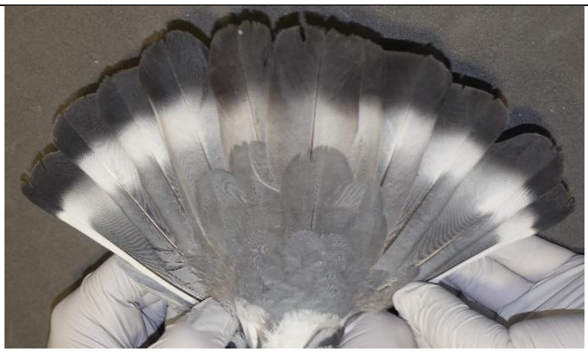
Sample ID: 05552 (male), Origin: Gurye	Sample ID: 05554 (female), Origin: Gurye
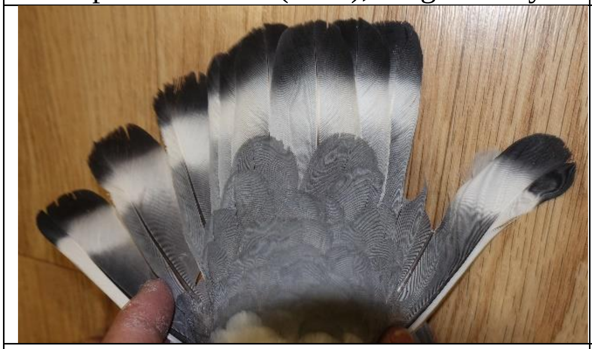	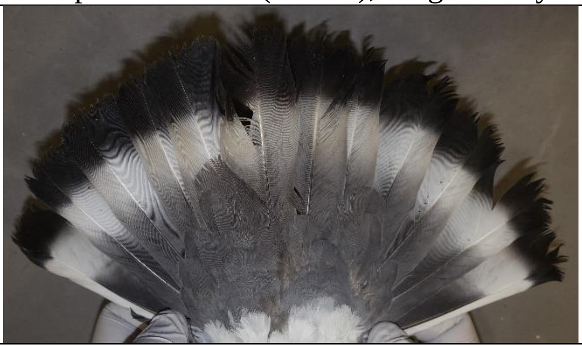
Sample ID: 05555 (female), Origin: Gurye	Sample ID: 05562 (female), Origin: Gurye
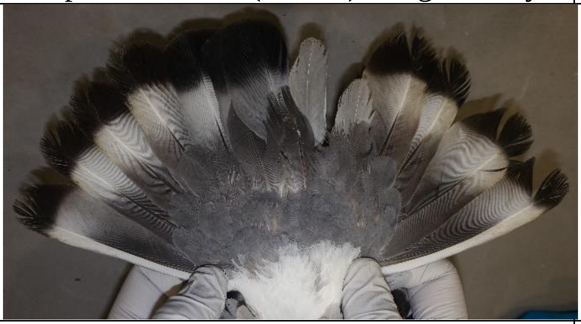	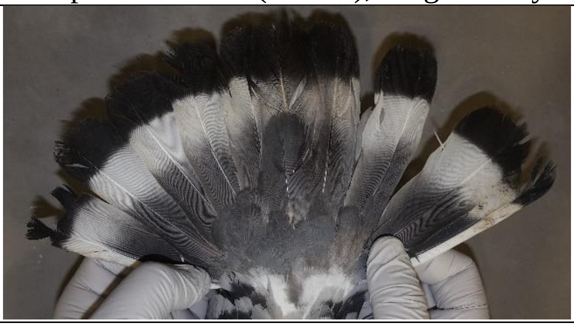
Sample ID: 05563 (female), Origin: Gurye	Sample ID: 05564 (male), Origin: Gurye
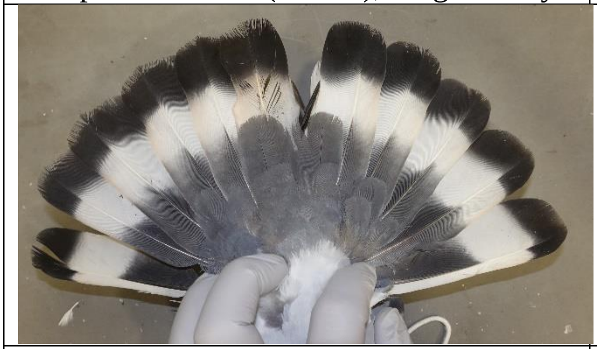	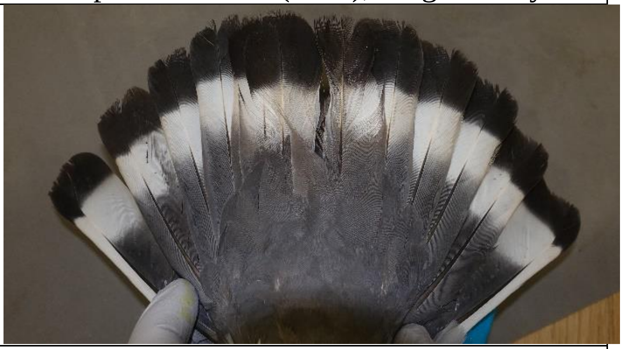
Sample ID: 05567 (male), Origin: Gurye	Sample ID: 05565 (female), Origin: Gurye

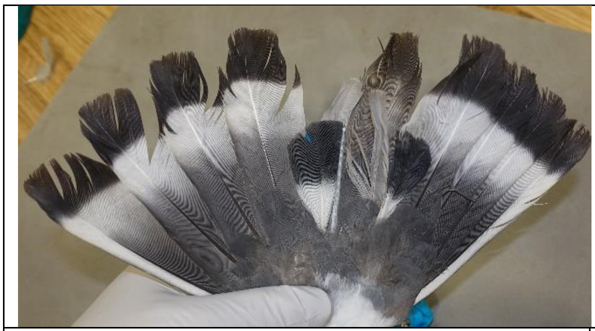	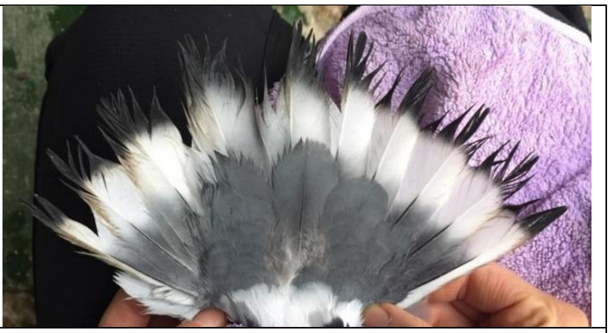
Sample ID: 05568 (female), Origin: Gurye	Sample ID: 151 (male), Origin: Goheung
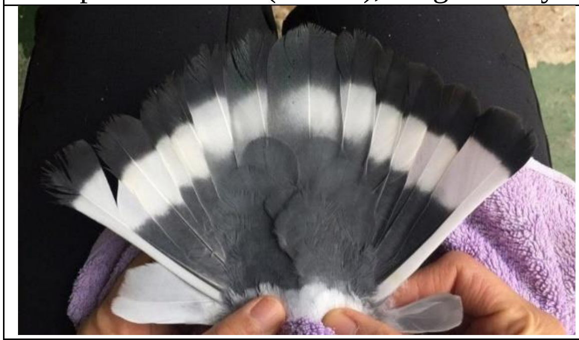	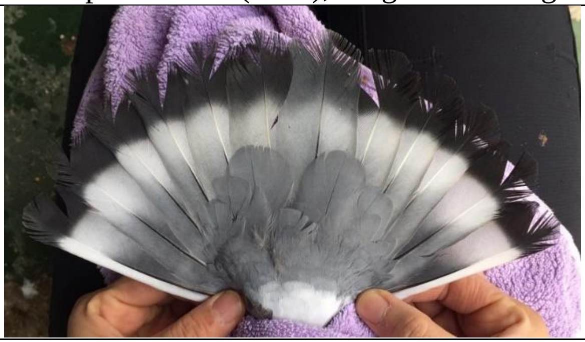
Sample ID: 152 (male), Origin: Goheung	Sample ID: 153 (male), Origin: Goheung
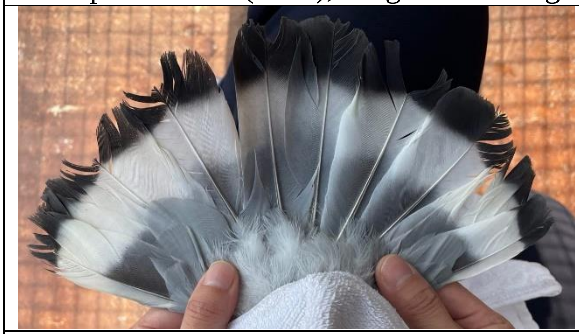	
Sample ID: 154 (male), Origin: Goheung	Sample ID: 155 (female), Origin: Goheung
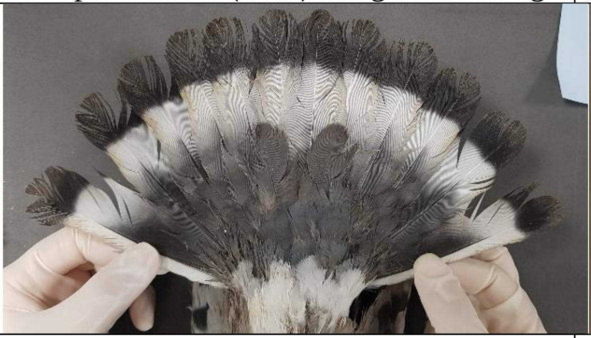	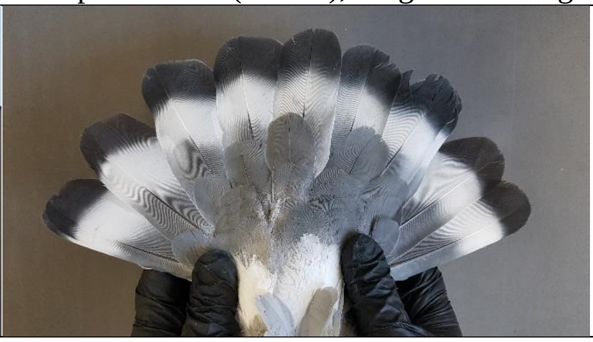
Sample ID: 02412(female), Origin: Gurye	Sample ID: 02415(male), Origin: Gurye

Supplementary Figure S1. Tail feather phenotypes identified as hill pigeon (*Columba rupestris*) for testing the species-specific InDel region using agarose gel electrophoresis.

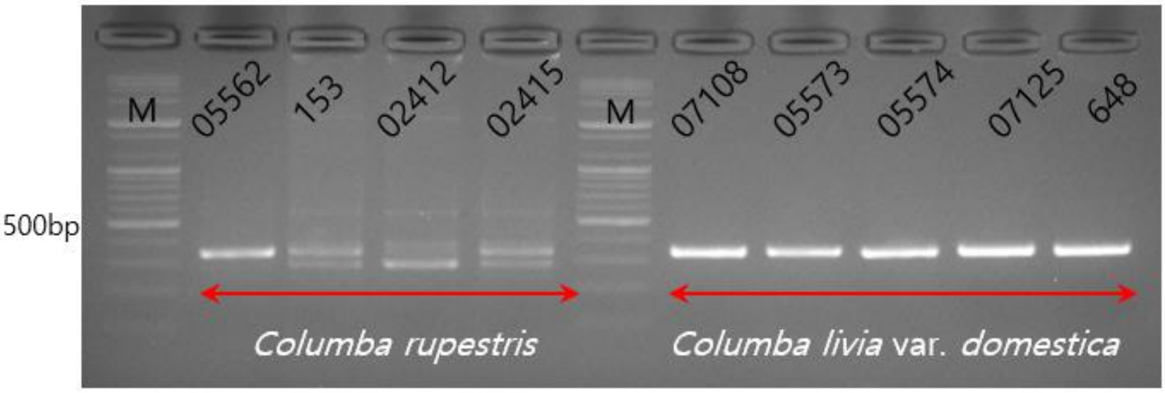

HM 1

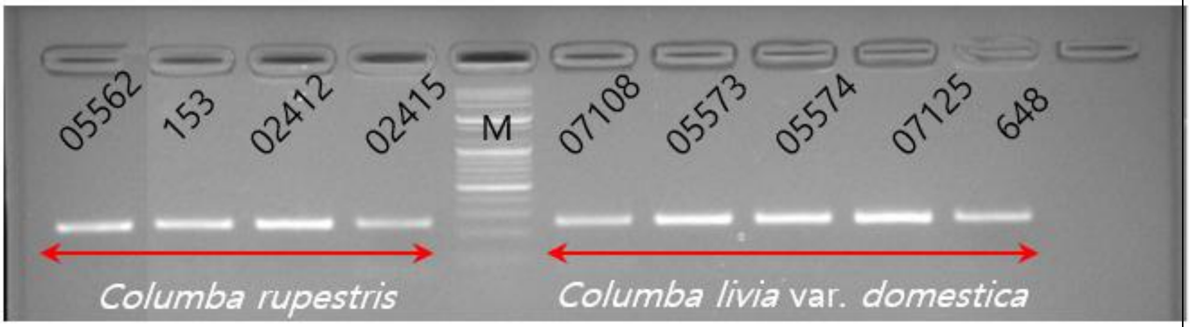

HM 2

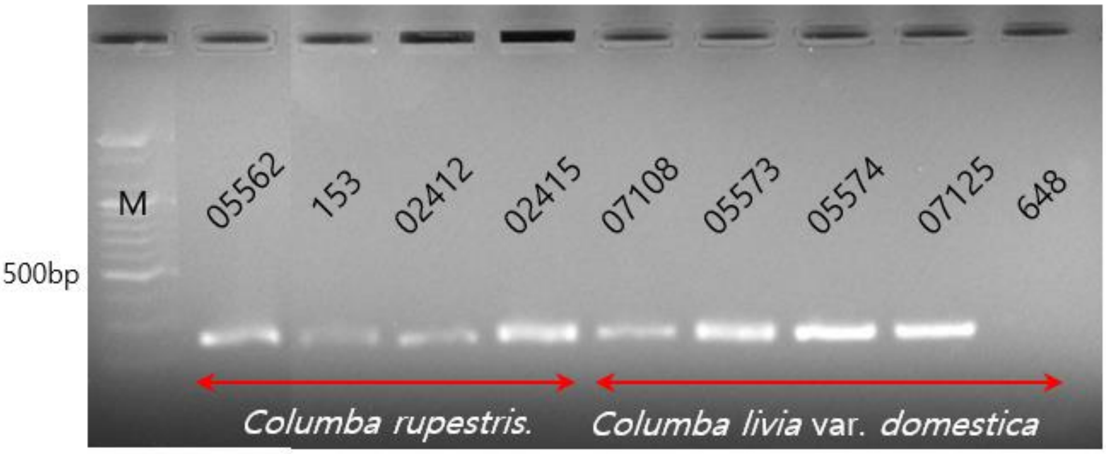

HM 3

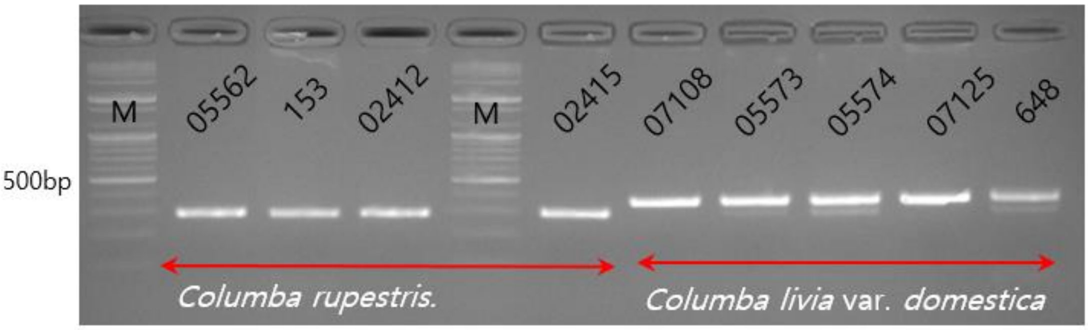

HM 4

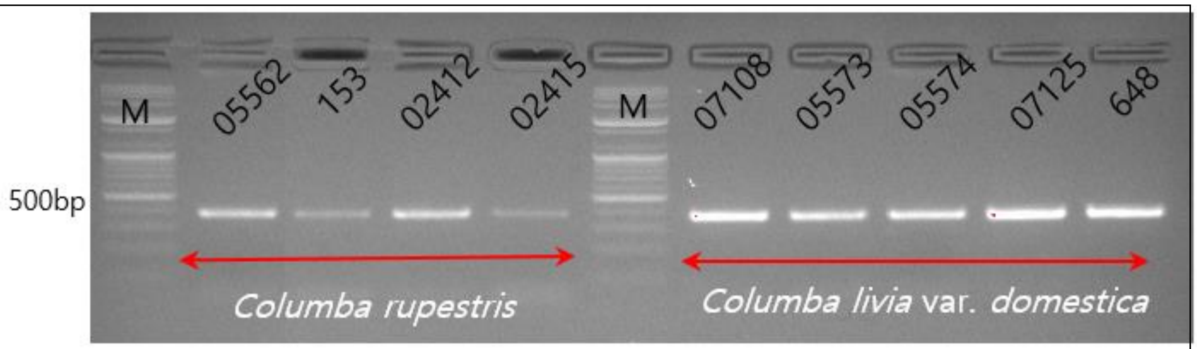

HM 5

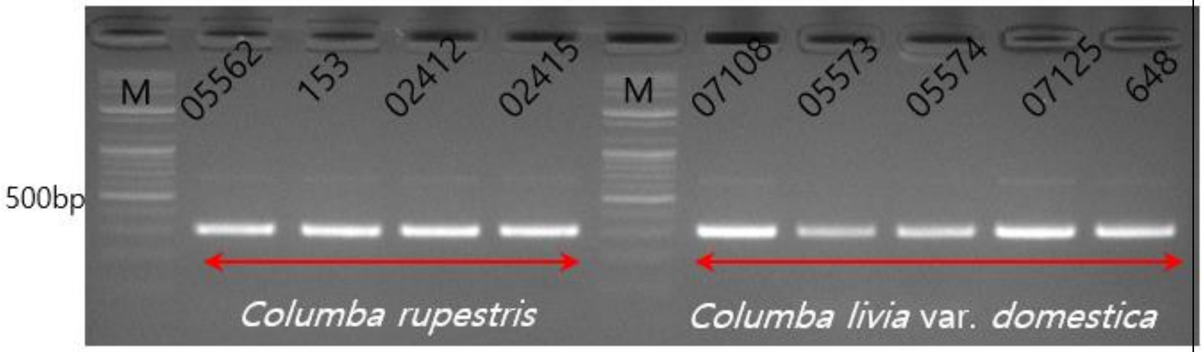

HM 6

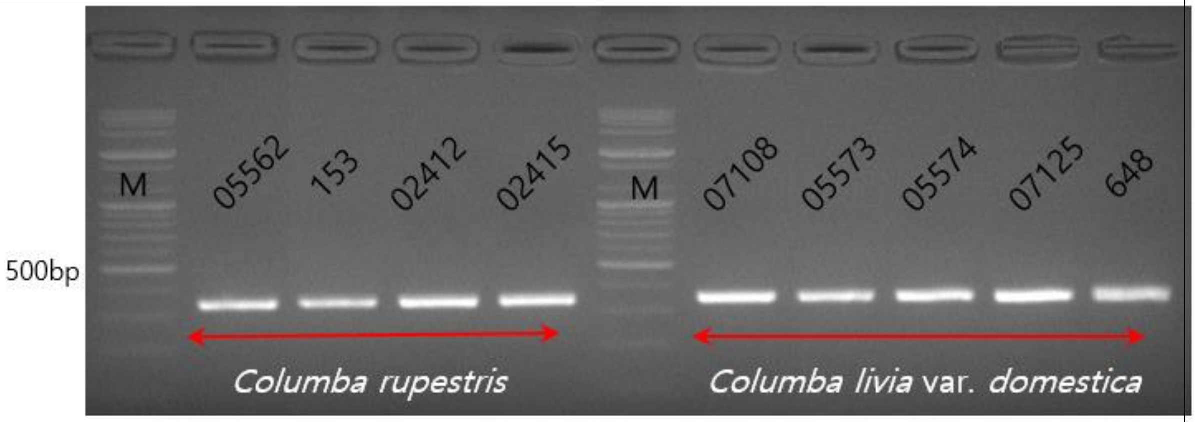

HM 7

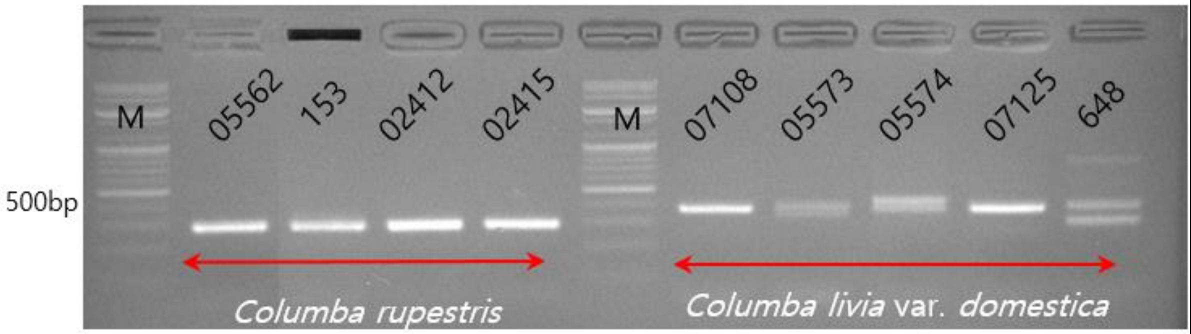

HM 8

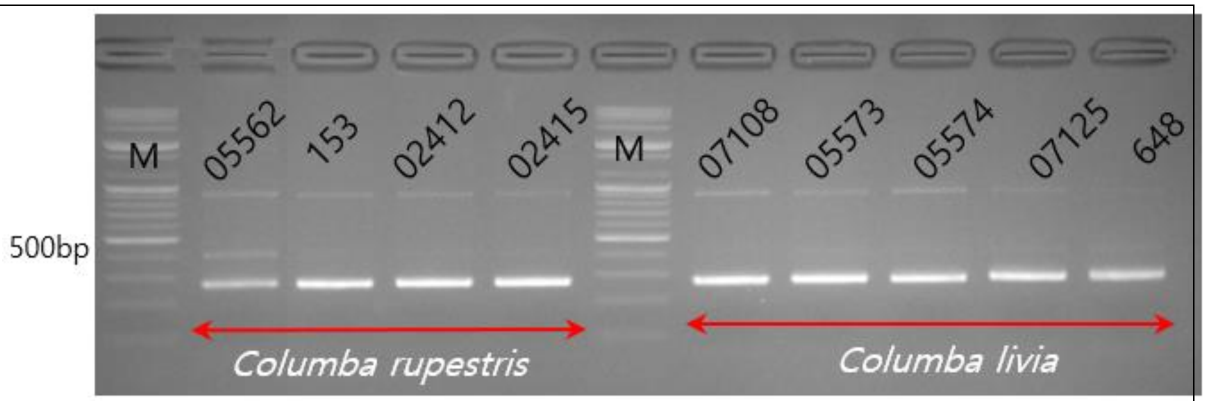

HM 9

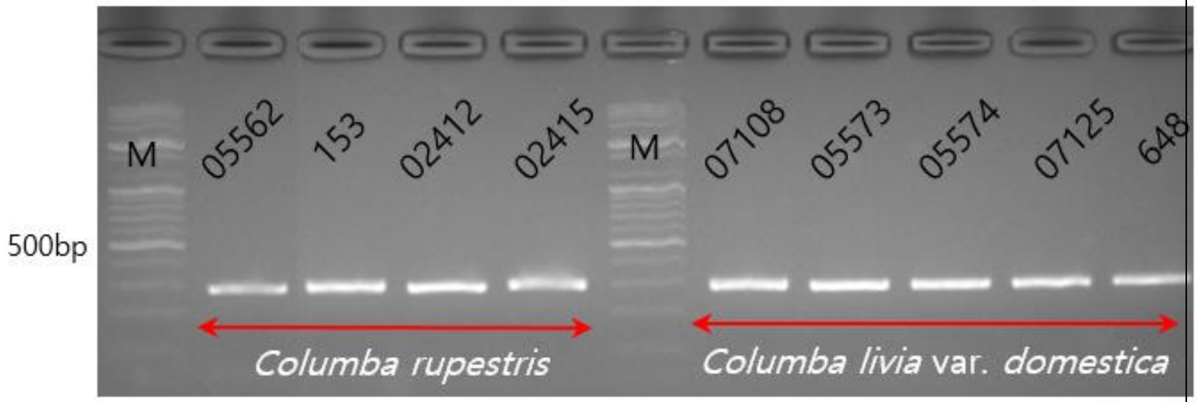

HM 10

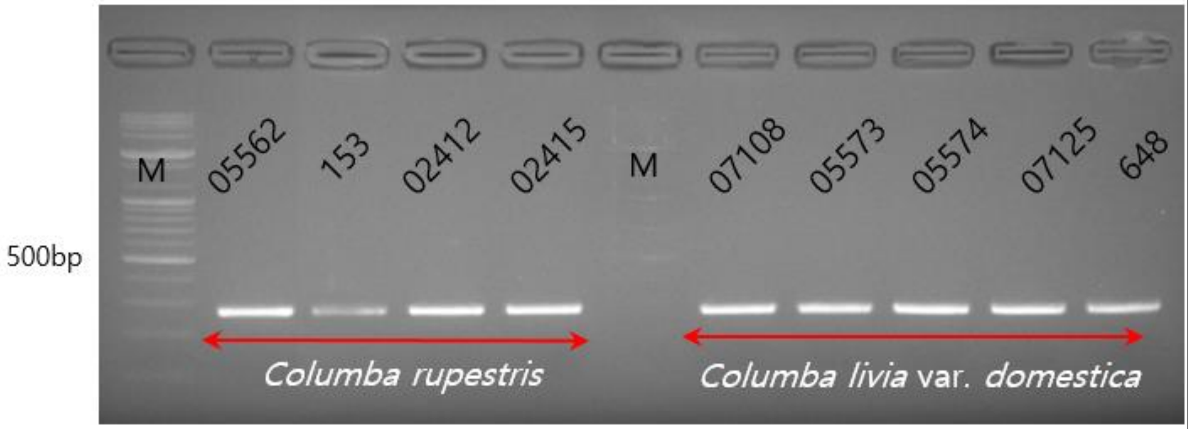

HM 11

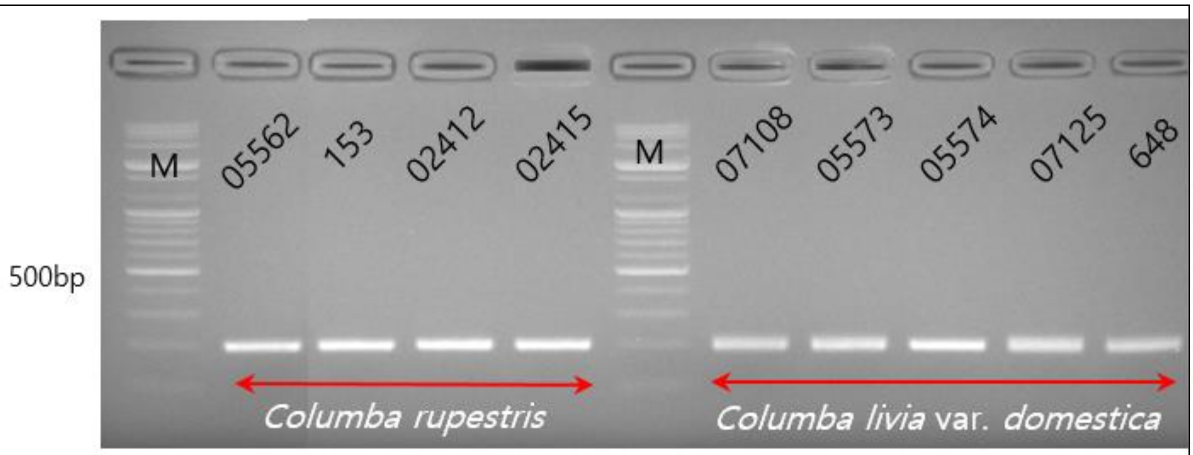

HM 12

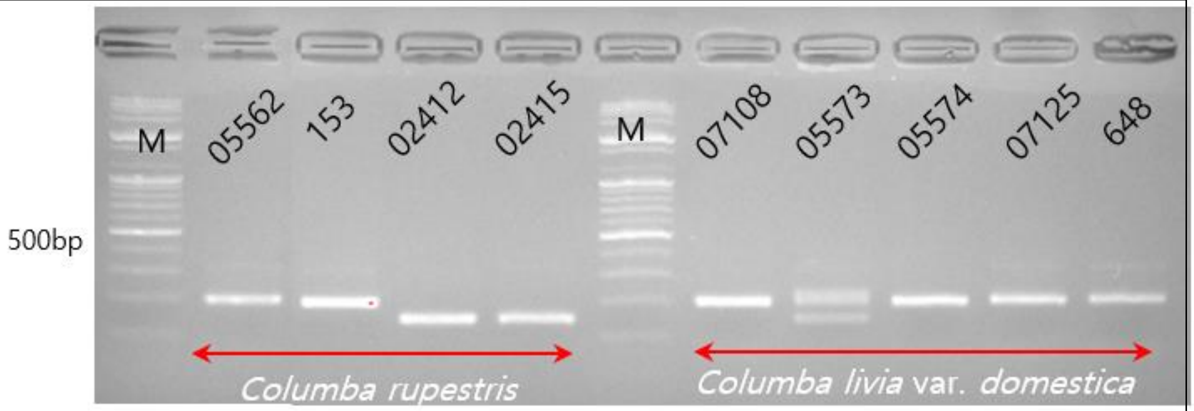

HM 13

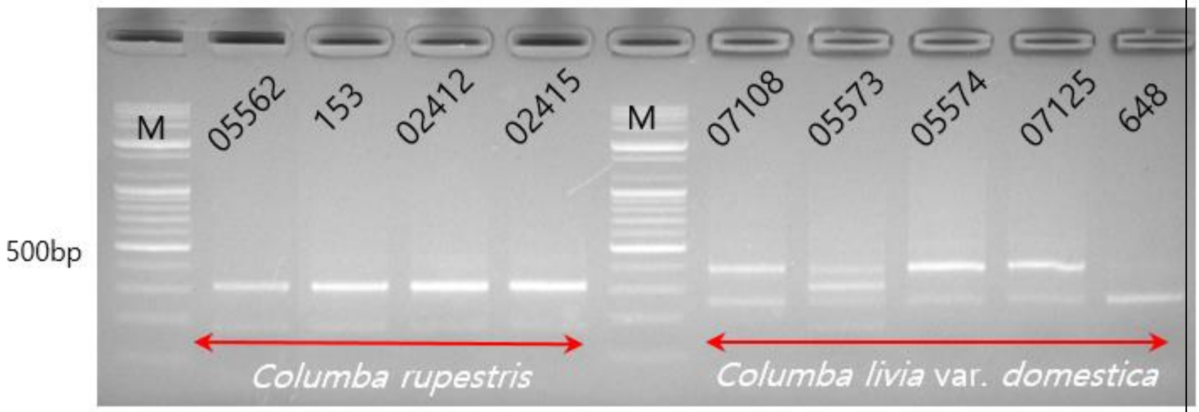

HM 14

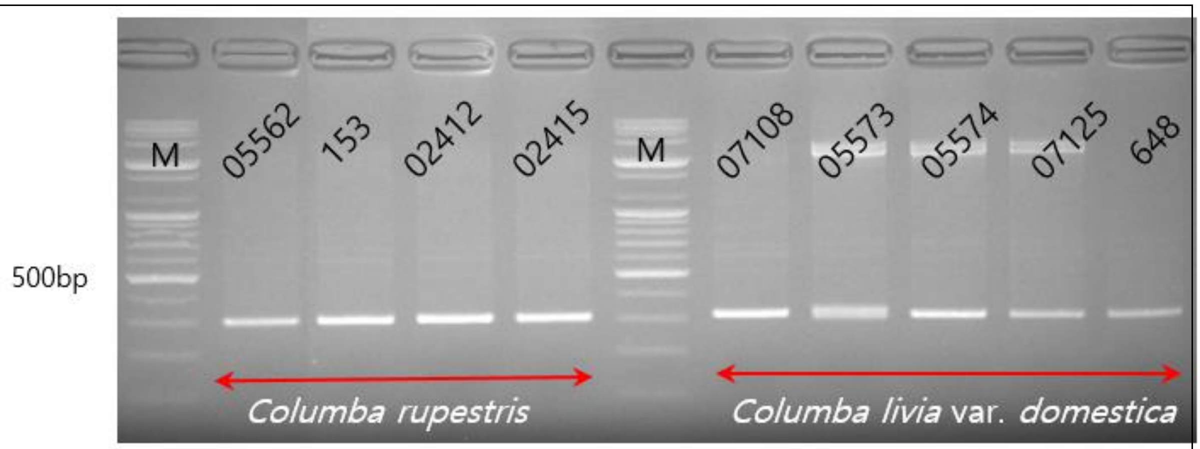

HM 15

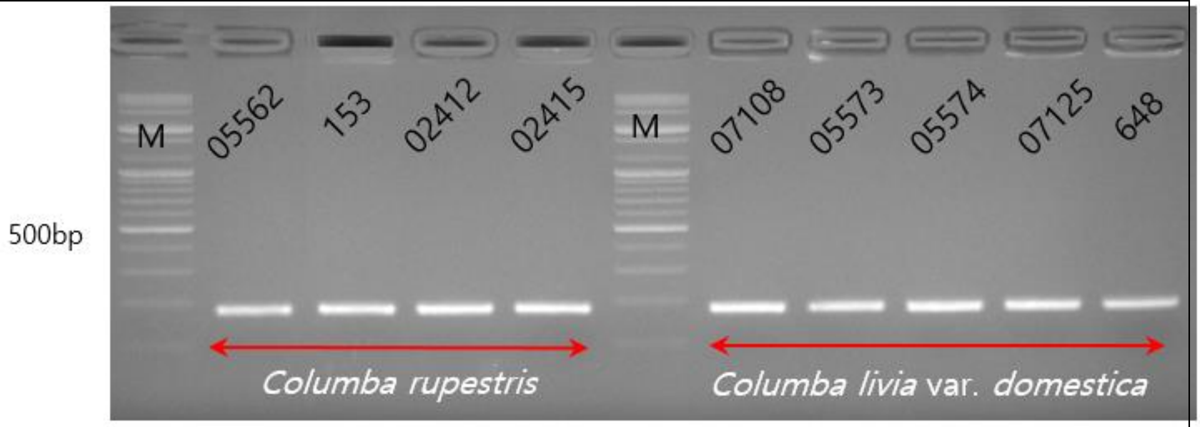

HM 16

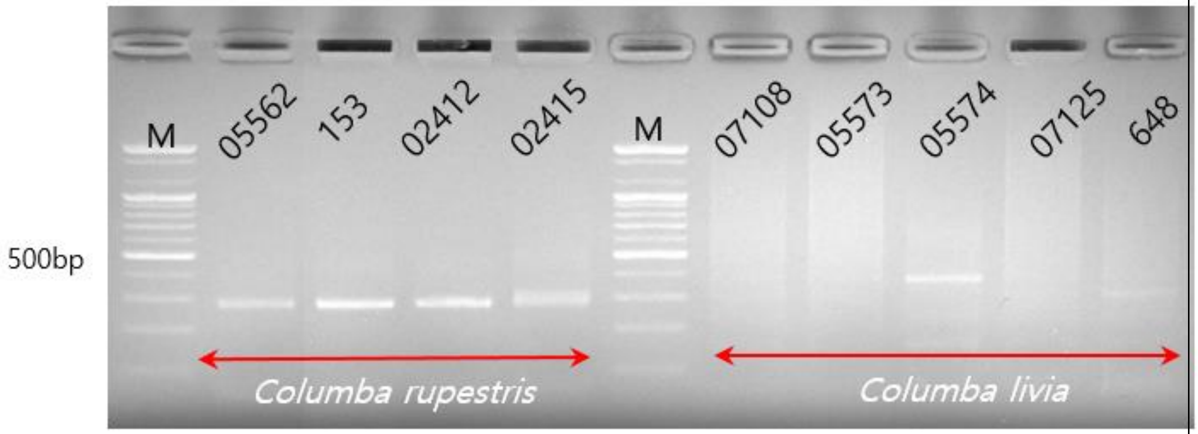

HM 17

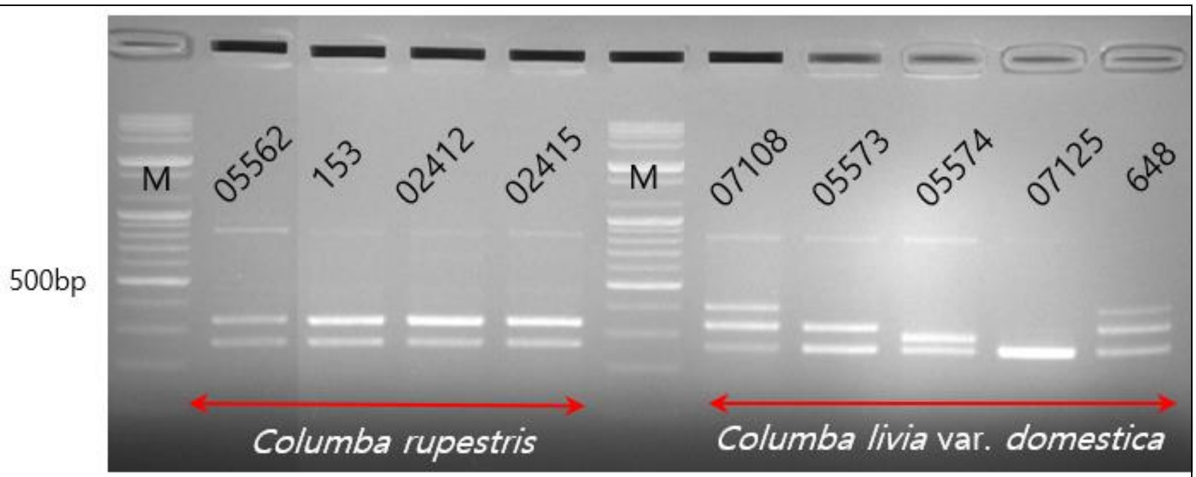

HM 18

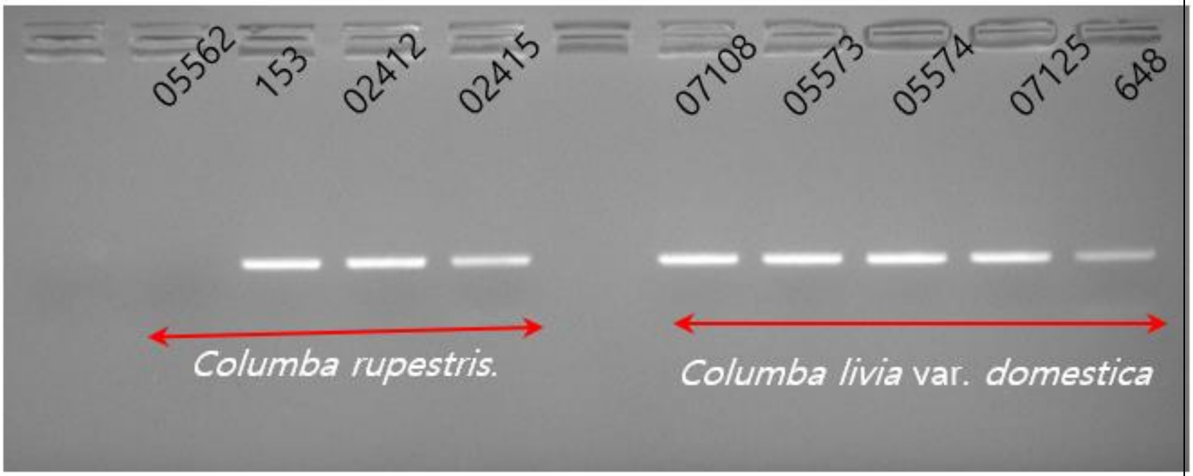

HM 19

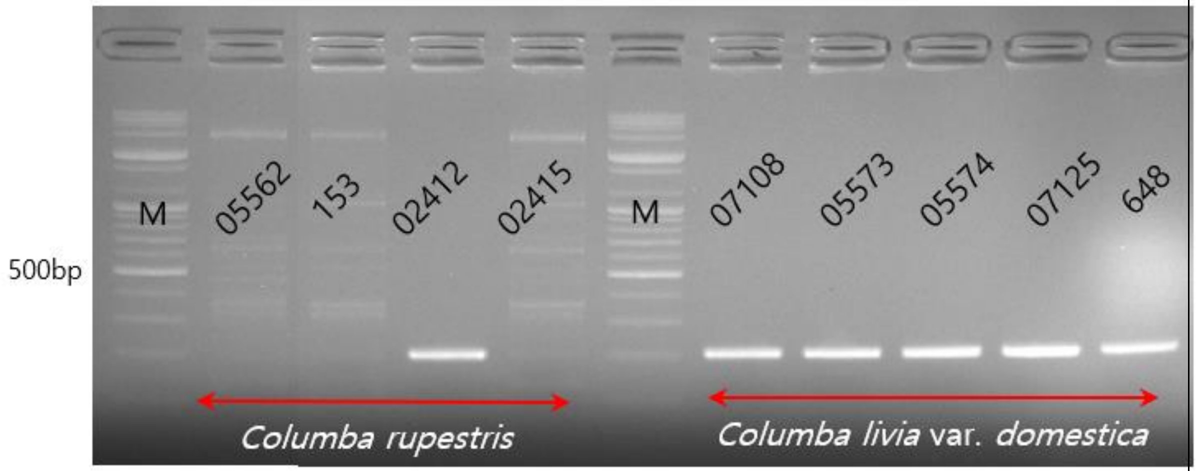

HM 20

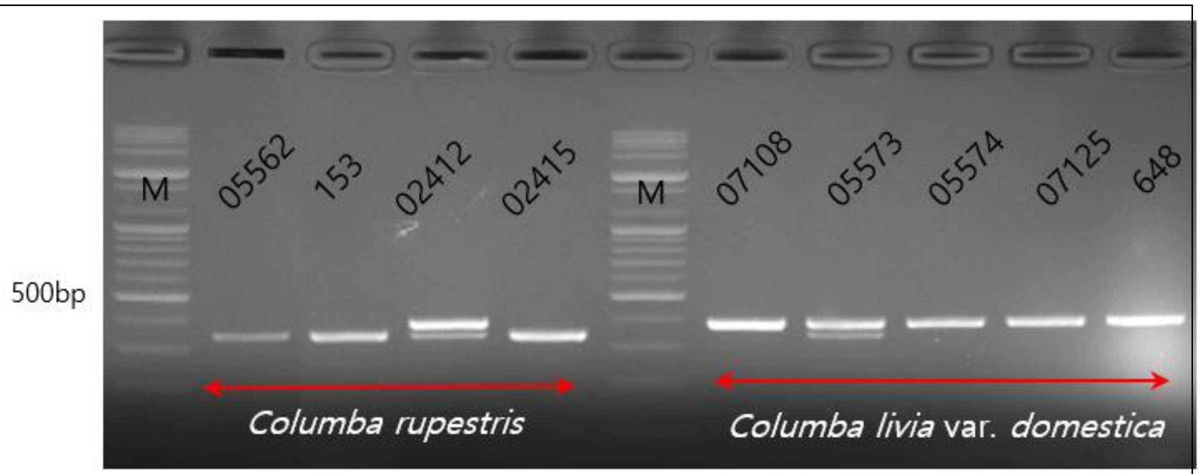

HM 21

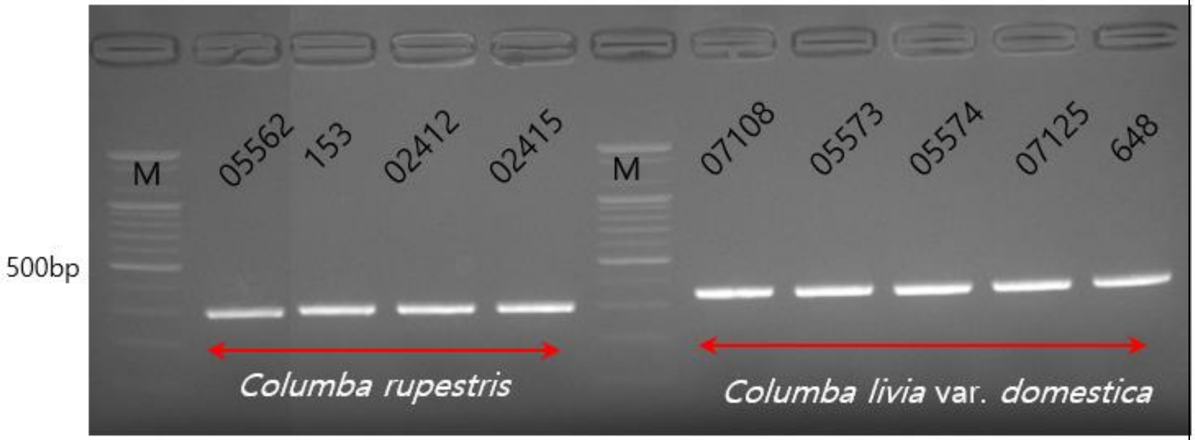

HM 22

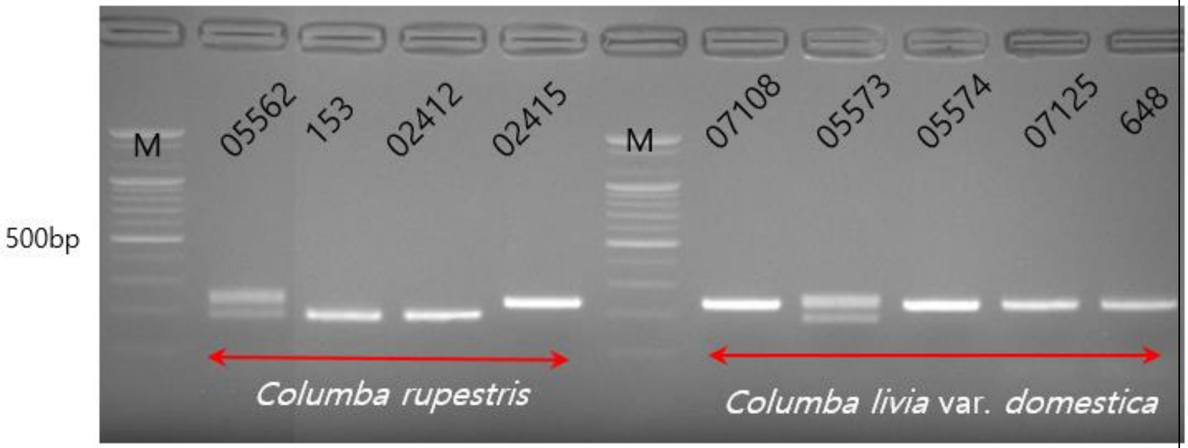

HM 23

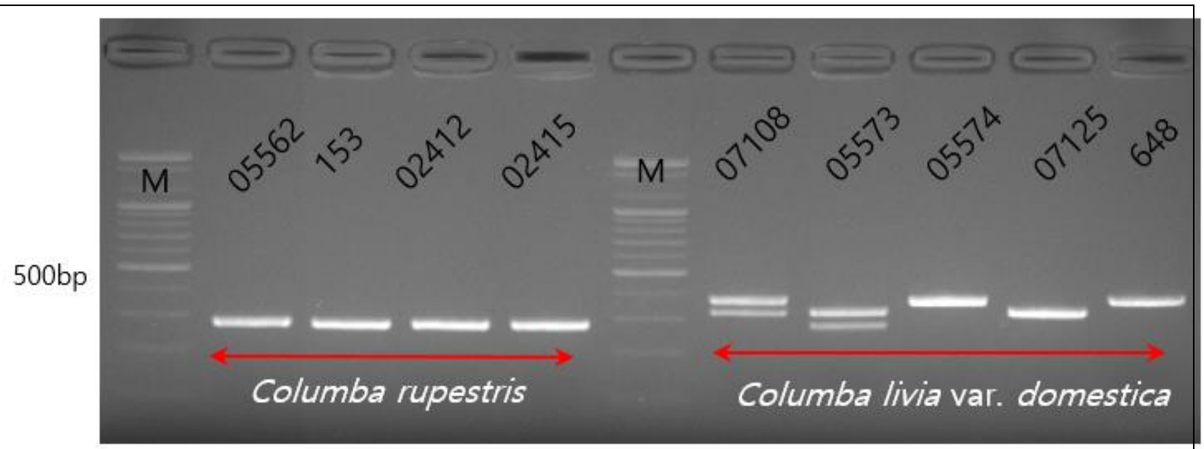

HM 24

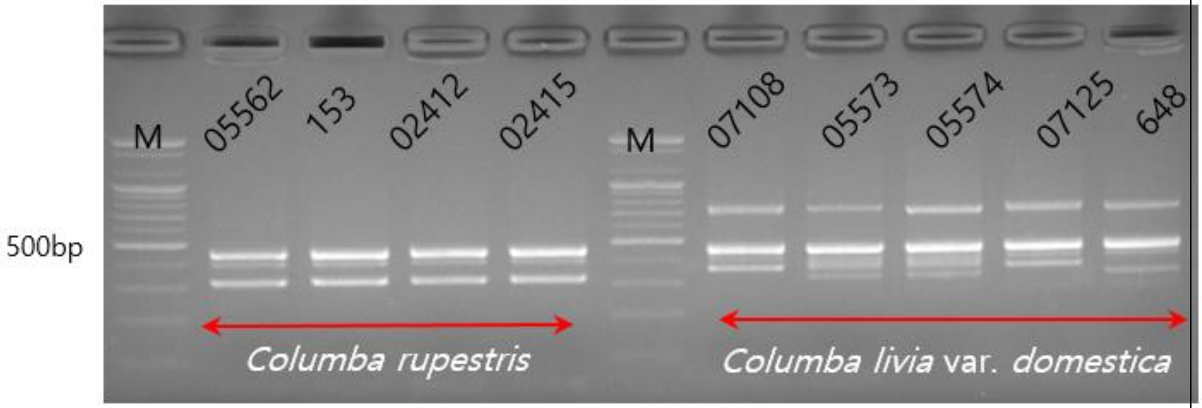

HM 25

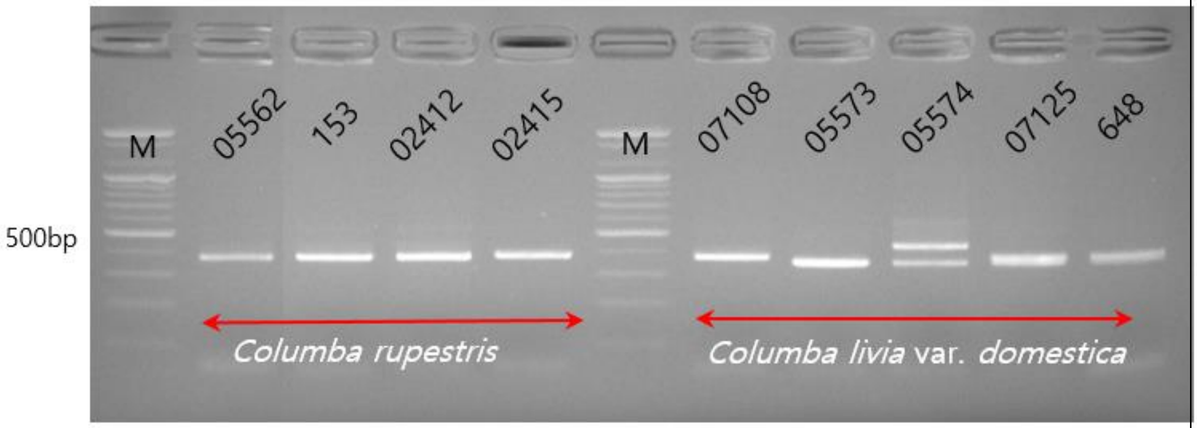

HM 26

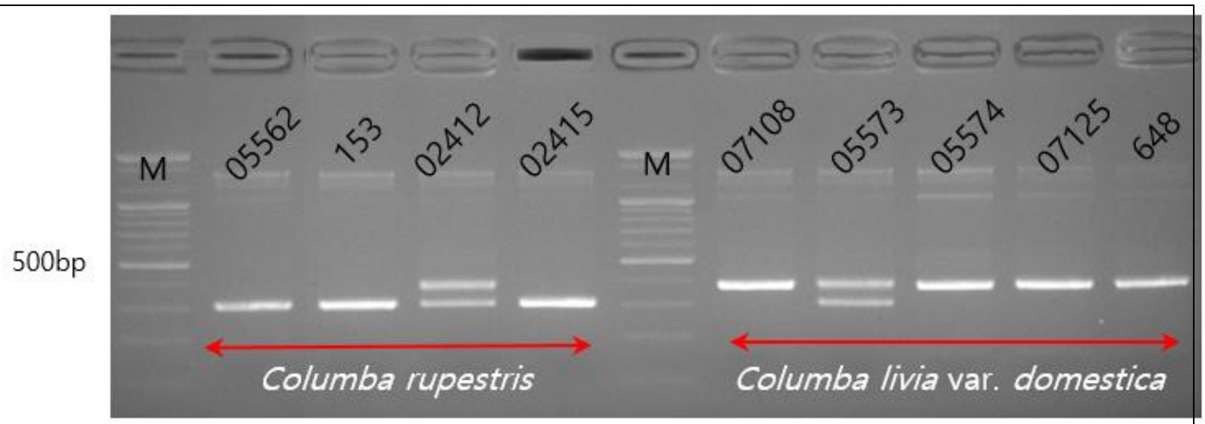

HM 27

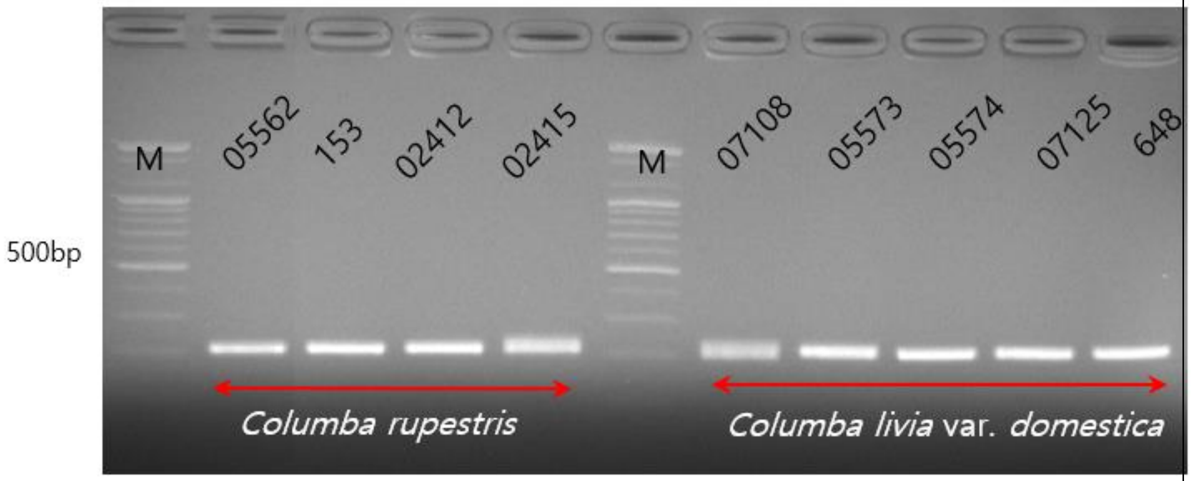

HM 28

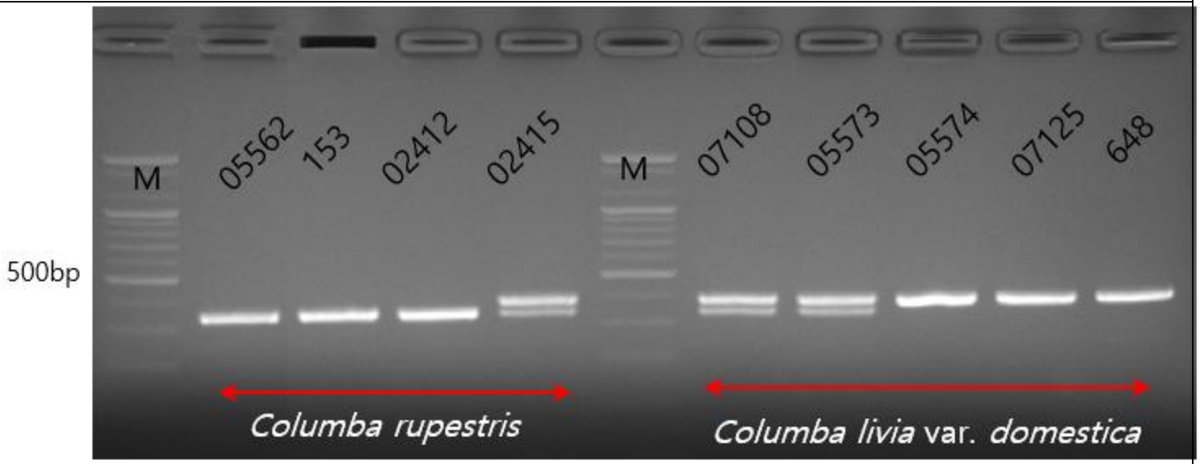

HM 29

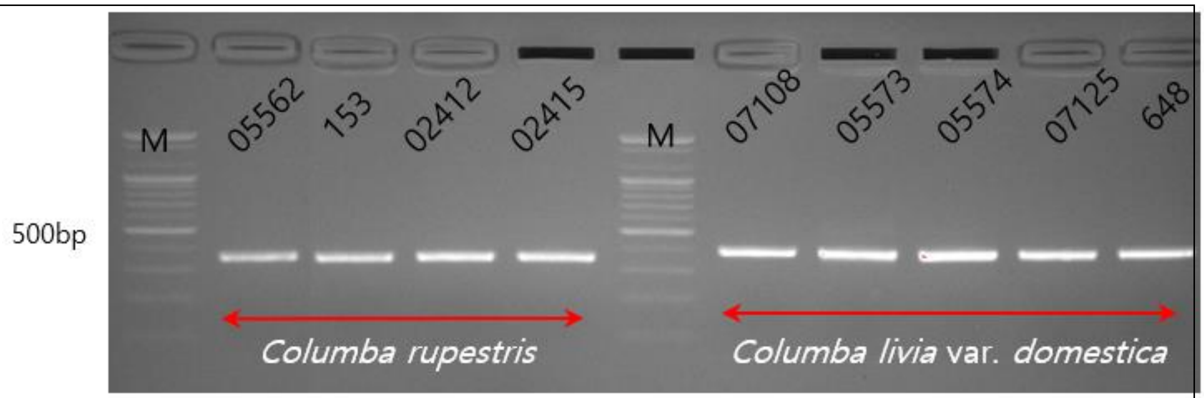

HM 30

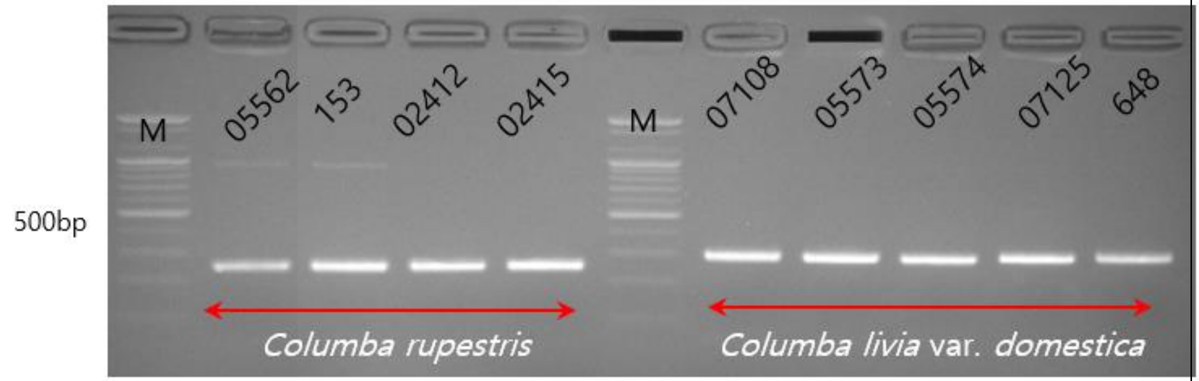

HM 31

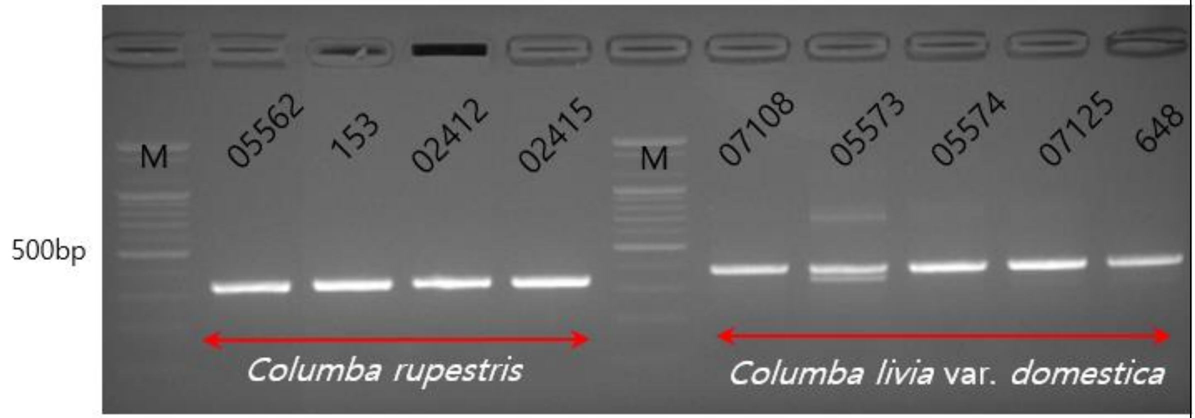

HM 32

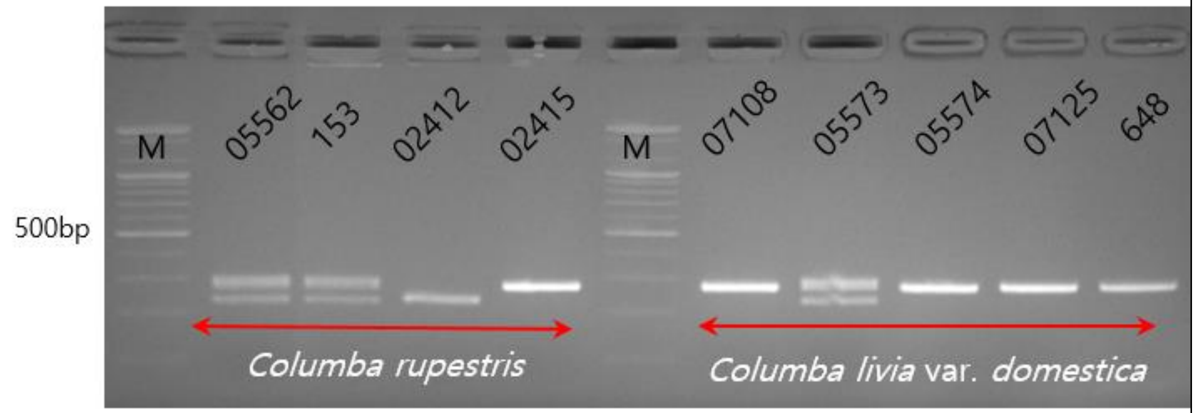

HM 33

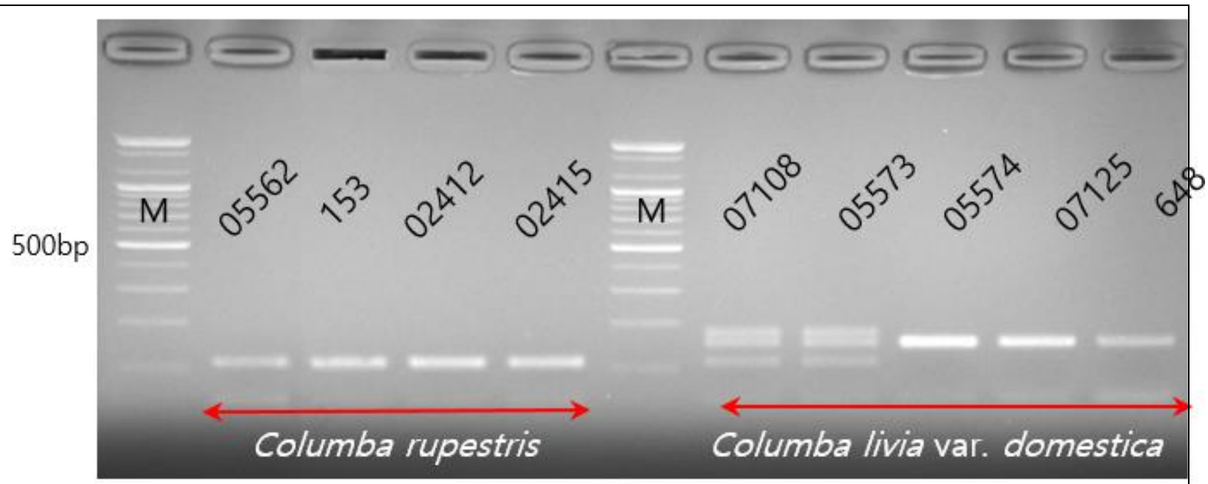

HM 34

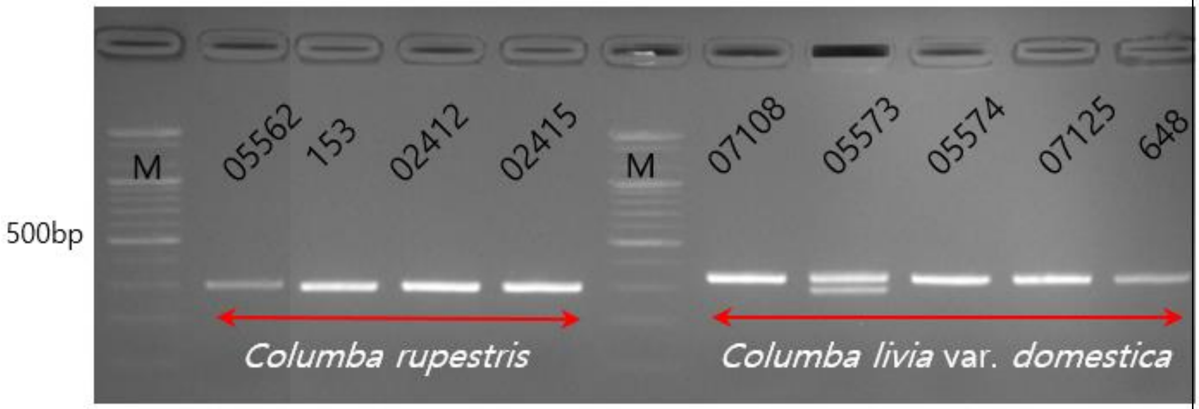

HM 35

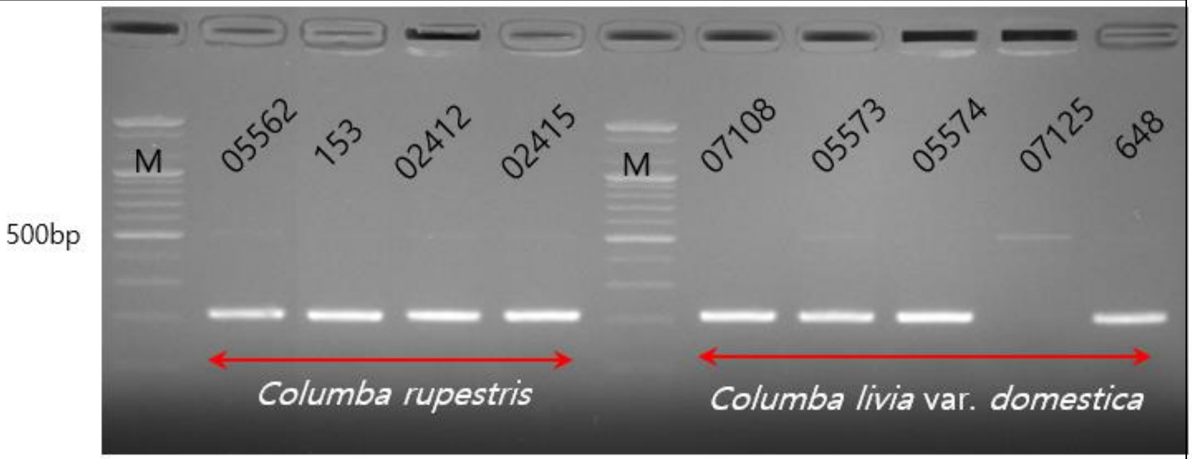

HM 36

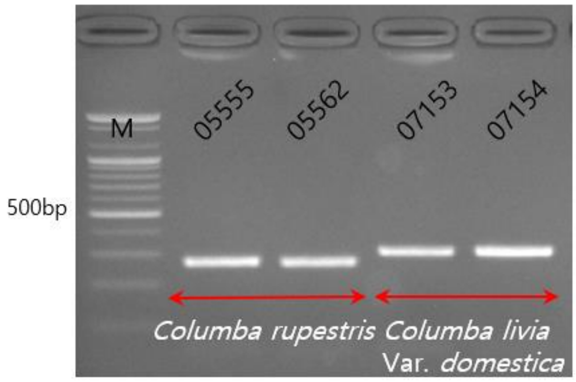

HM 37,

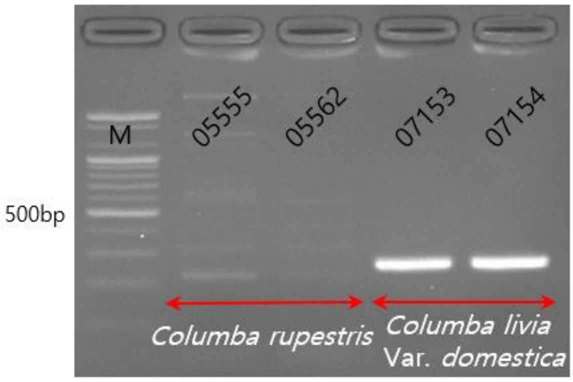

HM 38

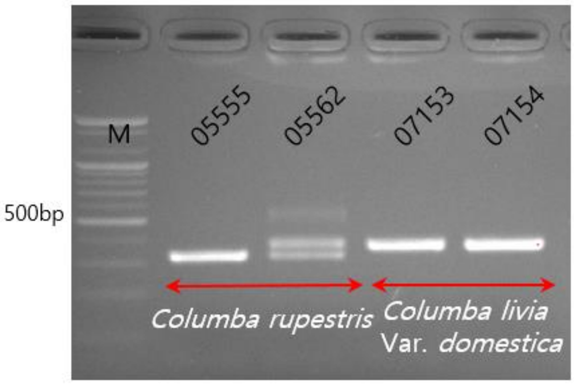

HM 39,

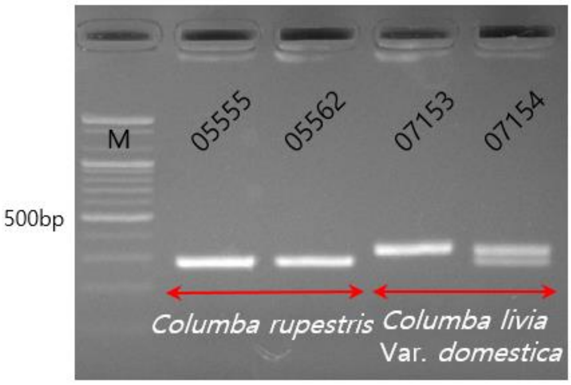

HM 40

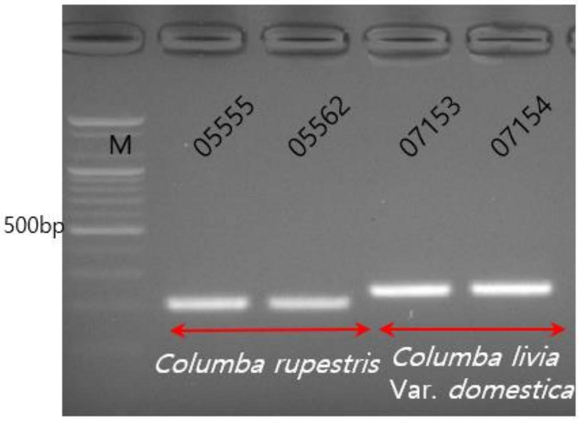

HM 41,

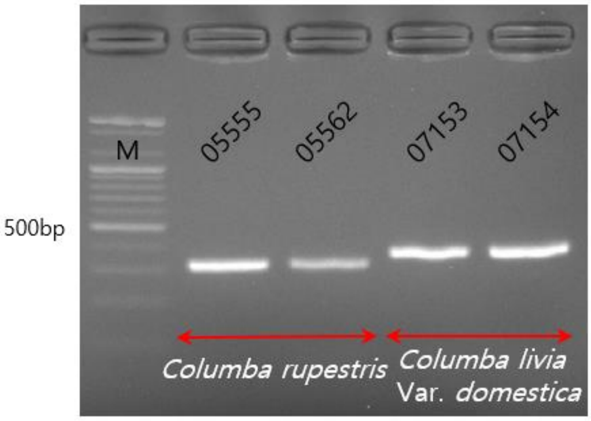

HM 42

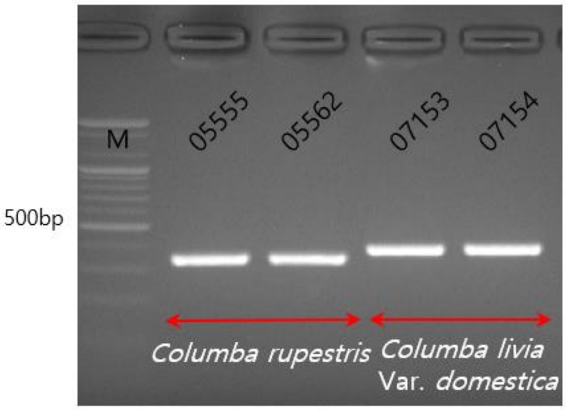

HM 43,

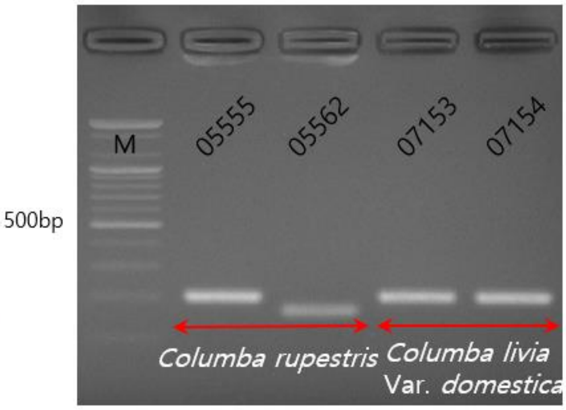

HM 44

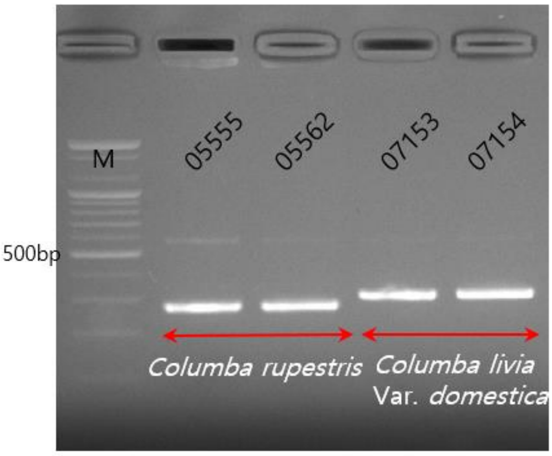

HM 45,

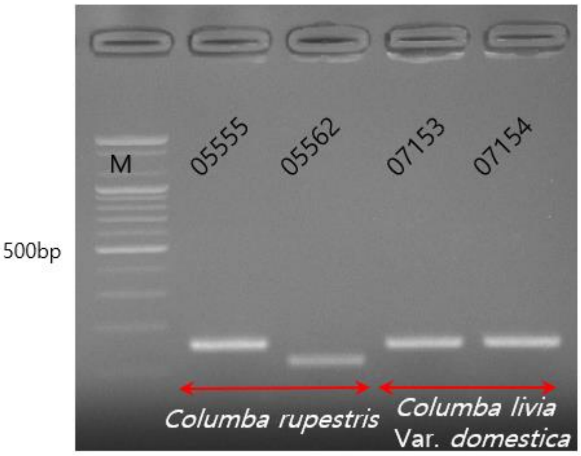

HM 46

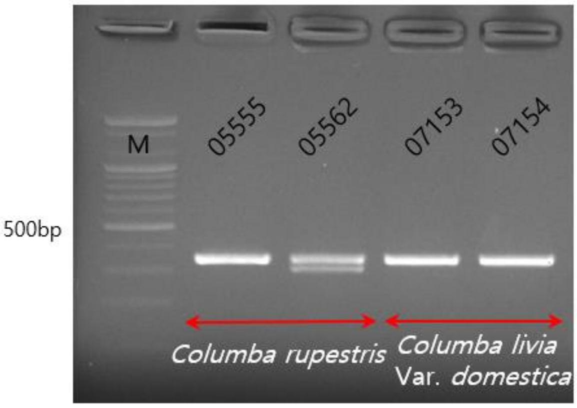

HM 47,

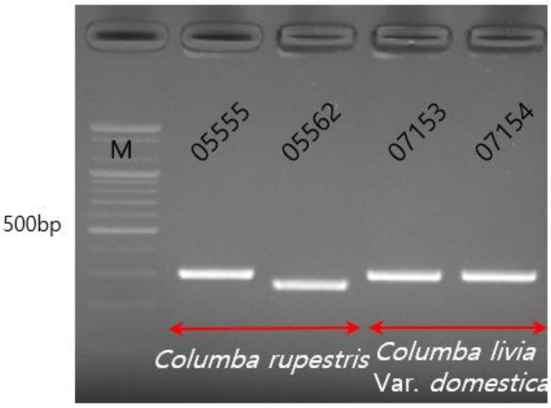

HM 48

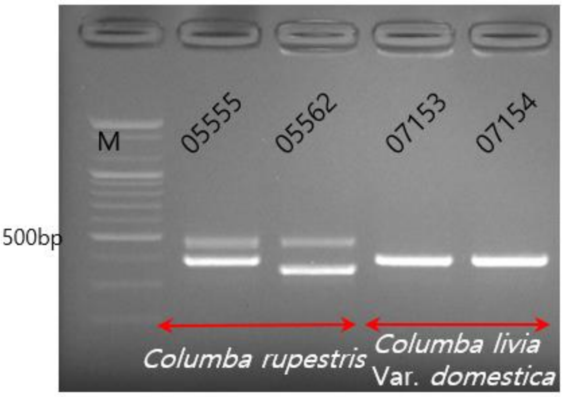

HM 49,

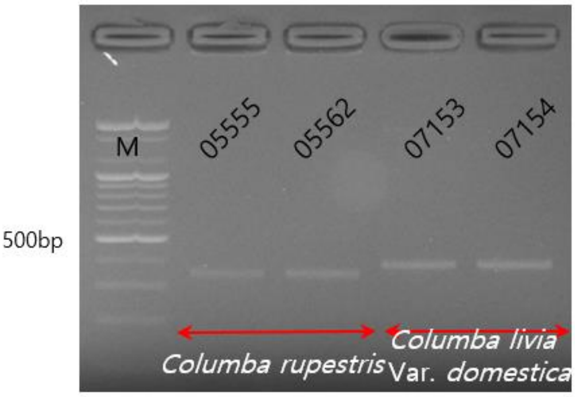

HM 50

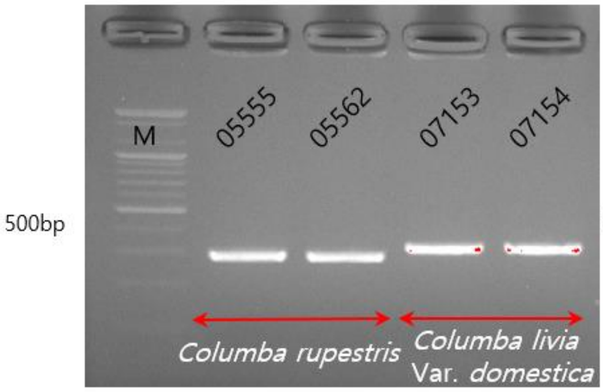

HM 51,

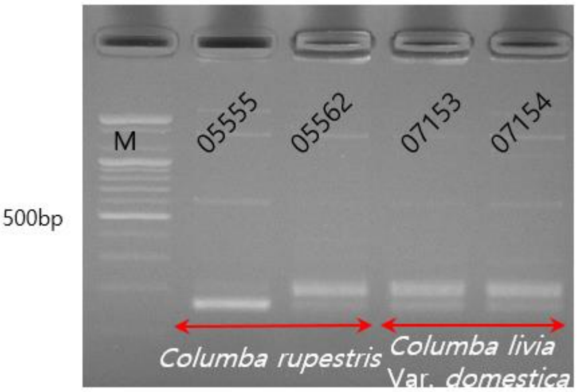

HM 52

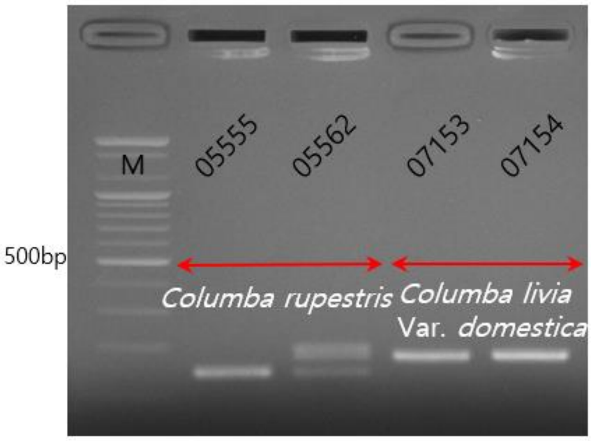

HM 53,

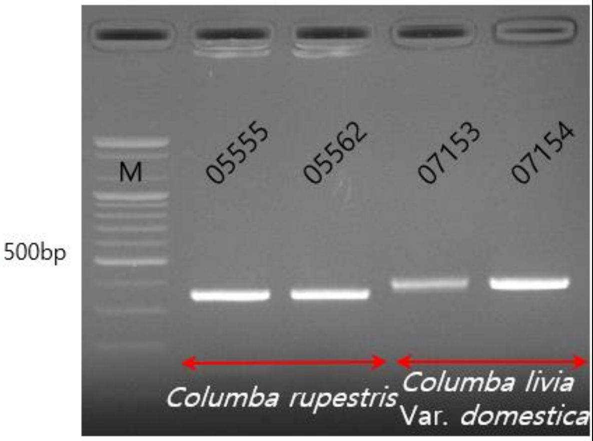

HM 54

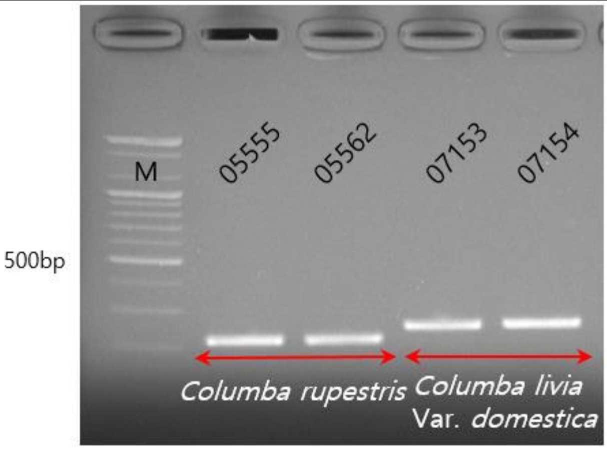

HM 55,

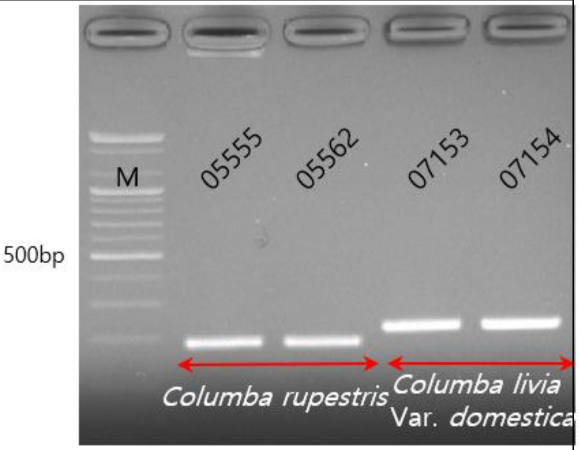

HM 56

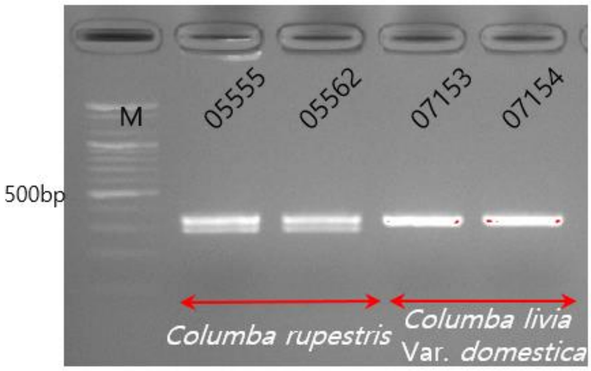

HM 57,

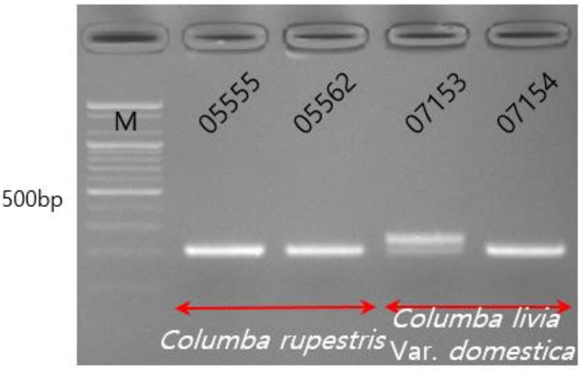

HM 58

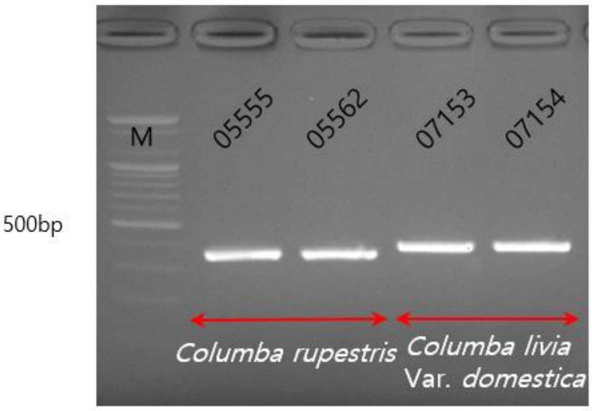

HM 59,

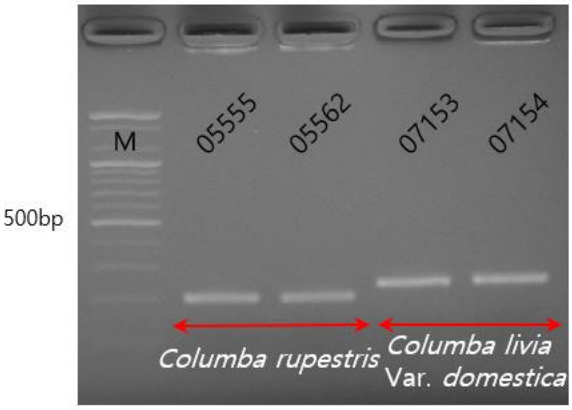

HM 60

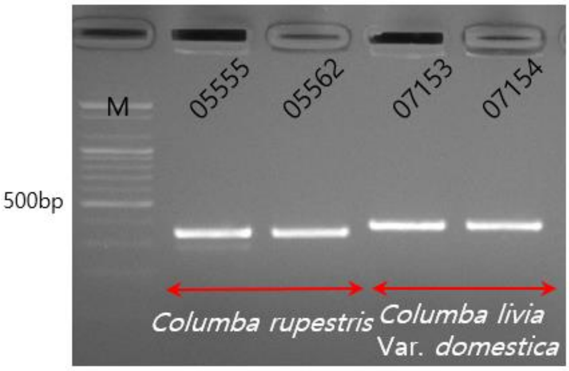

HM 61,

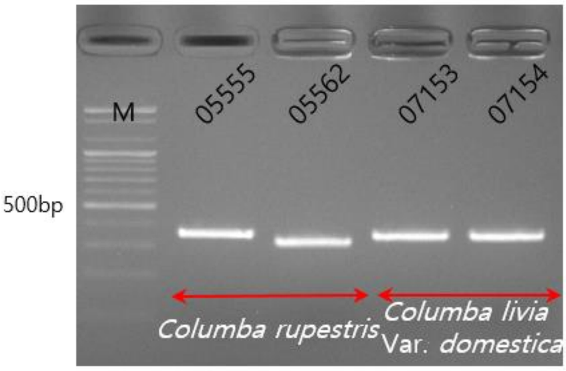

HM 62

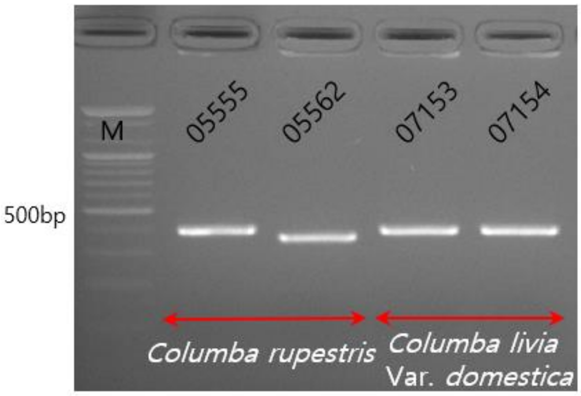

HM 63,

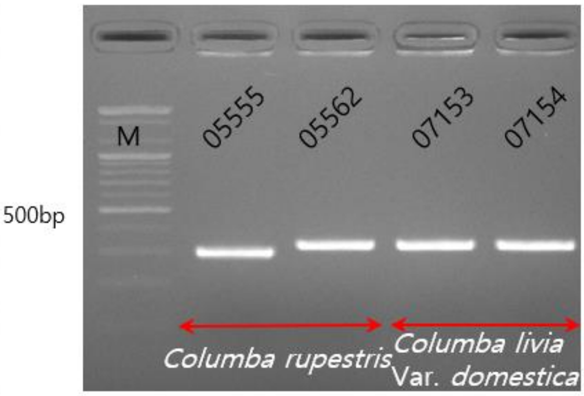

HM 64

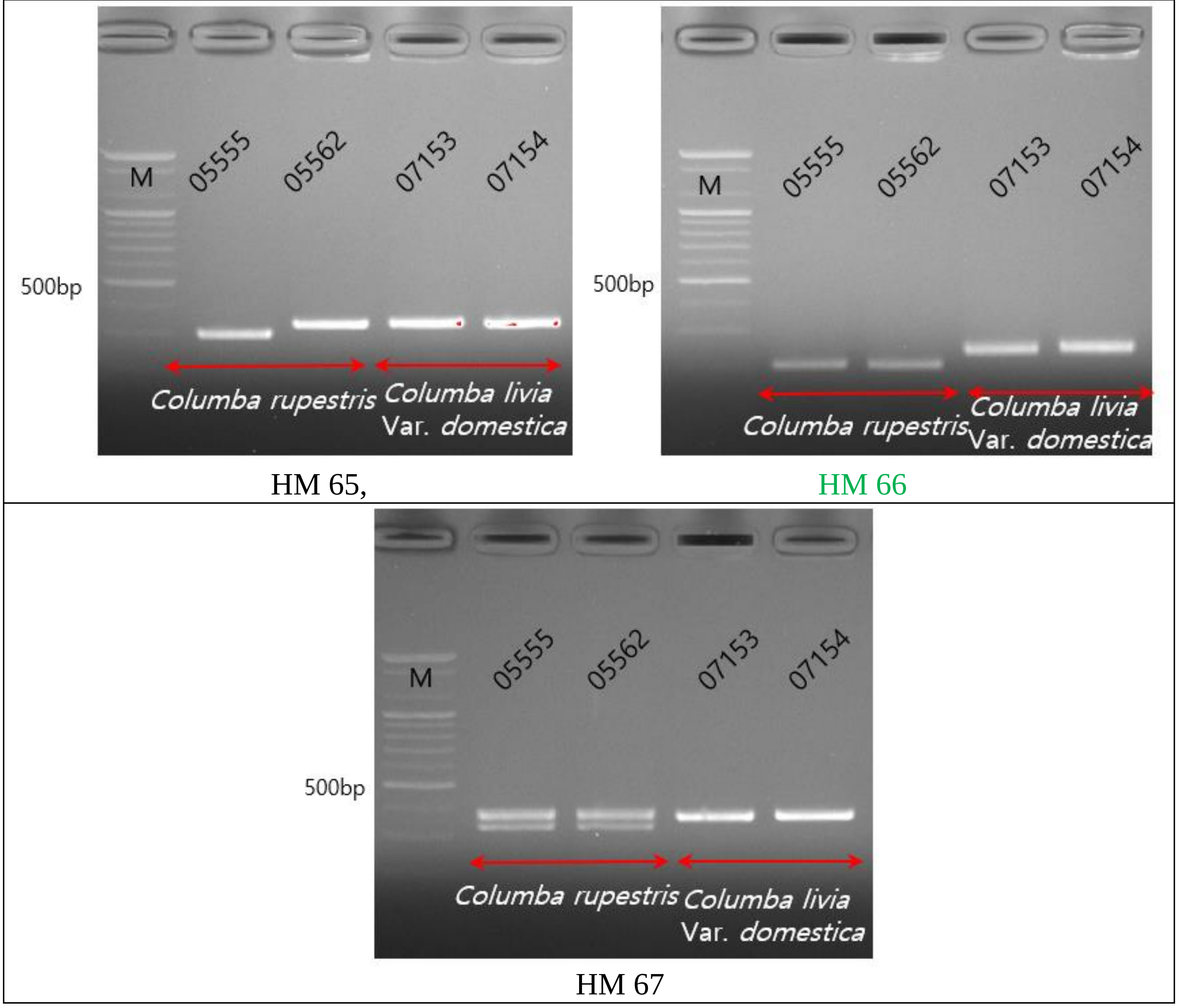

Supplementary Figure S2. Test of 67 primers to identify the species-specific InDel region of hill pigeon (*Columba rupestris*) and feral pigeon (*Columba livia var. domestica*) using agarose gel electrophoresis. Selected primers were indicated in green.

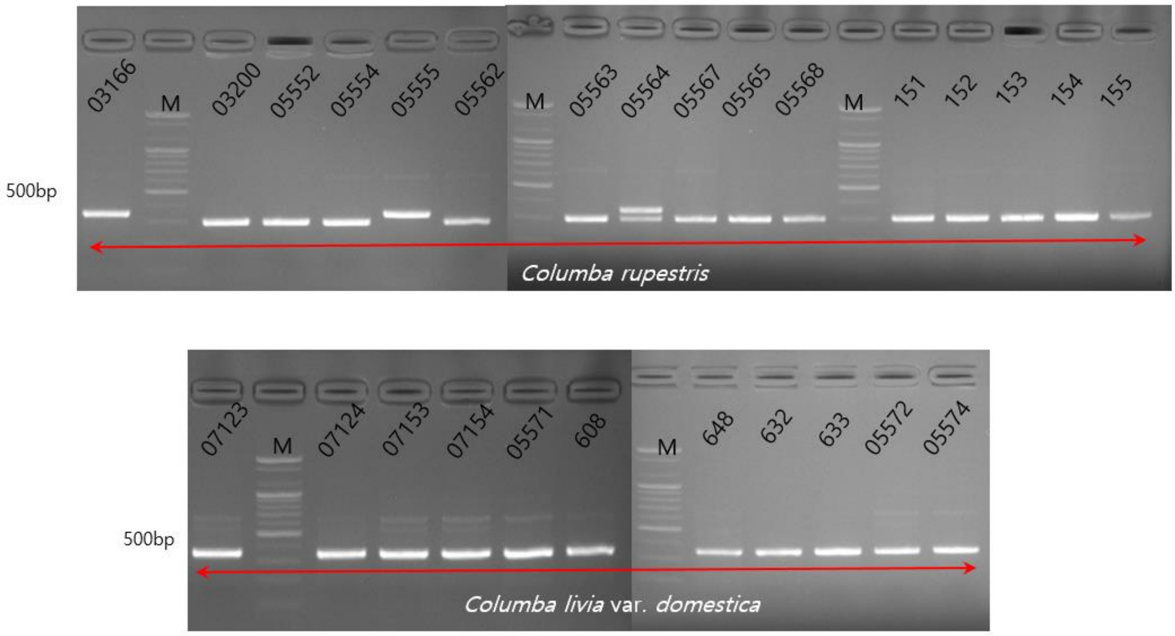
03166 M 03200 05552 05554 05555 05562 M 05563 05564 05567 05565 05568 M 151 152 153 154 155
500bp
Columba rupestris
07123 M 07124 07153 07154 05571 608 M 648 632 633 05572 05574
500bp
Columba livia var. domestica

HM 22

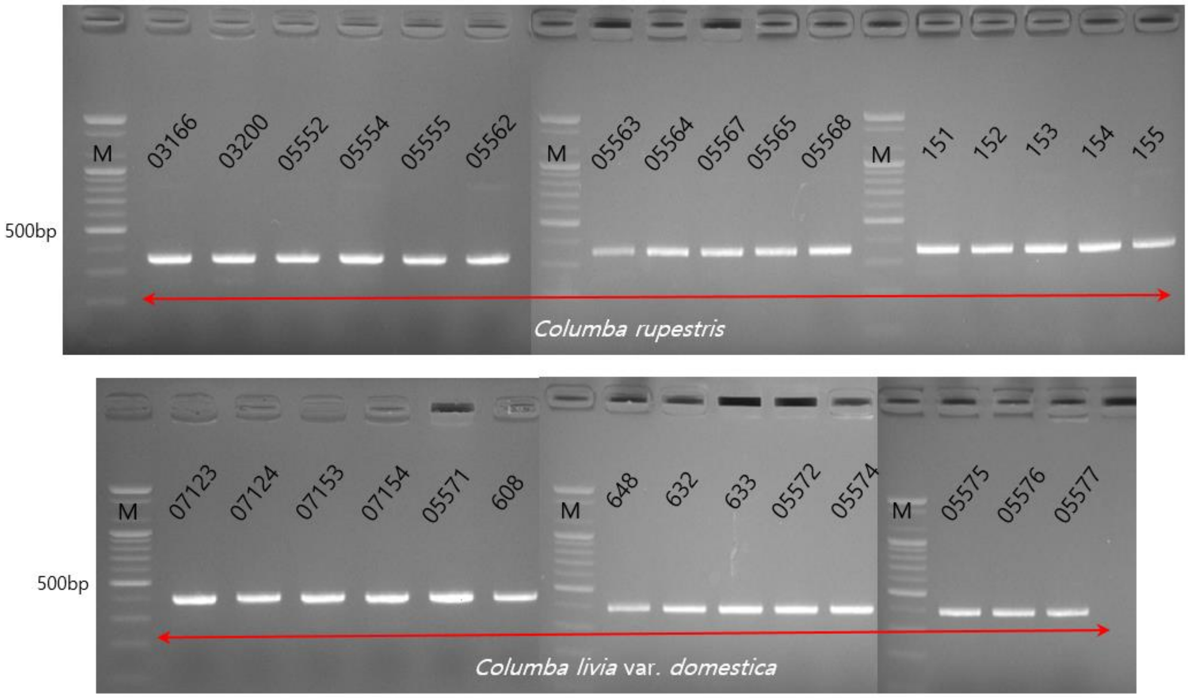
M 03166 03200 05552 05554 05555 05562 M 05563 05564 05567 05565 05568 M 151 152 153 154 155
500bp
Columba rupestris
M 07123 07124 07153 07154 05571 608 M 648 632 633 05572 05574 M 05575 05576 05577
500bp
Columba livia var. domestica

HM 30

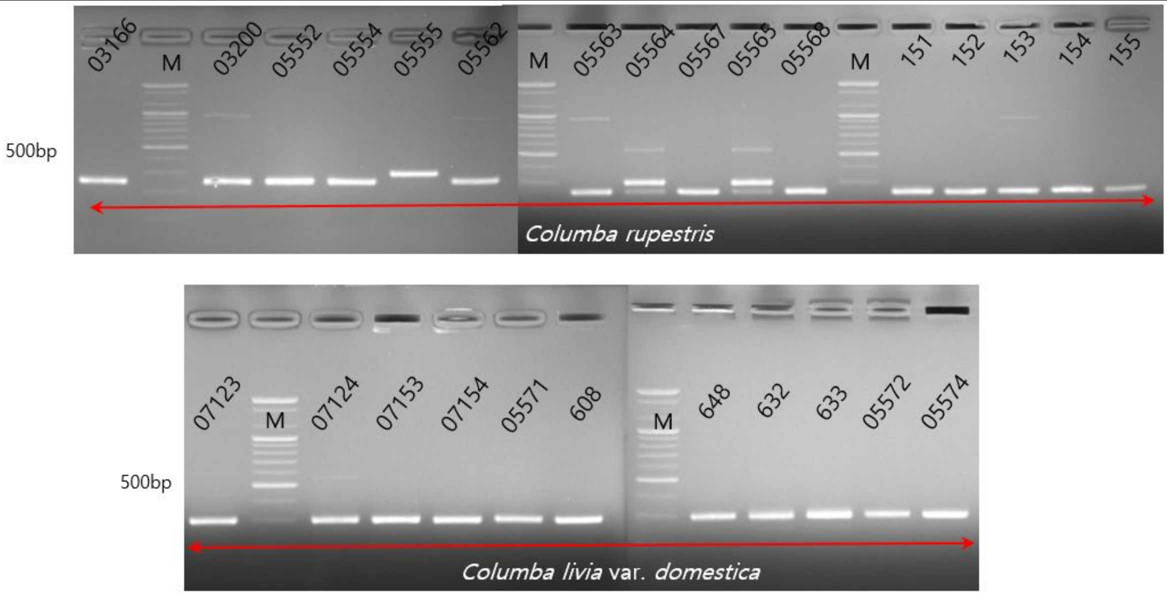

500bp
03166 M 03200 05552 05554 05555 05562 M 05563 05564 05567 05565 05568 M 151 152 153 154 155
Columba rupestris
500bp
07123 M 07124 07153 07154 05571 608 M 648 632 633 05572 05574
Columba livia var. domestica

HM 31

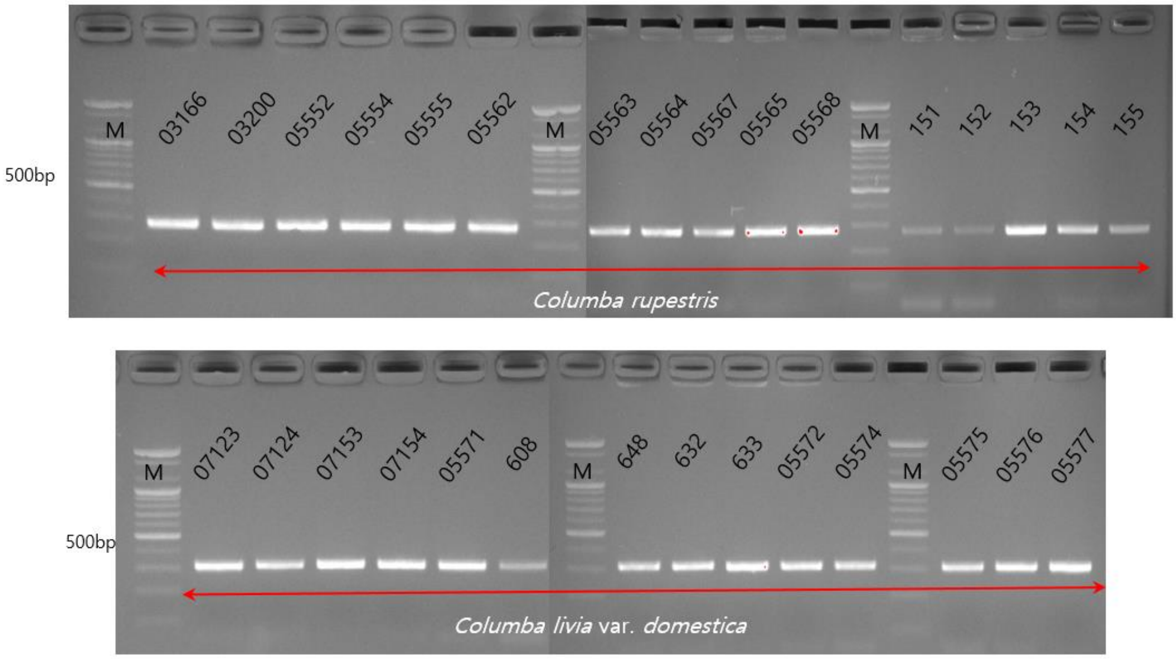

500bp
M 03166 03200 05552 05554 05555 05562 M 05563 05564 05567 05565 05568 M 151 152 153 154 155
Columba rupestris
500bp
M 07123 07124 07153 07154 05571 608 M 648 632 633 05572 05574 M 05575 05576 05577
Columba livia var. domestica

HM 37

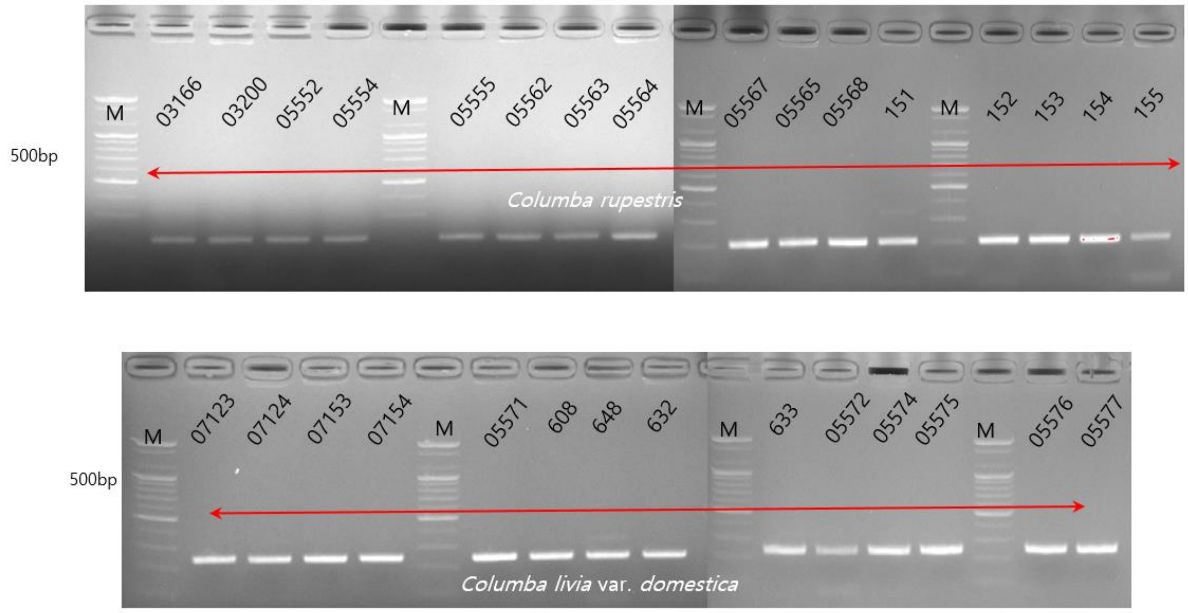
M
03166
03200
05552
05554
M
05555
05562
05563
05564
M
05567
05565
05568
151
M
152
153
154
155
500bp
Columba rupestris
M
07123
07124
07153
07154
M
05571
608
648
632
M
633
05572
05574
05575
M
05576
05577
500bp
Columba livia var. *domestica*

HM 41

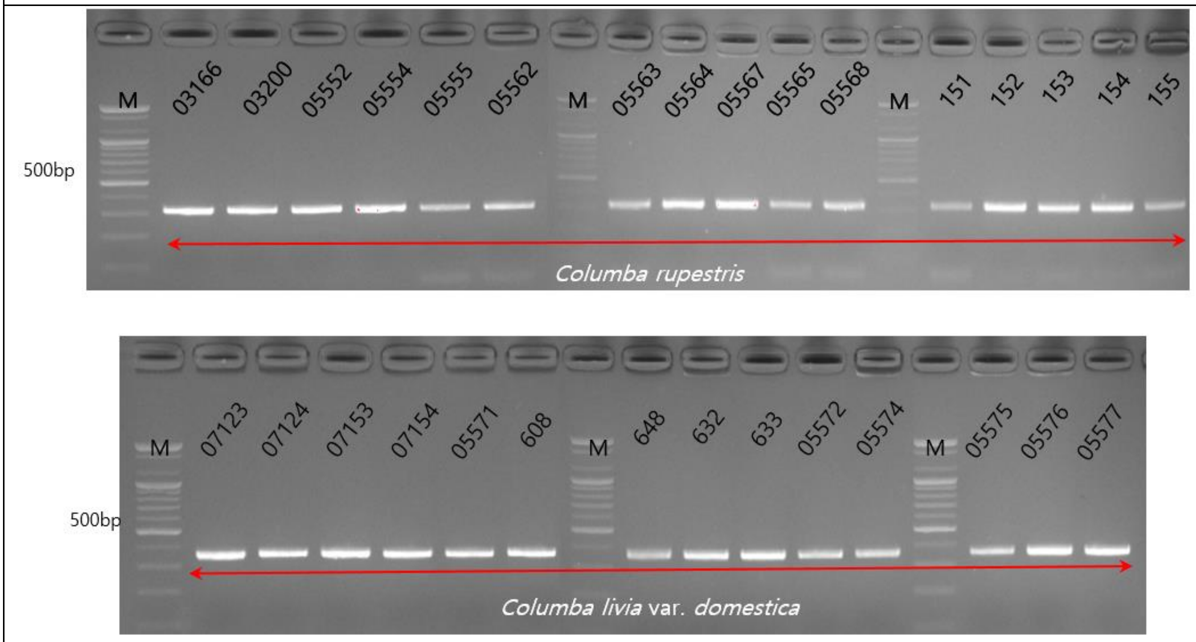
M
03166
03200
05552
05554
05555
05562
M
05563
05564
05567
05565
05568
M
151
152
153
154
155
500bp
Columba rupestris
M
07123
07124
07153
07154
05571
608
M
648
632
633
05572
05574
M
05575
05576
05577
500bp
Columba livia var. *domestica*

HM 42

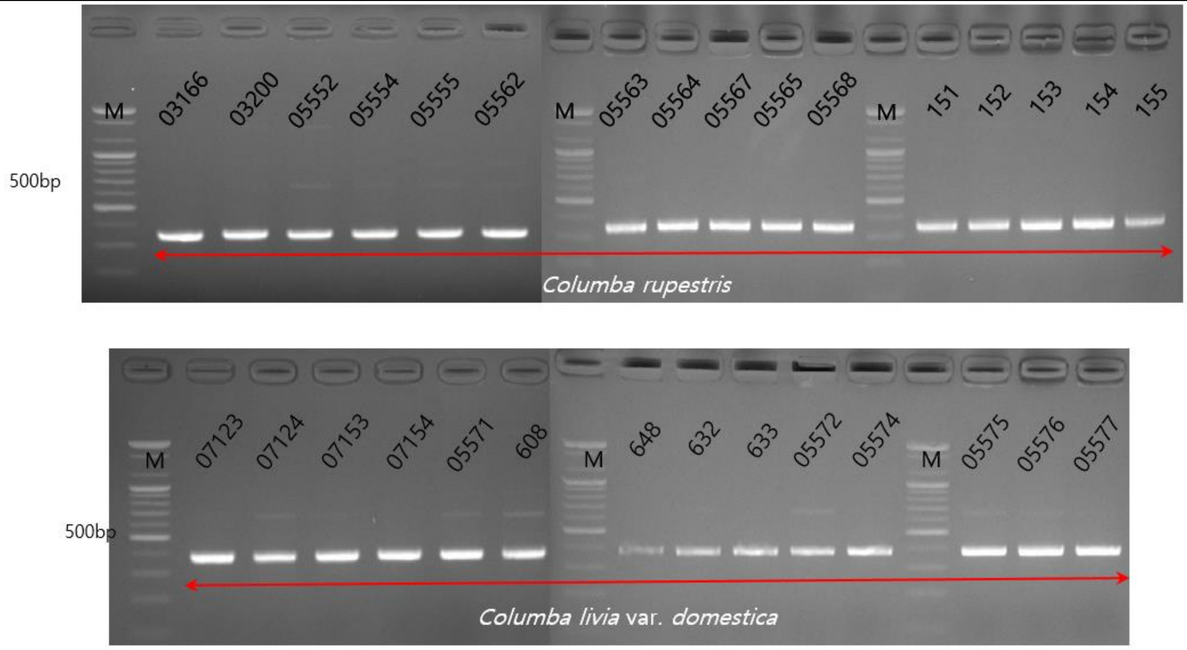

500bp
M
03166
03200
05552
05554
05555
05562
M
05563
05564
05567
05565
05568
M
151
152
153
154
155
Columba rupestris
500bp
M
07123
07124
07153
07154
05571
608
M
648
632
633
05572
05574
M
05575
05576
05577
Columba livia var. domestica

HM 43

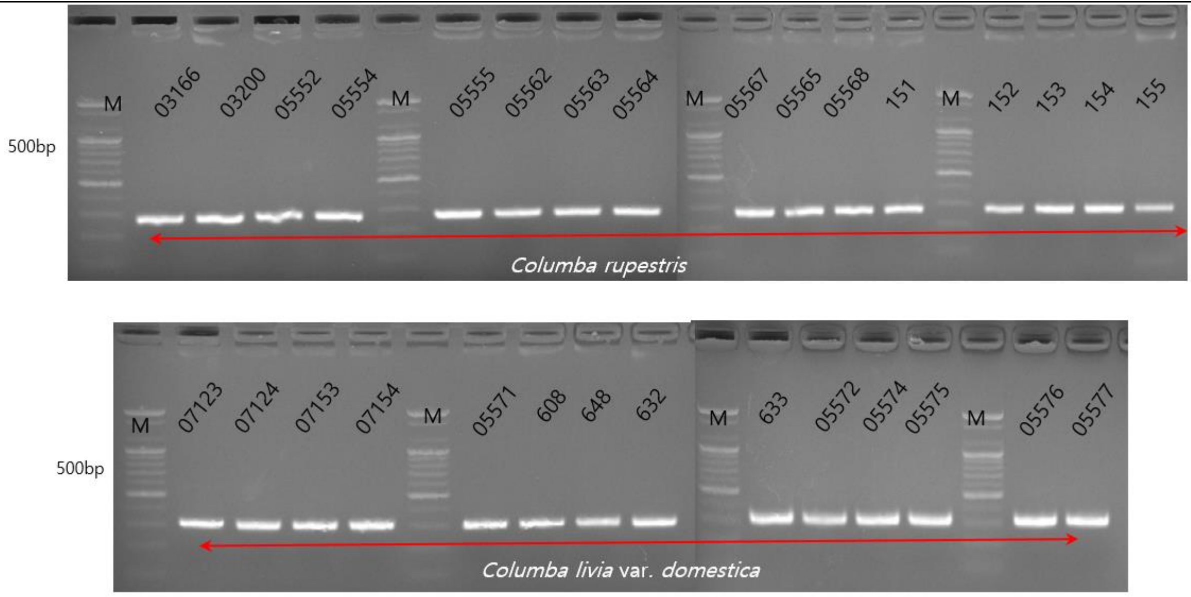

500bp
M
03166
03200
05552
05554
M
05555
05562
05563
05564
M
05567
05565
05568
151
M
152
153
154
155
Columba rupestris
500bp
M
07123
07124
07153
07154
M
05571
608
648
632
M
633
05572
05574
05575
M
05576
05577
Columba livia var. domestica

HM 45

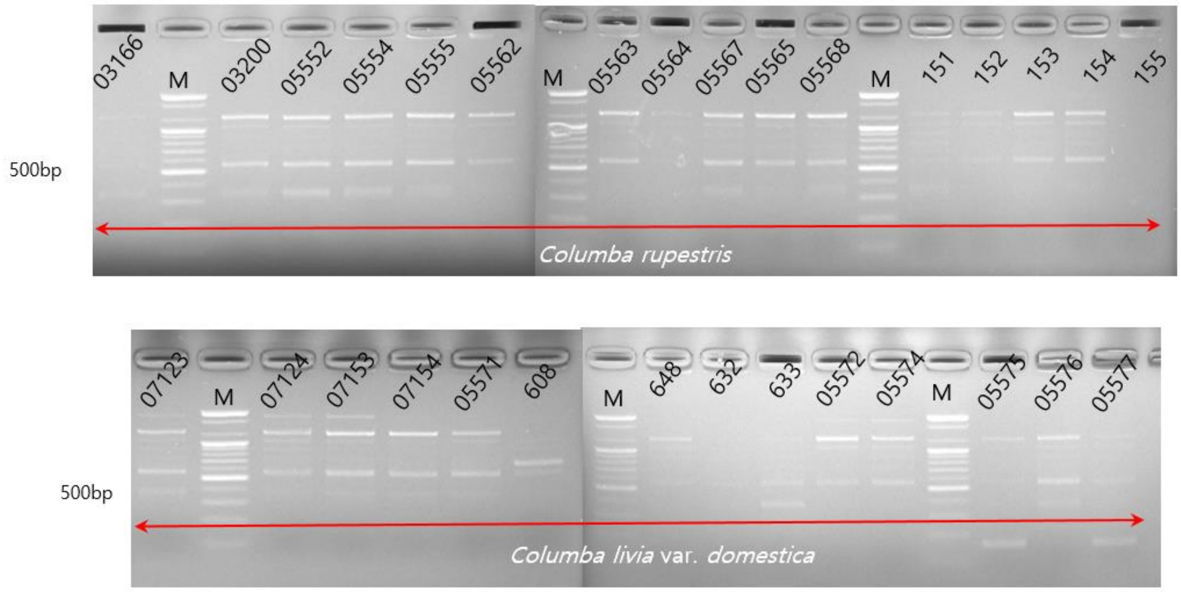
03166 M 03200 05552 05554 05555 05562 M 05563 05564 05567 05565 05568 M 151 152 153 154 155
500bp
Columba rupestris
07123 M 07124 07153 07154 05571 608 M 648 632 633 05572 05574 M 05575 05576 05577
500bp
Columba livia var. domestica

HM 50

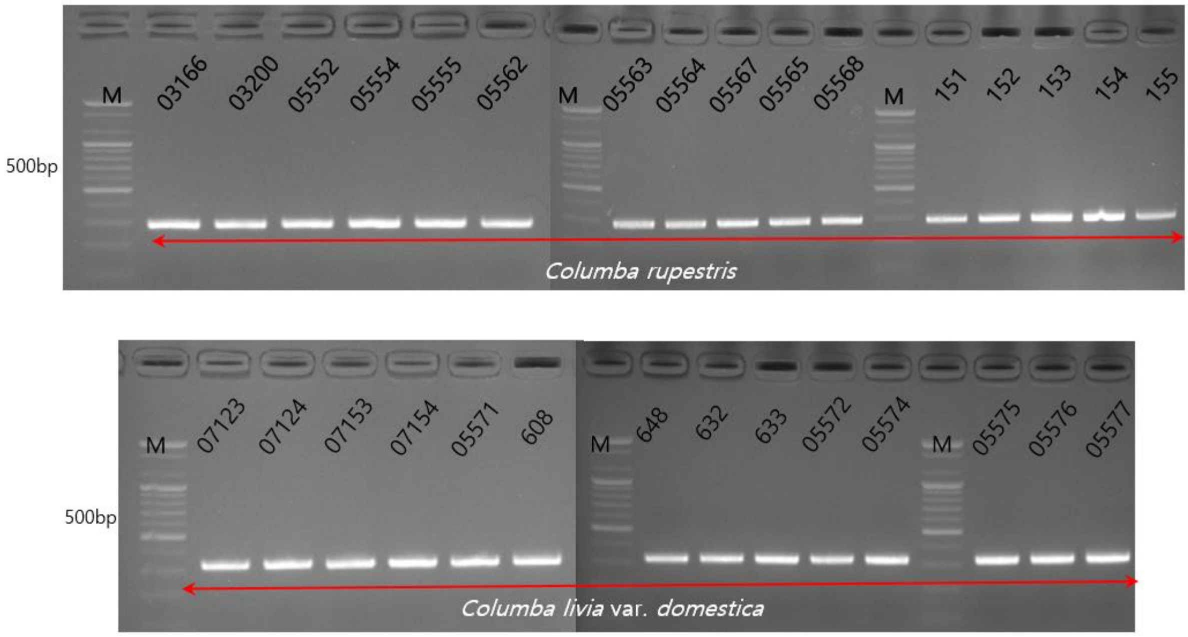
M 03166 03200 05552 05554 05555 05562 M 05563 05564 05567 05565 05568 M 151 152 153 154 155
500bp
Columba rupestris
M 07123 07124 07153 07154 05571 608 M 648 632 633 05572 05574 M 05575 05576 05577
500bp
Columba livia var. domestica

HM 51

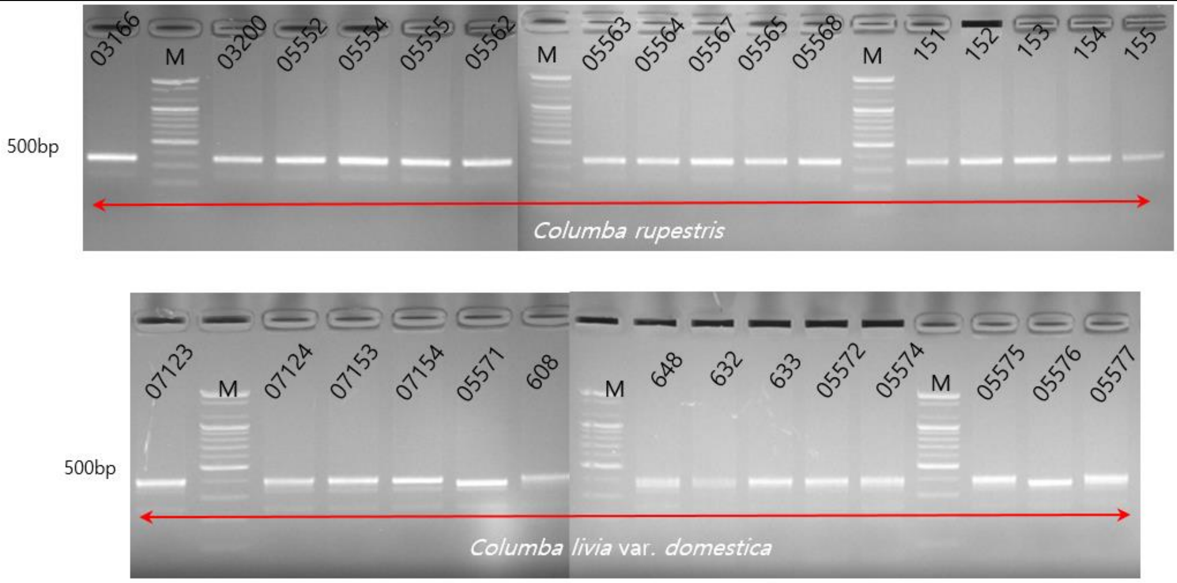
03166 M 03200 05552 05554 05555 05562 M 05563 05564 05567 05565 05568 M 151 152 153 154 155
500bp
Columba rupestris
07123 M 07124 07153 07154 05571 608 M 648 632 633 05572 05574 M 05575 05576 05577
500bp
Columba livia var. domestica

HM 54

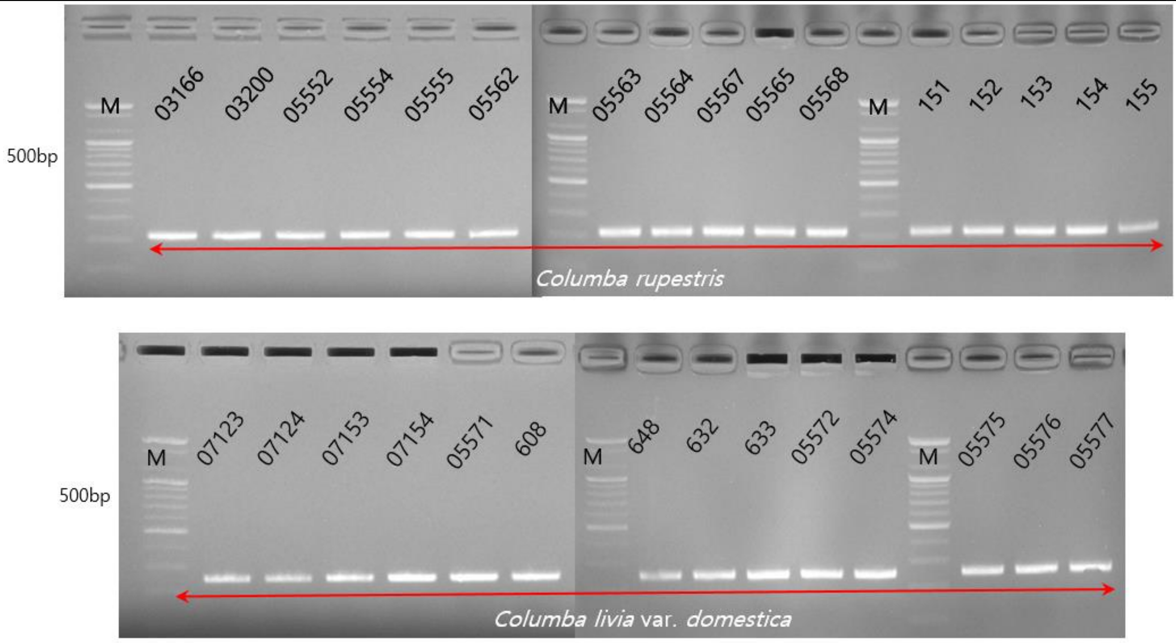
M 03166 03200 05552 05554 05555 05562 M 05563 05564 05567 05565 05568 M 151 152 153 154 155
500bp
Columba rupestris
M 07123 07124 07153 07154 05571 608 M 648 632 633 05572 05574 M 05575 05576 05577
500bp
Columba livia var. domestica

HM 55

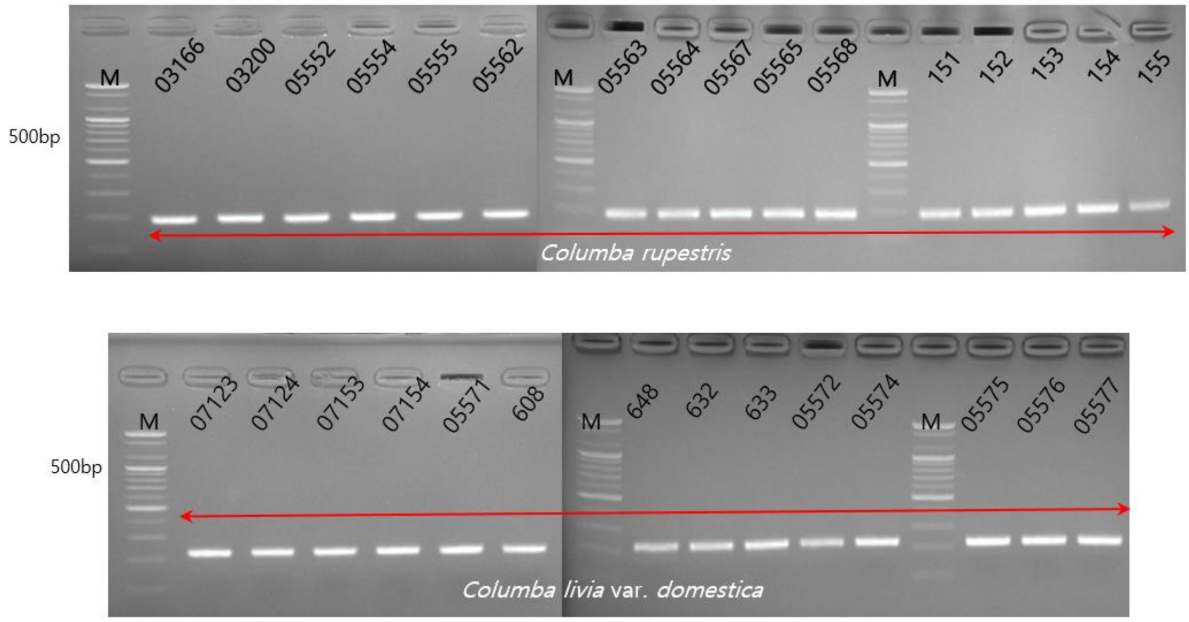
500bp
M
03166
03200
05552
05554
05555
05562
M
05563
05564
05567
05565
05568
M
151
152
153
154
155
Columba rupestris
500bp
M
07123
07124
07153
07154
05571
608
M
648
632
633
05572
05574
M
05575
05576
05577
Columba livia var. domestica

HM 56

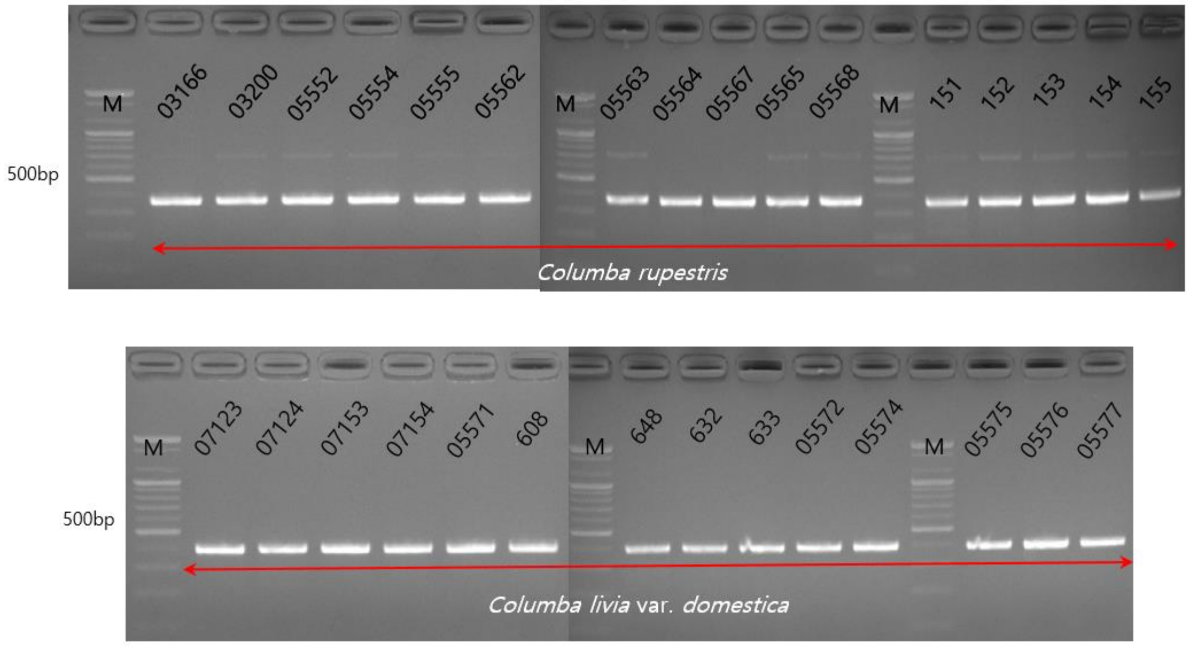
500bp
M
03166
03200
05552
05554
05555
05562
M
05563
05564
05567
05565
05568
M
151
152
153
154
155
Columba rupestris
500bp
M
07123
07124
07153
07154
05571
608
M
648
632
633
05572
05574
M
05575
05576
05577
Columba livia var. domestica

HM 59

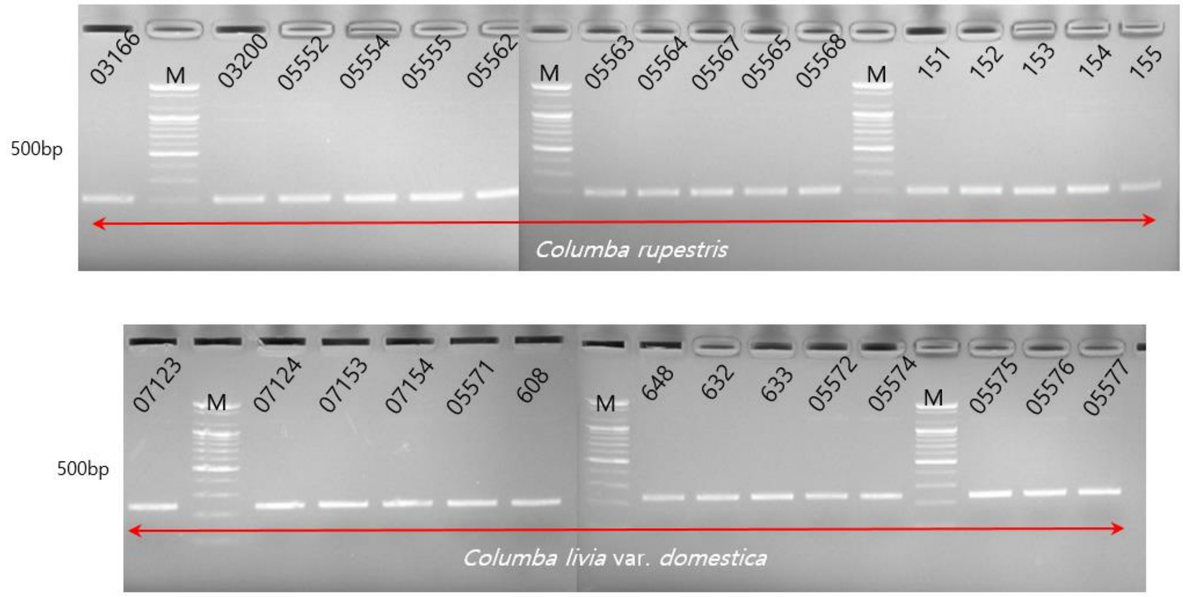
03166 M 03200 05552 05554 05555 05562 M 05563 05564 05567 05565 05568 M 151 152 153 154 155
500bp
Columba rupestris
07123 M 07124 07153 07154 05571 608 M 648 632 633 05572 05574 M 05575 05576 05577
500bp
Columba livia var. domestica

HM 60

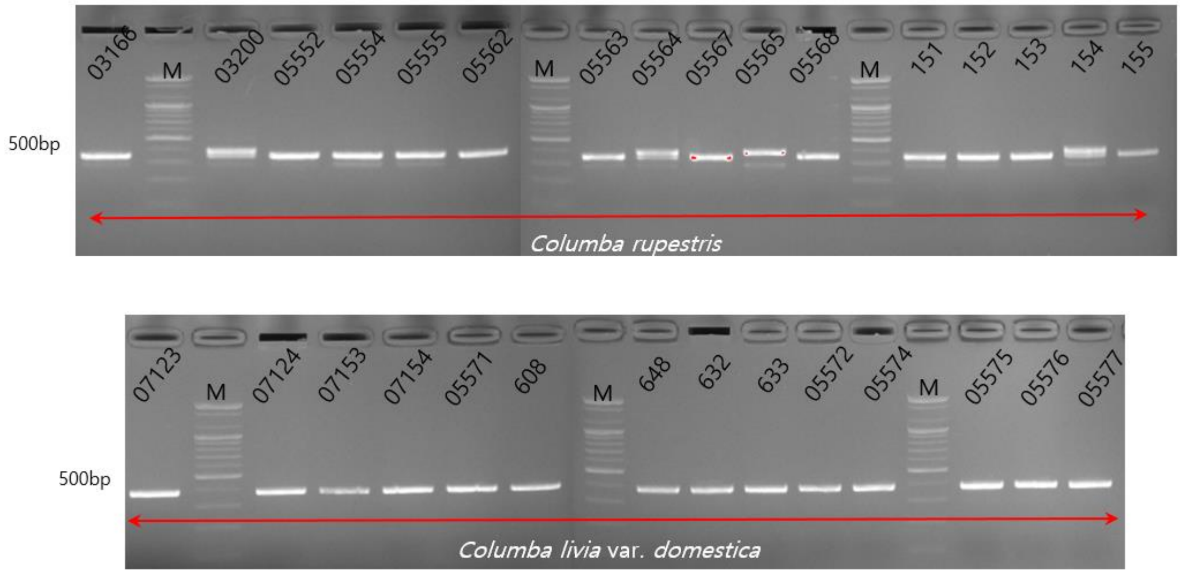
03166 M 03200 05552 05554 05555 05562 M 05563 05564 05567 05565 05568 M 151 152 153 154 155
500bp
Columba rupestris
07123 M 07124 07153 07154 05571 608 M 648 632 633 05572 05574 M 05575 05576 05577
500bp
Columba livia var. domestica

HM 61

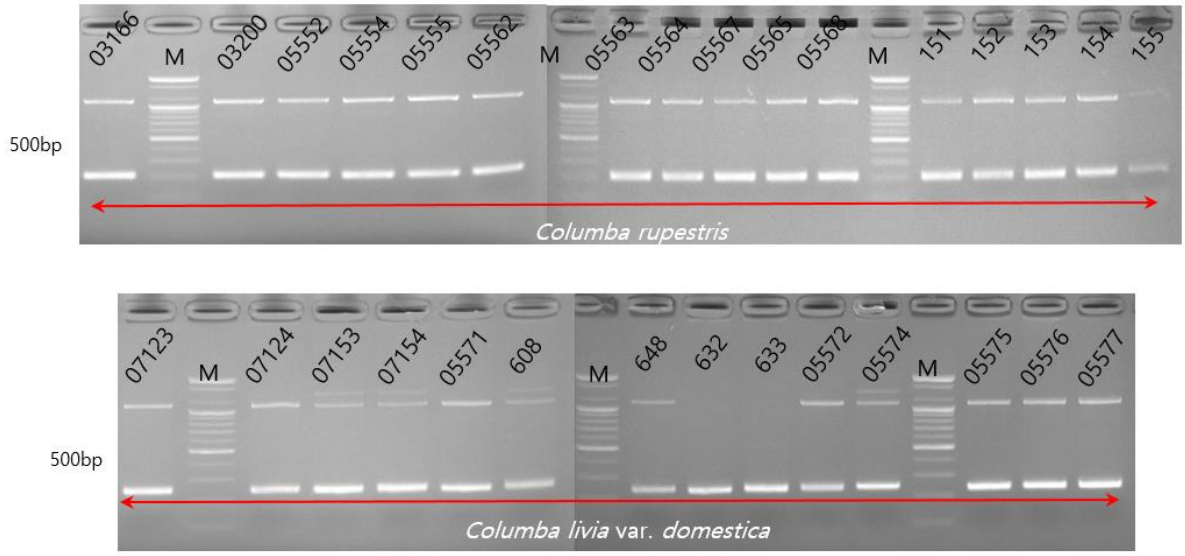

HM 66

Supplementary Figure S3. Test of selected 17 primers to identify species-specific InDel region of hill pigeon (*Columba rupestris*) and feral pigeon (*Columba livia var. domestica*) using agarose gel electrophoresis. Selected primers were indicated in green.

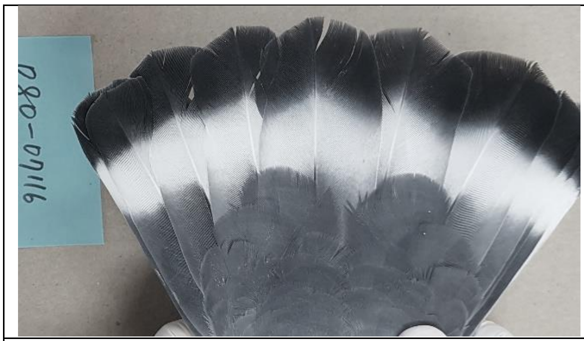	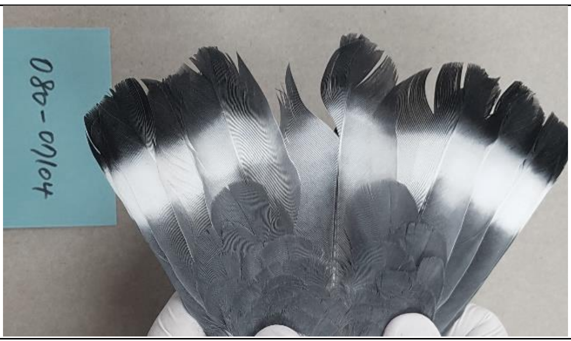
Species: *Columba rupestris*,<br>Sample ID: 07116 (male)	Species: *Columba rupestris*,<br>Sample ID: 07104 (female)
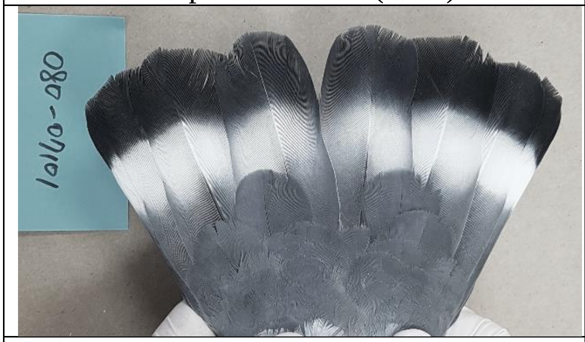	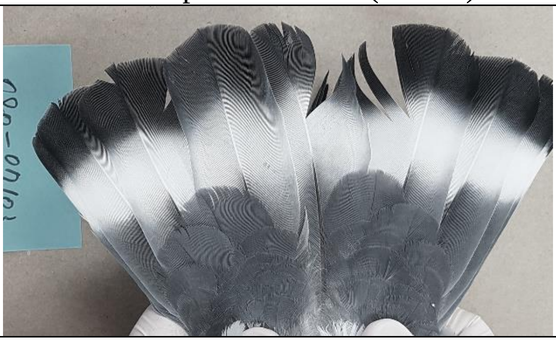
Species: *Columba rupestris*,<br>Sample ID: 07101 (female)	Species: *Columba rupestris*,<br>Sample ID: 07103 (male)
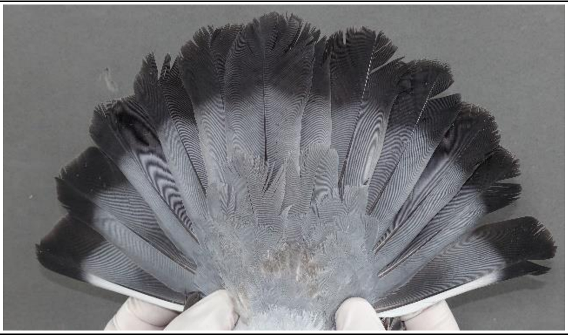	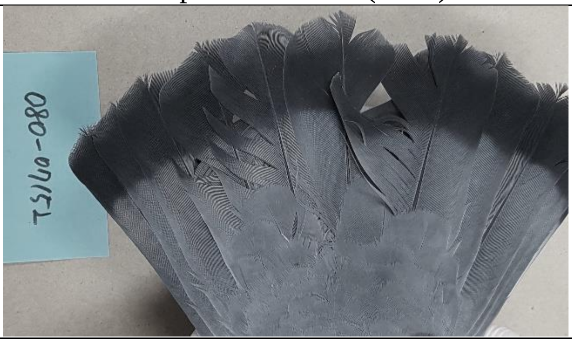
Species: *Columba livia var. domestica*,<br>Sample ID: 06 (female)	Species: *Columba livia var. domestica*,<br>Sample ID: 07125 (male)
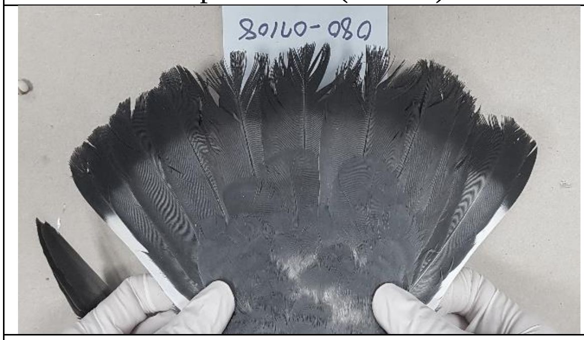	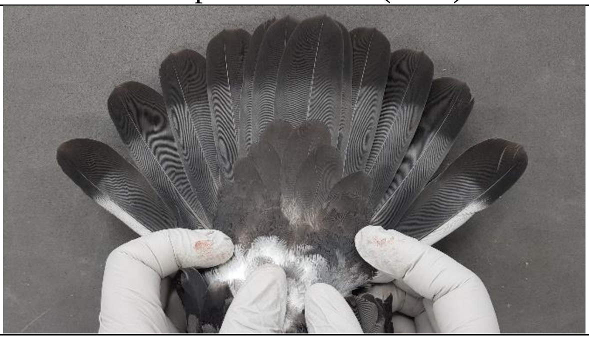
Species: *Columba livia var. domestica*,<br>Sample ID: 07108 (male)	Species: *Columba livia var. domestica*,<br>Sample ID: 07154 (female)

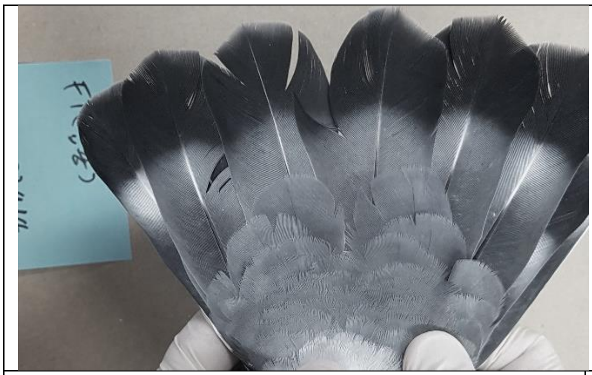	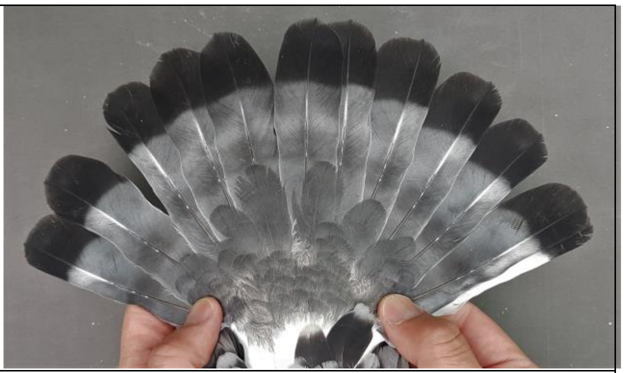
Species: hybrid F1 (07116 x 06),<br>Sample ID: 02414 (male)	Species: hybrid F1 (07103 x 07154)<br>Sample ID: 02422 (male)
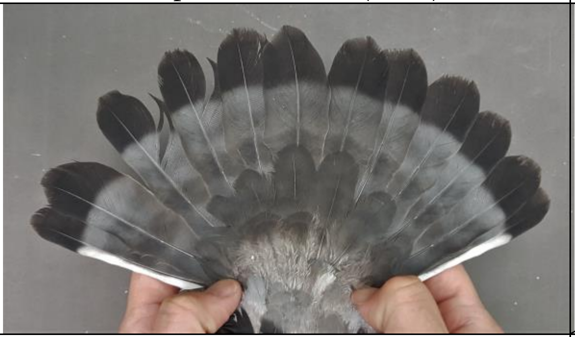	
Species: hybrid F1 (07103 x 07154)<br>Sample ID: 02423 (female)	

Supplementary Figure S4. Phenotypes of hill pigeon, feral pigeon, and their hybrid F1 for testing the hybrid InDel marker.

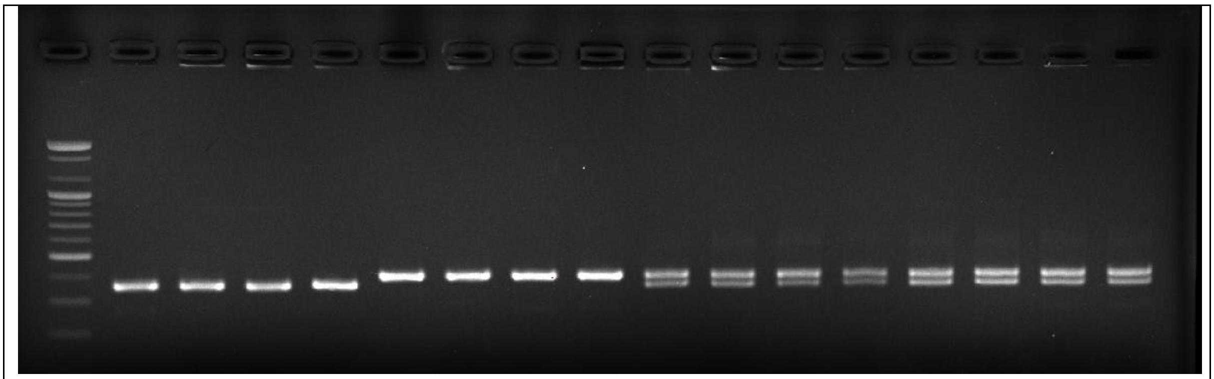
HM 30

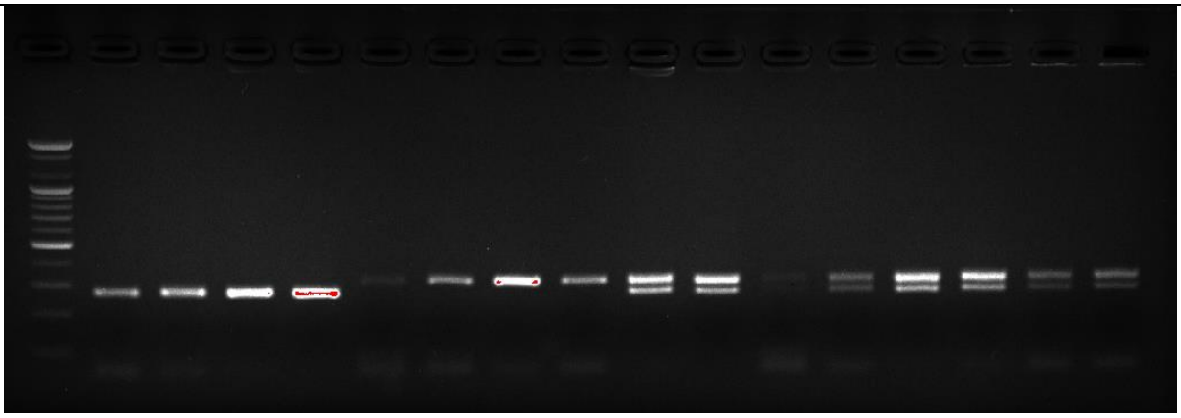
HM37

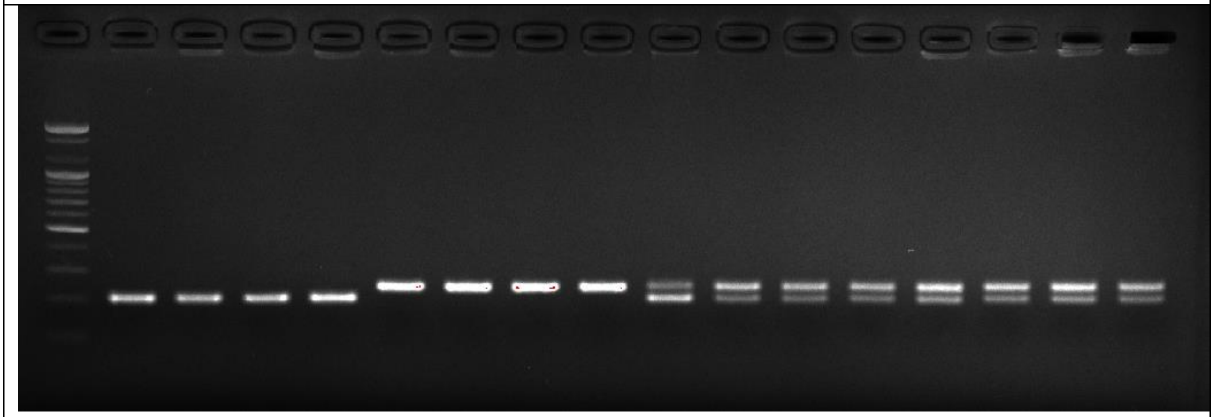
HM41

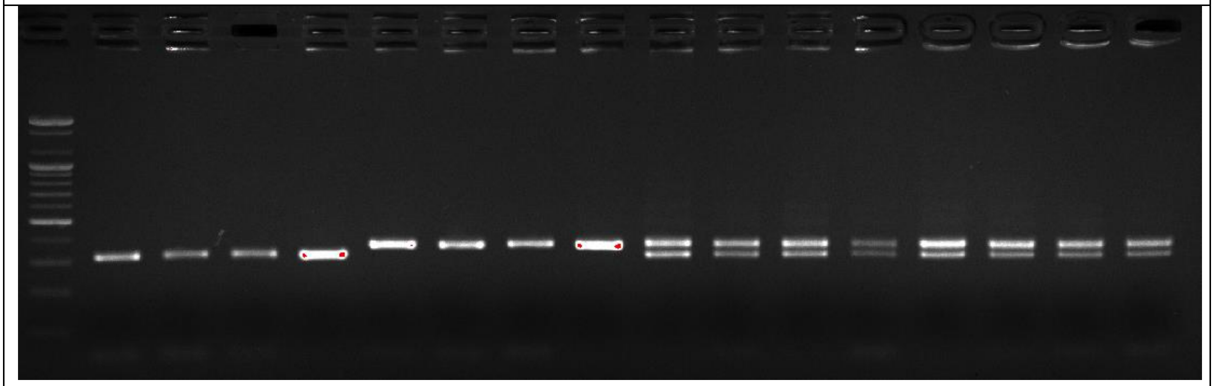
HM42

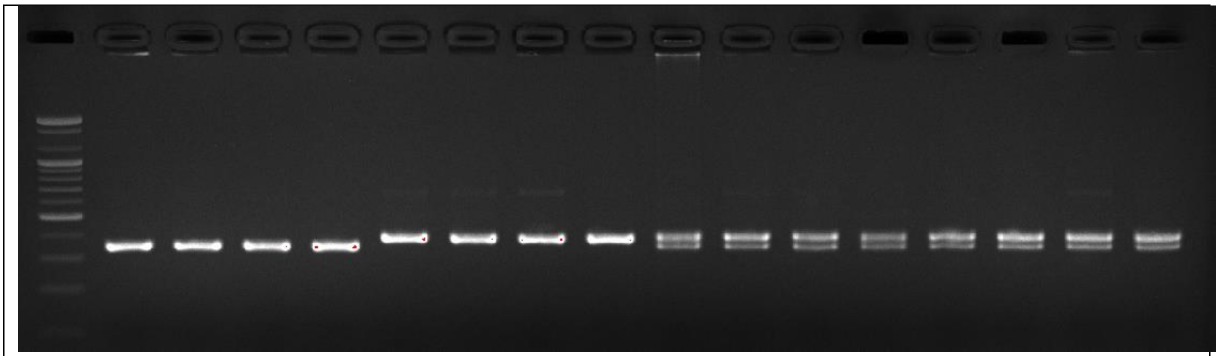

HM43

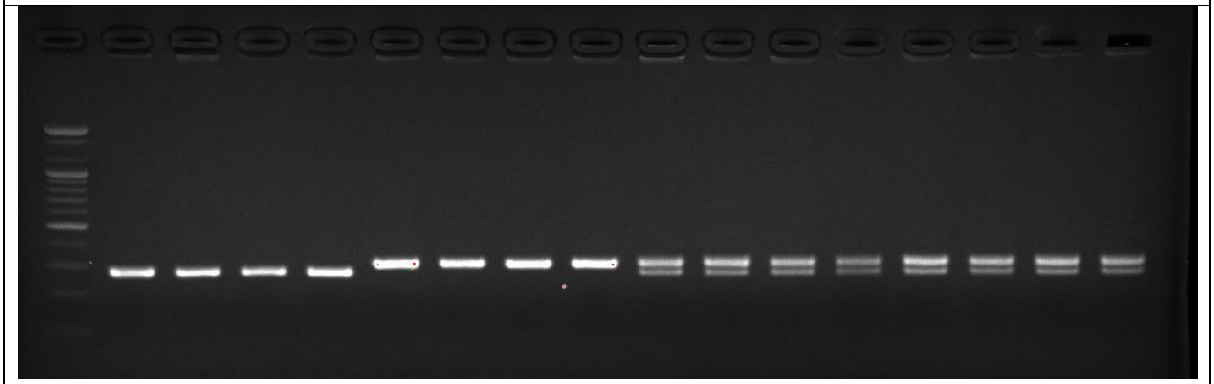

HM45

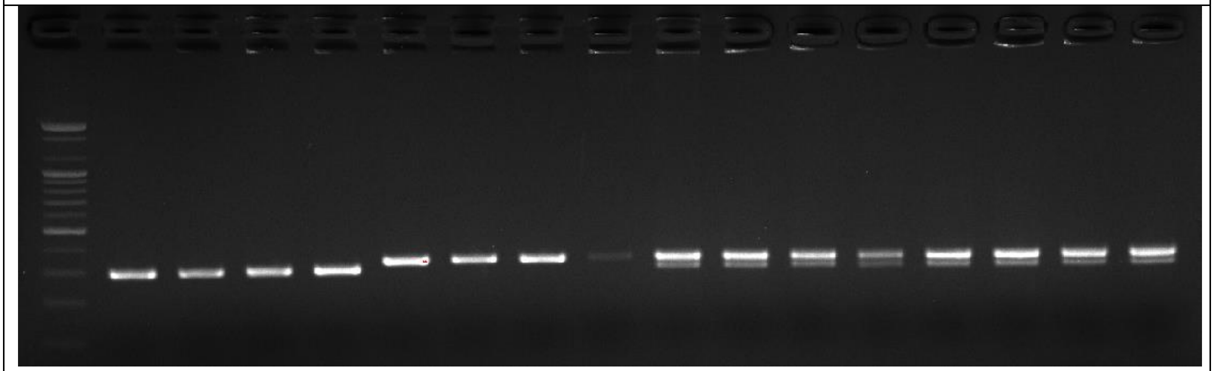

HM51

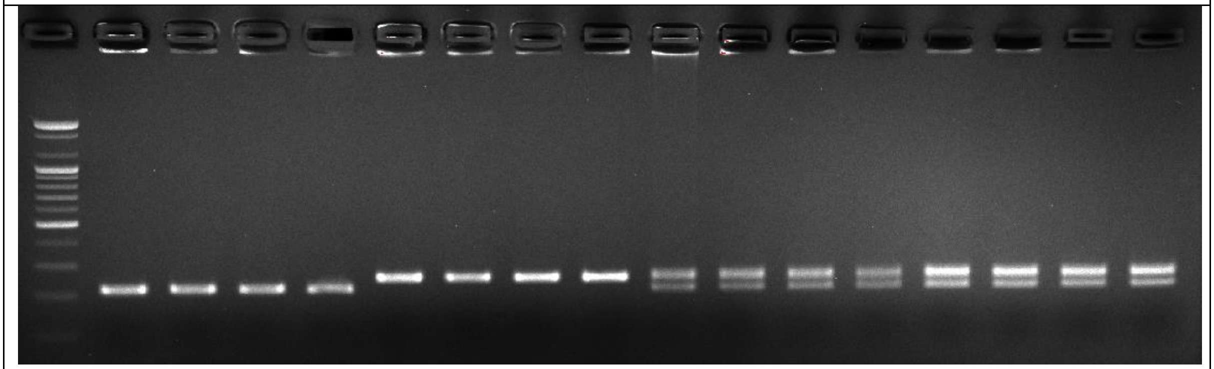

HM55

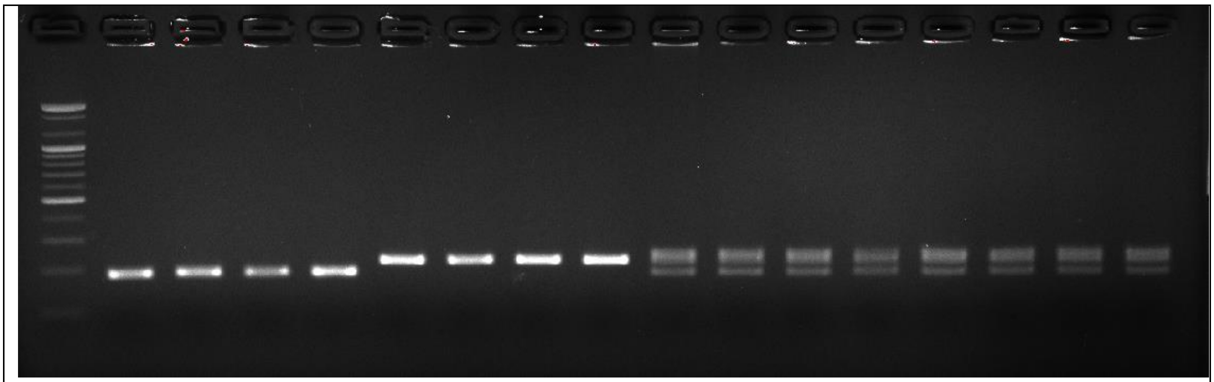
HM56

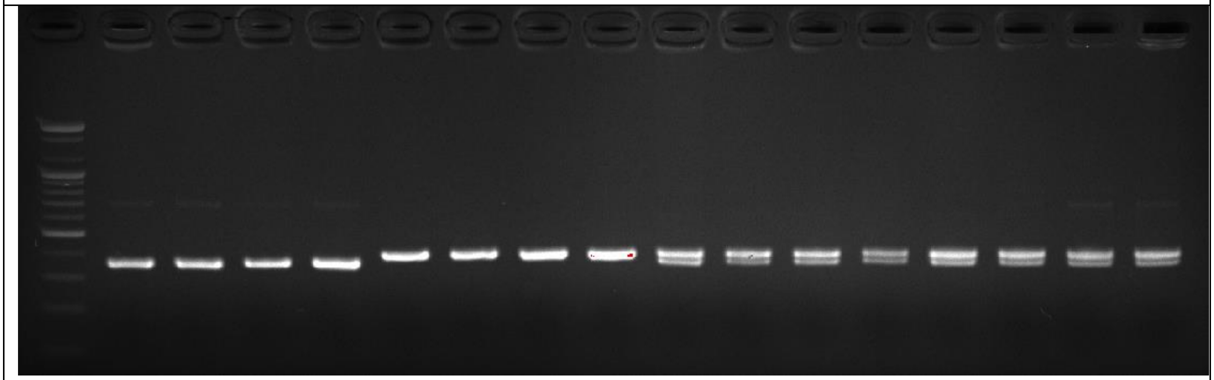
HM59

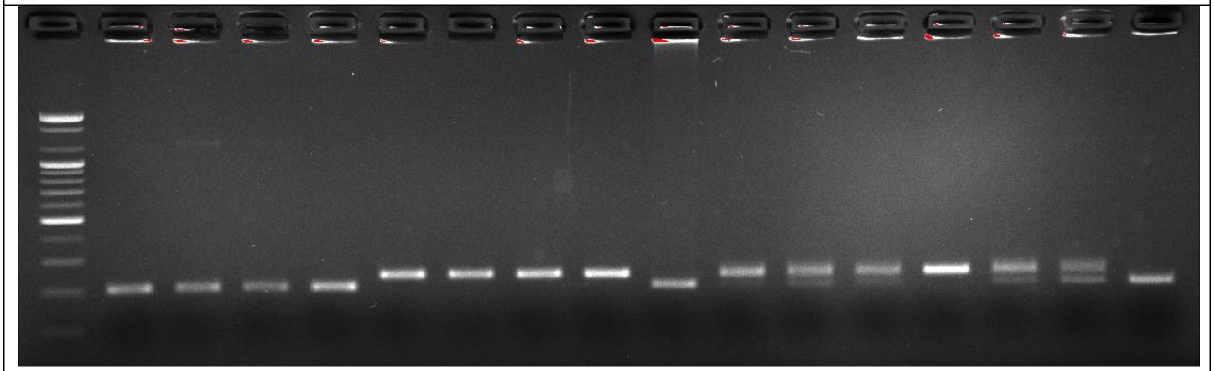
HM60

Supplementary Figure S5. None cropped and original gel image of Figure 1. The name of Individuals and populations were represented in Figure 1.
